# Single-cell transcriptomics reveals multiple chemoresistant properties in leukemic stem and progenitor cells in pediatric AML

**DOI:** 10.1186/s13059-023-03031-7

**Published:** 2023-08-31

**Authors:** Yongping Zhang, Shuting Jiang, Fuhong He, Yuanyuan Tian, Haiyang Hu, Li Gao, Lin Zhang, Aili Chen, Yixin Hu, Liyan Fan, Chun Yang, Bi Zhou, Dan Liu, Zihan Zhou, Yanxun Su, Lei Qin, Yi Wang, Hailong He, Jun Lu, Peifang Xiao, Shaoyan Hu, Qian-Fei Wang

**Affiliations:** 1 Department of Hematology and Oncology, Children’s Hospital of Soochow University, Suzhou, 215025 China; 2 CAS Key Laboratory of Genomic and Precision Medicine, Beijing Institute of Genomics, Chinese Academy of Sciences and China National Center for Bioinformation, Beijing, 100101 China; 3 University of Chinese Academy of Sciences, Beijing, 100049 China; 4 Institute of Pediatric Research, Children’s Hospital of Soochow University, Suzhou, 215025 China; 5 SuZhou Hospital of Anhui Medical University, Suzhou, China

**Keywords:** Residual tumor cell, Single-cell RNA sequencing, AML, Chemotherapy resistance, Leukemia stem cell, Oxidative phosphorylation, HSC-like, *CD69*

## Abstract

**Background:**

Cancer patients can achieve dramatic responses to chemotherapy yet retain resistant tumor cells, which ultimately results in relapse. Although xenograft model studies have identified several cellular and molecular features that are associated with chemoresistance in acute myeloid leukemia (AML), to what extent AML patients exhibit these properties remains largely unknown.

**Results:**

We apply single-cell RNA sequencing to paired pre- and post-chemotherapy whole bone marrow samples obtained from 13 pediatric AML patients who had achieved disease remission, and distinguish AML clusters from normal cells based on their unique transcriptomic profiles. Approximately 50% of leukemic stem and progenitor populations actively express leukemia stem cell (LSC) and oxidative phosphorylation (OXPHOS) signatures, respectively. These clusters have a higher chance of tolerating therapy and exhibit an enhanced metabolic program in response to treatment. Interestingly, the transmembrane receptor *CD69* is highly expressed in chemoresistant hematopoietic stem cell (HSC)-like populations (named the *CD69*^+^ HSC-like subpopulation). Furthermore, overexpression of *CD69* results in suppression of the mTOR signaling pathway and promotion of cell quiescence and adhesion in vitro. Finally, the presence of *CD69*^+^ HSC-like cells is associated with unfavorable genetic mutations, the persistence of residual tumor cells in chemotherapy, and poor outcomes in independent pediatric and adult public AML cohorts.

**Conclusions:**

Our analysis reveals leukemia stem cell and OXPHOS as two major chemoresistant features in human AML patients. *CD69* may serve as a potential biomarker in defining a subpopulation of chemoresistant leukemia stem cells. These findings have important implications for targeting residual chemo-surviving AML cells.

**Supplementary Information:**

The online version contains supplementary material available at 10.1186/s13059-023-03031-7.

## Background

Cancer patients often achieve dramatic responses to chemotherapeutic drugs yet retain therapy-resistant tumor cells, which ultimately results in relapse and decreased patient survival [1]. Chemotherapy serves as a main treatment strategy for acute myeloid leukemia (AML), a neoplastic cancer characterized by the accumulation of aberrant immature cells in the bone marrow (BM). To prevent AML relapse, increasing attention is being paid to leukemia cells that can survive chemotherapy. Although emerging sequencing technology has allowed more sensitive detection of those cells through genetic mutations, the biological characteristics of chemoresistant cells in AML patients remain largely unknown [2, 3].

Mouse model studies employing patient pre-chemotherapy samples have proposed that leukemia stem cells (LSCs) with self-renewal properties can preferentially survive chemotherapy [4]. Nevertheless, this prediction is mainly based on their inherent dormancy and has not been demonstrated in post-treatment patients [5]. In contrast, accumulating evidence using xenograft models with cytarabine treatment has found that chemoresistant properties were not associated with LSCs but resided in cells with active oxidative phosphorylation (OXPHOS), chemo-induced leukemia regenerating cells (LRC) or senescence-like cells [6–8]. However, to what extent AML patients exhibit these cellular and molecular features remains largely unknown.

Single-cell RNA sequencing (scRNA-seq) has emerged as a powerful tool for revealing tumor heterogeneity and identifying subpopulations with distinct molecular signatures [9]. Here, we applied scRNA-seq to paired pre- and post-chemotherapy whole BM samples from AML patients to maximize the ability to detect leukemic cells and evaluate their chemoresistant potential. We developed an efficient strategy to distinguish leukemic and normal cells based on their transcriptomes. Our analysis identified leukemic cell populations with distinct chemoresistant transcription features. Remarkably, we identified a quiescent *CD69*^+^ HSC-like subpopulation with stem and adhesion characteristics that could survive after chemotherapy. The clinical relevance of this subpopulation was further determined by deconvolution analysis of two publicly available cohorts. Collectively, our study provided the first in vivo characterization of post-therapy tumor heterogeneity in AML patients and identified a key cell population that may convey chemoresistance and drive disease recurrence.

## Results

### Single-cell baseline transcriptome landscape of human normal hematopoiesis

To gain insight into the cellular diversity of leukemic cells, we first profiled the baseline cellular diversity in normal hematopoiesis for comparison. We applied high-throughput 10X Genomics scRNA-seq to profile 72,624 cells from nine healthy BM and peripheral blood (PB) samples, including 40,326 CD34^+^-enriched cells, to investigate stem and progenitor populations. We also integrated three publicly available scRNA-seq datasets with 82,950 cells to capture a broad representation of hematopoietic cell types (Additional file 1: Fig. S1a; Additional file 2: Table S1) [10–12]. In total, 155,574 high-quality cells from thirty-one samples from healthy donors were combined for downstream analysis.

**Fig. 1:**
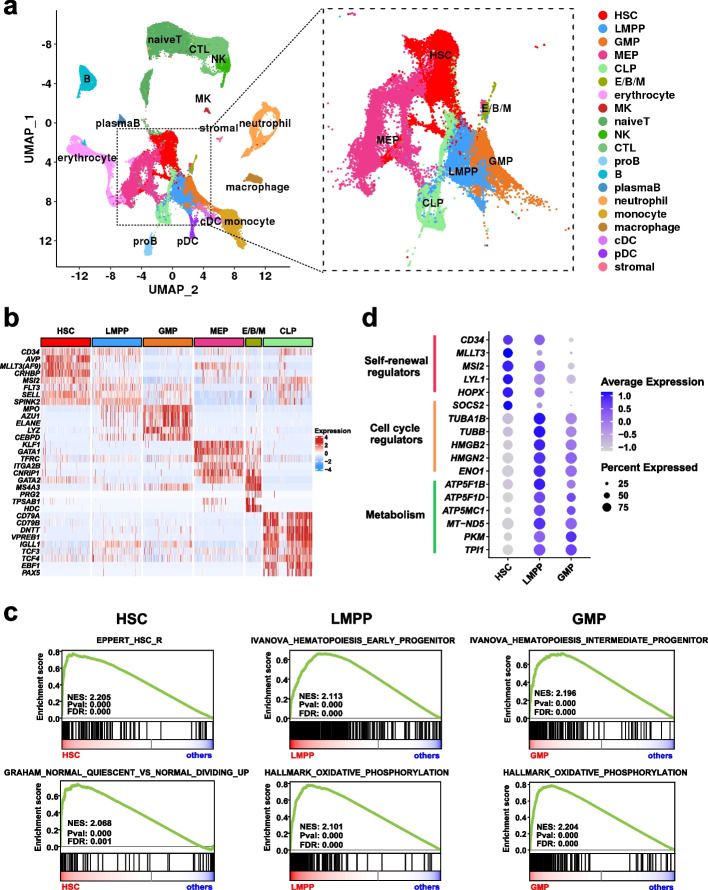
Single-cell transcriptome landscape of human normal hematopoiesis. **a** UMAP visualization of healthy human hematopoietic cells (*n* = 155,574 cells), with each dot representing a cell and colors indicating distinct cell types. The inset plot provides an enlarged view of the six HSPC clusters, including HSC (hematopoietic stem cell), LMPP (lymphoid-primed multi-potential progenitor), GMP (granulocyte–macrophage progenitor), CLP (common lymphoid progenitor), MEP (megakaryocyte (MK) and erythroid progenitor), and E/B/M (eosinophil/basophil/mast cell progenitor) that express three lineages-specific canonical markers and MEP commitment-essential transcription factors, consistent with previous reports [14–17]. **b** Heatmap illustrating cell type-specific gene expression (rows) across various HSPC populations (columns). **c** GSEA plots showing the representative gene signatures enriched in HSC, LMPP, and GMP populations with accompanying normalized enrichment score (NES), *p* value, and false discovery rate (FDR) value. **d** Dot plot representing the expression of representative genes involved in indicated biological processes in HSC, LMPP, and GMP populations. Dot size signifies the proportion of cells expressing a gene in a cell population, while shading represents the relative expression level

After removing the batch effect, unsupervised clustering was performed, and the results were visualized by uniform manifold approximation and projection (UMAP) (see “Material and methods”; Additional file 1: Fig. S1b) [13]. Twenty cell types were inferred according to well-known cell type-specific genes, including six hematopoietic stem and progenitor cell (HSPC) populations as well as multiple myeloid, lymphoid, megakaryocyte, and erythroid populations (Fig. 1a-b and Additional file 1: Fig. S1c; see “Material and methods”). Our cell type annotations were consistent with recent scRNA-seq studies and published gene signatures (Additional file 1: Fig. S1d).

We further focused on the transcriptional characteristics of three cell types along the hematopoietic stem cell (HSC) to myeloid progenitor axis, including HSC, lymphoid-primed multi-potential progenitor (LMPP), and granulocyte–macrophage progenitor (GMP) (*n* = 26,423 cells). Gene set enrichment analysis (GSEA) revealed that HSCs possessed expression signatures enriched for stemness and quiescence, while LMPPs and GMPs exhibited increased proliferation and OXPHOS expression (Fig. 1c). In agreement with the enrichment analysis, HSCs overexpressed genes related to stem cell function, including self-renewal regulators (*CD34*, *MLLT3*, *MSI2*, *LYL1*, *HOPX*) and cell cycle regulators (*SOCS2*) (Fig. 1d and Additional file 3: Table S2). In contrast, both LMPPs and GMPs highly expressed genes that were involved in cell cycle progression (*TUBA1B*, *TUBB*, *HMGB2*, *HMGN2*, *TUBA1B*, *ENO1*), DNA replication, and metabolism pathways including ATP synthase and NADH dehydrogenase. GMPs also highly expressed granule genes such as *AZU1* and *ELENE* (Fig. 1d and Additional file 3: Table S2). In addition, cell cycle state prediction analysis further confirmed that HSCs contained a higher proportion of cells in a resting cell cycle state (G0/G1 phase) than LMPPs and GMPs (Additional file 1: Fig. S1e). Interestingly, quiescence, stemness, and OXPHOS features have been associated with chemoresistance properties in leukemia cells [18].

Overall, we revealed that HSPC populations at different developmental stages had distinct molecular characteristics that are relevant to chemoresistance properties in AML. In addition, the normal hematopoietic landscape serves as an important reference for distinguishing leukemic cells and understanding their heterogeneity.

### Identification and validation of AML cells from pre- and post-chemotherapy whole bone marrow populations

**Fig. 2:**
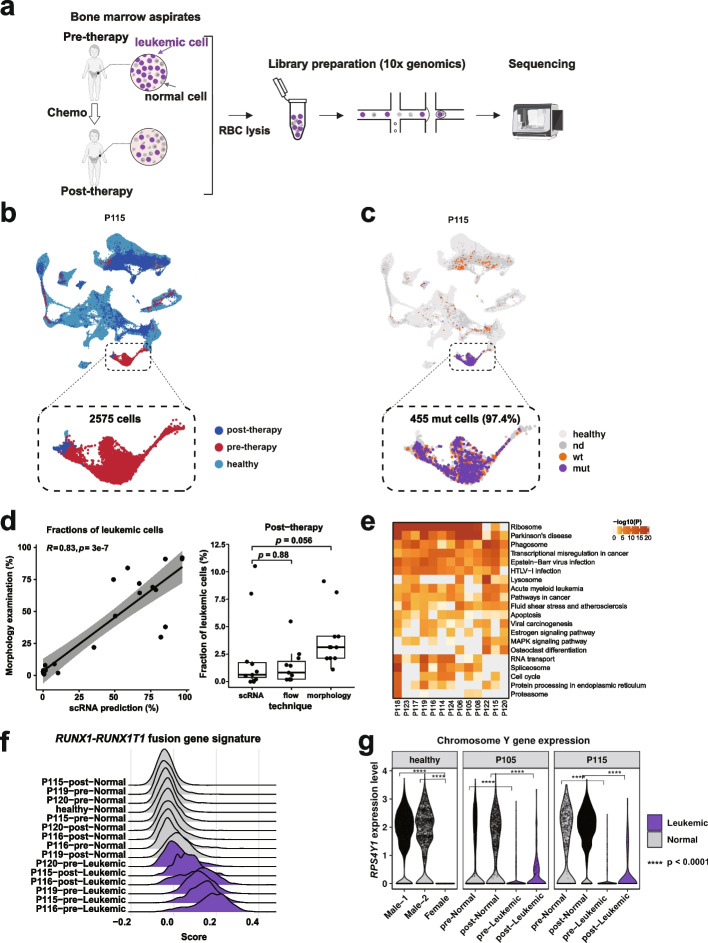
Identification and validation of AML cells in pre- and post-chemotherapy whole bone marrow samples. **a** Workflow illustrating the collection and processing of BM aspirates from 13 pediatric AML patients for scRNA-seq analysis. **b** UMAP shows the clustering of healthy donors (*n* = 155,574 cells) with paired pre- and post-therapy samples from patient P115 (*n* = 18,257 cells). Cells are color-coded by sample origin. The inset plot provides an enlarged view of leukemia clusters, with the predicted leukemic cell count indicated. **c** UMAP visualization as in panel b, with cells colored based on detected mutant (purple) or wild-type (orange) transcripts. The number of mutant cells is indicated, and the percentage of mutant cells assigned to predicted leukemia cells is noted in parentheses. **d** (Left) Scatterplot comparing the proportions of predicted malignant cells determined by morphology and scRNA-seq, with correlation coefficient (R) and *p* values calculated using Pearson’s correlation test. (Right) Boxplot comparing the proportions of post-therapy malignant cells determined by scRNA-seq, morphology, and flow cytometry, with each point representing a sample and *p* values calculated using the Wilcoxon signed-rank test. **e** Heatmap displays KEGG pathways enriched by highly expressed genes in leukemic cells within each AML patient. **f** Ridge plots showing the expression of *RUNX1*-*RUNX1T1* fusion gene signature in leukemic and normal cells from four patients (P115, P116, P119, and P120) harboring this chromosomal translocation. **g** Violin plots depicting the expression of Y chromosome-located gene *RPS4Y1* in cells from healthy female and male donors, as well as in predicted leukemic and normal cells pre- and post-chemotherapy from two patients (P105 and P115) with a chromosome Y deletion. *P* values were calculated using the Wilcoxon signed-rank test

There is currently a lack of universal markers for the prospective isolation of leukemic cells. To maximize the power to detect leukemic cells from a mixed population of leukemic and normal cells, we used unsorted whole BM samples for high-throughput 10X Genomics scRNA-seq. Twenty-six BM samples collected at two time points (pre- and post-chemotherapy) from thirteen remission pediatric AML patients were sequenced (Fig. 2a and Additional file 4: Table S3). Overall, we retained 227,842 high-quality cells for downstream analyses, with an average of 8,763 cells per sample (range: 2,647–17,512; Additional file 4: Table S3). Approximately 9,986 cells per post-chemotherapy sample were analyzed for the identification of residual leukemic cells.

Previous studies have shown that leukemic cells within high tumor burden samples could be distinguished from normal cells based on their distinct transcriptomic programs [19, 20]. We reasoned that a high tumor burden diagnostic sample could serve as an anchor, and the AML cells with low abundance presented in the remission samples could be reliably identified by coclustering with the isolated leukemic cell populations from diagnostic samples. We first tested the feasibility of this approach in a published dataset in which both high quality scRNA-seq and mutational genotyping data were available [21]. By compiling scRNA-seq data from healthy donors and seven patients with matched pre- and post-therapy samples, we found that pre-therapy cells in all seven patients formed separate clusters away from healthy donors. Noticeably, in three patients with identifiable post-therapy malignant cells (AML7070B, AML328, and AML329), a small proportion of post-therapy cells coclustered with pre-therapy AML cells (Additional file 1: Fig. S2a). The predicted malignant cells derived from our transcriptomic clustering were in high agreement with previous classifications using a machine learning classifier based on integrated genomic and transcriptional information in a published study (*R* = 0.9; Additional file 1: Fig. S2b) [22]. Specifically, 78.04% (range: 47.50%-98.63%) of post-therapy malignant cells assigned by the previous study were also classified as malignant cells in our analysis, while few cells were identified as malignant cells in post-therapy samples where the previous study detected no AML cells. Overall, these data showed that our approach was able to identify malignant cells, especially from patient specimens with rare malignant cells.

We further applied this method to classify leukemic cells in pre- and post-chemotherapy samples from our patient cohort. Based on the morphology and flow cytometry examination, our untreated pre-therapy samples had a high tumor burden, with an estimated average of 64.76% leukemia cells (range: 22%-92%), while post-chemotherapy BM samples predominantly showed enrichment of normal cells (> 95%) and exhibited an average of 3.58% leukemia cells (range: 1%-9%; Additional file 4: Table S3). We integrated scRNA-seq data from thirty-one healthy donors and paired pre- and post- therapy samples from each patient, and performed UMAP projection (Fig. 2b and Additional file 1: Fig. S2c). Our analysis identified two types of major clusters: one almost entirely consisted of normal healthy donor cells, while the other mainly comprised cells derived from pre-therapy samples. Interestingly, a small proportion (~ 1.85%) of post-therapy cells colocalized with the pre-therapy clusters, indicating that these post-therapy cells were residual leukemia cells that survived chemotherapy. We defined a cluster as leukemic if more than 80% of the cells in this cluster were derived from pre-therapy samples and exhibited a close relationship with myeloid cells, while cells in the remaining clusters were classified as normal cells (see “Material and methods”, Additional file 1: Fig. S2d). Overall, we identified an average of 5,481 (range: 2,381–12,433) leukemic cells per diagnostic sample and an average of 152 (range: 5–1,262) leukemic cells per post-treatment sample based on transcriptional profiling (Additional file 4: Table S3). These transcriptionally predicted leukemic cells averagely accounted for 71.43% and 1.85% of total pre- and post-therapy cells, respectively. These data were highly consistent with clinical blast counts estimated by morphology and flow cytometry analysis (Fig. 2d; Additional file 1: Fig. S2e).

Interestingly, the genes overexpressed in transcriptionally predicted leukemic cells were found to be associated with activation of MYC, SATB1, and TAL1, as well as repression of CEBPA, SPI1 (PU.1), FOXC1, NLRC5, and NONO. Most of these genes were hematopoietic lineage-specific transcription factors, indicating that the healthy hematopoietic process was repressed in those leukemic cells (Additional file 1: Fig. S2f). Kyoto Encyclopedia of Genes and Genomes (KEGG) enrichment analysis showed that those leukemic cells had high activities of pathways such as ribosome, transcriptional misregulation in cancer, pathways in cancer, and hematopoietic cell lineage (Fig. 2e). These results were consistent with the distinct transcriptomic programs observed in malignant AML cells from a previous scRNA-seq study [14].

To independently validate these results, we examined the presence of somatic mutations, expression signatures associated with chromosomal structural changes (translocation or chromosome deletion), and the coexpression of leukemia-associated immunophenotype (LAIP) markers in these transcriptionally predicted leukemic cells. First, targeted DNA sequencing was used to identify high-confidence somatic mutations (see “Material and methods”). Cells expressing the somatic mutations were identified using the scRNA-seq data (see “Material and methods”; Fig. 2c and Additional file 4: Table S3). This analysis enabled identification of the fraction of leukemic cells that harbored somatic mutations in proximity to the 3’ end of the gene. An average of 148 (range: 9–455) pre- and post-therapy mutant cells were identified per patient (Additional file 4: Table S3). More than 93% of those mutant cells in each sample were transcriptionally predicted to be leukemic cells (Fig. 2c and Additional file 1: Fig. S2c). Second, patients with chromosomal alterations (four patients with *RUNX1-RUNX1T1* fusions and two patients with a Y chromosome deletion, with patient P115 concurrently carrying these two genomic lesions) were validated by specific gene expression signatures for leukemic cells derived from pre- and post-chemotherapy. In four patients with *RUNX1*-*RUNX1T1* fusion gene, we examined the fusion target gene score of each cell based on the expression of known signature genes (see “Material and methods”) [23]. It was apparent that these genes were preferentially expressed at higher levels in leukemic cells from three patients (P115, P116, and P119; Fig. 2f). Additionally, in two patients (P105 and P115) who harbored a Y chromosome deletion, Y chromosome transcripts were minimally detected in leukemic cells (Fig. 2g). Notably, three patients (P105, P115, and P116) were transcriptionally predicted to have more than 100 post-treatment leukemic cells. Those cells expressed significantly higher levels of *RUNX1-RUNX1T1* fusion transcripts, as well as the fusion gene-associated expression signatures (P115 and P116; Fig. 2c, f and Additional file 1: Fig. S2g). In patients (P105 and P115) who had a Y chromosome deletion, the predicted residual leukemic cells minimally expressed Y chromosome transcripts (Fig. 2g). Third, flow cytometry was used to identify leukemia cells with LAIP expression as previously described [24]. Eleven out of thirteen patients had suitable expression of LAIP markers for defining and monitoring leukemia cells at pre- and post-therapy (Additional file 4: Table S3). Cells coexpressing LAIP markers were identified using the scRNA-seq data of these eleven patients (see “Material and methods”, Additional file 4: Table S3). We observed that 94.73% of those cells were classified as transcriptionally defined leukemia cells, while only 5.27% were classified as transcriptionally defined normal cells (Additional file 1: Fig. S2c).

Together, these data indicate that we were able to confirm the identification of the transcriptionally predicted leukemic cells from all thirteen patients using at least one independent method (Additional file 1: Fig. S2i).

### LSC and OXPHOS signatures were prevalent in leukemic stem and progenitor populations and persistent in drug-resistant subsets after chemotherapy

**Fig. 3:**
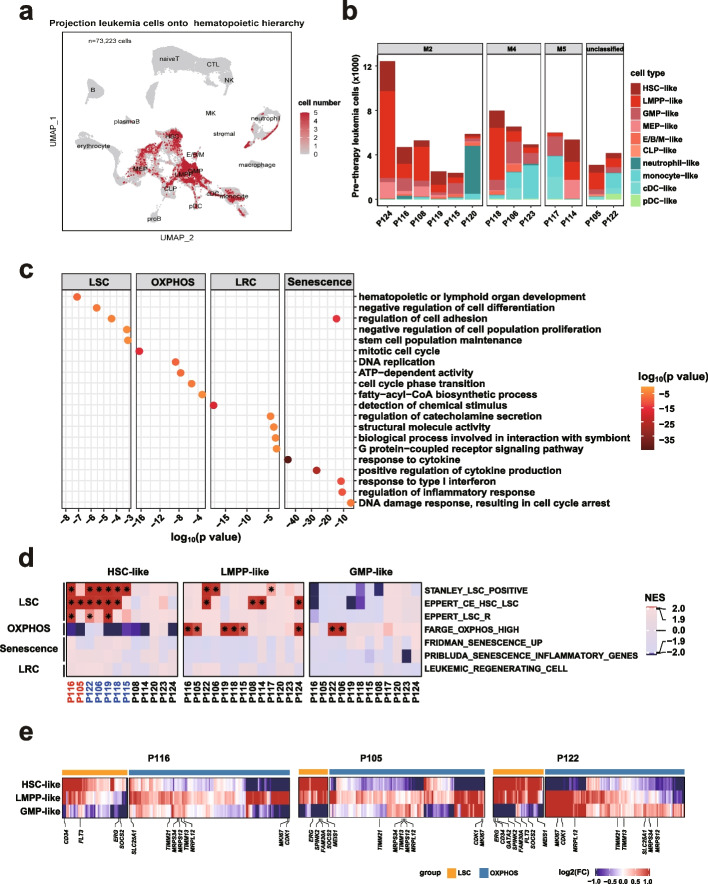
LSC and OXPHOS signatures were prevalent in leukemic stem and progenitor populations. **a** UMAP plot illustrating the projection of transcriptionally predicted leukemic cells from thirteen AML patients onto the normal hematopoietic hierarchy, based on transcriptomic similarity to normal cells. Projected cells are highlighted, with shading indicating the frequency of being projected. **b** Bar plot showing the cell counts of pre-therapy leukemia cell populations in each AML patient. **c** Dot plot displaying pathways enriched by four known chemoresistance-related expression signatures derived from mouse model studies, with colors representing enrichment *p* values. **d** Heatmap depicting the GSEA results of the four expression signatures in panel c for each HSPC-like population compared to all other leukemic populations within each patient before therapy. Colors represent NES values obtained from GSEA analysis, and an asterisk denotes both NES > 1.9 and FDR < 0.001. Patient code colors indicate resistant (red) and sensitive (blue) cases. **e** Heatmaps showing expression fold changes (FC) of core enriched genes (columns) contributing to LSC and OXPHOS signatures in each HSPC-like population (rows) compared to all other leukemic populations in three representative patients (P116, P105, and P122). Core enriched genes are identified from GSEA results and those related to cell stemness and metabolism are indicated

Leukemic cells from AML patients were found to reside in different cellular hierarchies [22, 25]. To identify leukemic cells with chemoresistant potential at the time of diagnosis, we first annotated the cellular types of each of the AML cells. Specifically, each single cell was projected to the nearest healthy counterpart based on the cosine similarity calculated from the expression of cell type-specific genes using the scmap tool (see “Material and methods”) [26], which showed general agreement with previous classifications in annotating cell types across multiple public single-cell datasets (Additional file 1: Fig. S3a, b). The tumor cells resembled one of the ten normal cell types along the HSC to myeloid axis with a high median cosine similarity of 0.85 (range: 0.35–0.91; Additional file 1: Fig. S3c-d), and were named their healthy counterpart with a “-like” suffix (Fig. 3a). Consistent with recent single cell studies [22], the composition of different cell types varied between patients and generally agreed with the clinical French–American–British (FAB) classification, except for in three patients (P114, P118, and P120; Fig. 3b). To clarify this, we assessed the proportion of HSPC-like cells using flow cytometry by examining the expression of canonical stem cell markers (CD34 and CD117). Flow cytometry supported the scRNA-seq prediction and showed a high proportion (77.40% and 95.14%) of HSPC-like populations in two patients (P114 and P118, Additional file 1: Fig. S3e).

We then investigated the chemoresistant potential of each tumor population based on the presence of known chemoresistance-related gene expression signatures. Four transcription signatures were identified to be associated with chemoresistance in PDX models, including LSC activity, active OXPHOS, LRC, and senescence (Additional file 5: Table S4) [6–8, 27, 28]. These molecular signatures represented distinct biological functions with little overlap in the associated genes (Fig. 3c). Only leukemic cell populations with sufficient cell numbers were used for the following analysis. Among the 73 cell populations derived from thirteen patients, these features were significantly enriched in the populations that resembled HSPCs, including HSC, LMPP, and GMP (20/37 vs, 0/36, *p* < 0.0001; Fig. 3d and Additional file 1: Fig. S4a). Interestingly, the presence of LSC and OXPHOS signatures was mutually exclusive in different populations. Cell populations with the LSC signature were either HSC-like (7/12, 58.33%) or LMPP-like (6/13, 46.16%), while the OXPHOS signature was mainly restricted to a different subset of LMPP-like and GMP-like cell populations. LRC and senescence signatures were largely undetectable. In addition, LMPP-like cells showing different chemoresistant signatures were from different AML patients. Specifically, LMPP-like cells within AML-M4/M5 patients tended to highly express the OXPHOS signature, while those from AML-M2 patients were likely to show LSC signatures (Additional file 4: Table S3). We also examined the expression of the core enriched genes that contributed to each signature. An analogous pattern of mutually exclusive expression of the core enriched genes was observed in HSPC-like populations (Fig. 3e and Additional file 1: Fig. S4b). HSC- and/or LMPP-like populations with LSC signatures highly expressed several well-known genes related to stemness (e.g., *CD34* and *ERG*). In contrast, LMPP-like and GMP-like subpopulations with the OXPHOS signature exhibited higher expression of metabolic genes (e.g., *SLC25A1* and *MRPS34*).

To explore whether the two major chemoresistance features were exclusively present in HSPC-like populations in independent cohorts, we reanalyzed recently published scRNA-seq data from eleven adult AML samples [21]. Consistent with our findings, 62.5% (five out of eight) of populations with LSC signatures were HSC-like, while higher OXPHOS expression signatures were present in LMPP-like or GMP-like populations (Additional file 1: Fig. S4c). The loss of self-renewal capacity and increase in OXPHOS also occurred during normal myeloid development (Fig. 1d), suggesting that these are conserved biological features in both normal and malignant conditions. Together, these findings suggest that LSC and OXPHOS, two known chemoresistance-related signatures derived from mouse models, are present in different HSPC-like populations.

To explore whether the populations containing LSC or OXPHOS signatures were enriched for chemoresistant cells, we first examined the changes in AML cell composition over the course of chemotherapy. Interestingly, ten patients who achieved complete remission (CR) displayed a decrease in cellular diversity in response to treatment, with a significant reduction in the variety of early stem and progenitor populations (Additional file 4: Table S3; Additional file 1: Fig. S4e). In contrast, two (P116 and P105) out of three patients who achieved partial remission (PR) maintained diverse cell types (Additional file 4: Table S3; Additional file 1: Fig. S4e). Furthermore, the presence of chemoresistance-related signatures was correlated with treatment response. All seven diagnostic HSPC-like populations without LSC or OXPHOS signatures were cleared after chemotherapy (Fig. 3d and Additional file 1: Fig. S4e). In contrast, two (P116 and P105) out of seven patients whose pre-therapy HSC-like cell populations carried LSC signatures had an average of 147 cells (1.76% of total cells) that survived after chemotherapy, and half (three out of six; P116, P105, and P124) of patients whose diagnostic LMPP-like populations carried an active OXPHOS signature had 0.17%-2.57% of total cells persisting at remission (Fig. 3d and Additional file 1: Fig. S2e). Importantly, the persistence of AML cells after chemotherapy in these patients was also supported by morphology examination and flow cytometry data (Additional file 4: Table S3).

**Fig. 4:**
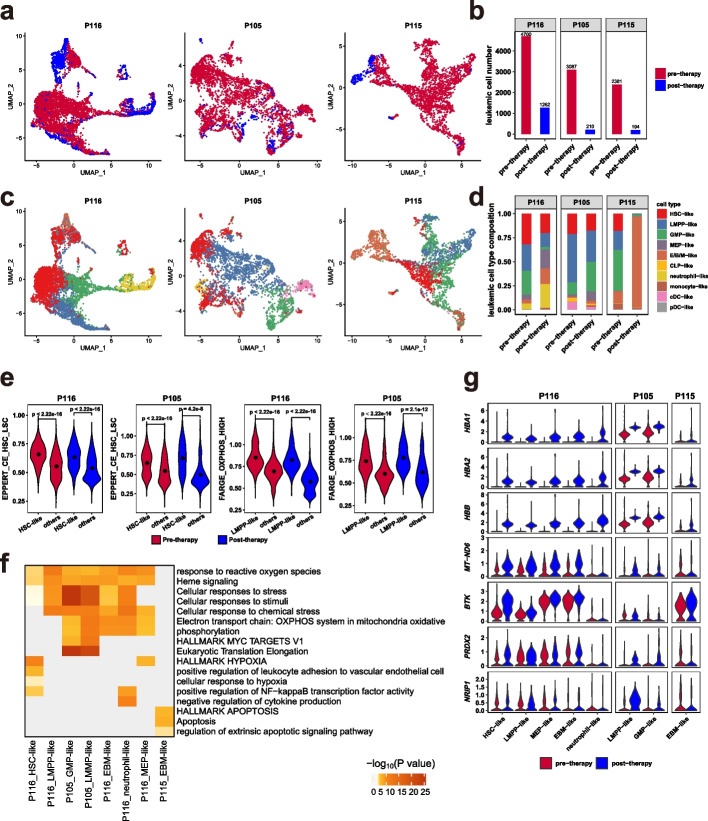
Dynamic cellular and transcriptomic changes in leukemic cells after chemotherapy in P116, P105, and P115. **a**,**c** UMAP plots of leukemic cells from pre- and post-therapy samples for each patient, with cells color-coded by sample origin (**a**) and cell type (**c**). **b** Bar plot showing the number of leukemic cells in samples described in **a**. **d** Bar plot depicting the distribution of cell types in samples described in **c**. **e** Violin plots of normalized single-cell expression scores for LSC and OXPHOS signatures in HSC-like and LMPP-like cells from patients P116 and P105, with black dots representing average signature expression. *P* values were calculated using the Wilcoxon signed-rank test. **f** Heatmap visualization of Metascape pathways enriched by upregulated genes in each cell population from post-therapy samples compared to pre-therapy samples. **g** Violin plots showing the expression of genes related to heme metabolism signaling pathways and response to reactive oxygen species in pre- and post-therapy cell populations

Next, we investigated the transcriptional features of AML cells that survived chemotherapy. Three patients (P105, P115, and P116) who had hundreds of cells (average: 492; range: 194–1,262) remaining after treatment were used for this analysis (Fig. 4a-d). We performed high-dimensional clustering analysis and UMAP projection of pre- and post-therapy leukemic cells from each patient (Fig. 4a, b). This analysis revealed that most post-therapy cells overlapped with pre-therapy leukemic cells in their distribution, while some cells showed transcriptional changes that shifted their location within the projection (Fig. 4a). Single-cell gene signature score analysis showed that post-therapy HSC- and LMPP-like cells maintained high expression of LSC and OXPHOS signatures, respectively (Fig. 4e). Gene enrichment analysis of the upregulated genes confirmed these results (Fig. 4f). Specifically, self-renewal-associated signaling pathways (e.g., hypoxia and NF-κB) were highly expressed in post-therapy HSC-like populations, while biological processes related to oxidative phosphorylation were activated in progenitor-like cells (Fig. 4f). E/B/M-like cells in P115 exhibited an increased transcriptional activity in the apoptosis pathway after therapy (Fig. 4f), which was consistent with prior in vitro and in vivo studies showing that cytarabine induces DNA double strand breaks and apoptotic morphology [29]. Notably, post-therapy AML cells from patients who achieved partial remission (P116 and P105) displayed activation of response to reactive oxygen species (*PRDX2*, *BTK, NRIP1*) and heme metabolism signaling pathways (*HBB*, *HBA1*, *HBA2*) compared to the pre-therapy populations (Fig. 4f, g). Together, these results indicate that chemo-surviving HSPC-like cells acquire enhanced metabolic features while maintaining the original LSC and OXPHOS signatures.

### Identification of a chemoresistant HSC-like subpopulation characterized by the surface marker *CD69*

**Fig. 5:**
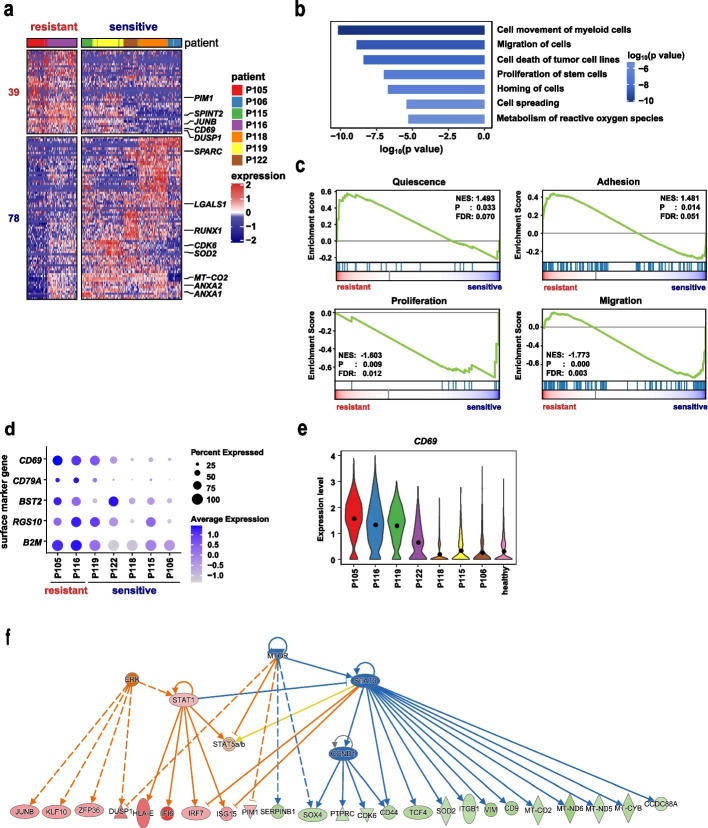
Characterization of the *CD69*^+^ HSC-like cell subpopulation potentially conferring chemoresistance. **a** Heatmap displaying differentially expressed genes (DEGs) in pre-therapy HSC-like populations between resistant cases (P105 and P116) and sensitive cases (P115, P106, P118, P119, and P120). **b** Bar plot presenting representative suppressed biological functions enriched by DEGs in panel **a** using Ingenuity Pathway Analysis (IPA). **c** GSEA plots showing the enrichment of quiescence, proliferation, adhesion (KEGG term: cell adhesion molecules), and migration (KEGG term: leukocyte transendothelial migration) signatures in HSC-like cells from resistant cases compared to sensitive cases. **d** Dot plots of normalized expression of differentially expressed surface marker genes between resistant and sensitive cases. Dot size represents the proportion of cells expressing a gene in a patient’s HSC-like cell population, and shading indicates the relative expression level. **e** Violin plots depicting *CD69* expression in HSC-like populations from patients and HSC populations from healthy donors. **f** Regulatory network showing upstream regulators and their targets predicted to be activated or suppressed in HSC-like cells from resistant cases. Colors indicate increased (red) or decreased (green) gene expression relative to sensitive cases. Red and blue lines represent known activating or inhibitory effects, respectively, between each regulator and its targets

We further focused on the seven patients (out of thirteen; P116, P105, P122, P106, P119, P118, and P115) whose HSC-like populations possessed LSC signatures. The HSC-like populations persisted after therapy in two (P116 and P105) of the seven patients (Figs. 3d and 4d). Therefore, we referred to the two patients with persistent HSC-like populations as “resistant cases”. In contrast, HSC-like populations in the remaining five patients became undetectable after therapy, and we referred to these patients as “sensitive cases” (Additional file 1: Fig. S4e). To explore the molecular features underlying the differential therapy response of HSC-like populations, we compared pre-therapy HSC-like populations from resistant and sensitive cases. We found 117 differentially expressed genes (DEGs) that were unique to patients with a resistance phenotype (Fig. 5a and Additional file 6: Table S5). Ingenuity Pathway Analysis (IPA) biological function and upstream regulator analysis revealed that those genes were related to the repression of proliferation of stem cells (e.g., *CDK6, CCND1, JUNB, SPARC*)*,* cellular movement (e.g., *CD69, DUSP1, LGALS1, ANXA1*)*,* hematopoietic differentiation regulators (e.g., *GATA1, CEBPA, RUNX1, ZFP36*) and activation of glucose metabolism (e.g., *SOD2, MT-CO2*; Fig. 5b, Additional file 1: Fig. S5a, and Additional file 7: Table S6)*.* Consistently, GSEA showed that the HSC-like populations from resistant cases were enriched for specific gene expression signatures derived from hematopoietic cells, including HSC self-renewal capacity, leukemia quiescent state, and leukocyte adhesion (Fig. 5c). Although these biological processes were similarly present in the LSC expression signature, this analysis suggests that the HSC-like populations from resistant cases may have enhanced functions.

Among several differentially expressed cell surface marker genes (*CD69*, *CD79A*, *CD317/BST2*, *RGS10*, and *B2M*), *CD69,* a type II transmembrane C-type lectin receptor, exhibited the most prominent difference between resistant and sensitive HSC-like cells (fold change = 1.75; Fig. 5d and Additional file 6: Table S5). Furthermore, in the two resistant patients, nearly 90% of HSC-like cells expressed *CD69* (90.00% in P105 and 89.20% in P116), while less than 40% of HSC-like cells (median: 39.52%, range: 24.40%-88.97%) from the sensitive patients did (Fig. 5d-e). These data suggested that HSC-like populations that were able to survive chemotherapy were dominated by *CD69*^+^ cells (named the *CD69*^+^ HSC-like subpopulation), while those that became undetectable after chemotherapy were enriched for *CD69*^−^ HSC-like cells (named the *CD69*^−^ HSC-like subpopulation, Fig. 5d-e). In addition, the UMAP projection of HSC-like populations from these seven patients showed two major clusters (Additional file 1: Fig. S4f). Resistant HSC-like subpopulations were clustered together and showed significantly higher expression of *CD69*, while the majority (four out of five) of sensitive HSC-like subpopulations formed another cluster with lower expression of *CD69*. The expression of *CD69* was still maintained in post-therapy HSC-like subpopulations of the two resistant patients (Additional file 1: Fig. S5b). In addition, the mRNA and surface protein levels of CD69 showed a strong correlation in primary AML samples (R = 0.89, *p* = 0.045; Additional file 1: Fig. S5c), and the expression of *CD69* was minimally detected in HSCs from healthy donors (Fig. 5e, Additional file 1: Fig. S5d). These findings suggest that *CD69* can serve as a potential biomarker for chemoresistant HSC-like subpopulations.

Considering that CD34^+^CD38^−^ leukemic cells immunophenotypically resemble HSCs and functionally enrich LSCs, we investigated whether the *CD69*^+^CD34^+^CD38^−^ population could recapitulate the expression signature in a single-cell analysis-defined *CD69*^+^ HSC-like subpopulation. We utilized publicly available bulk microarray expression profiles of flow cytometry-sorted CD34^+^CD38^−^ cells. We divided 54 samples from 78 AML patients into the *CD69*^+^CD34^+^CD38^−^ group and the *CD69*^−^CD34^+^CD38^−^ group based on the expression level of *CD69* (See “Material and methods”; Additional file 1: Fig. S5e) [30]. The differential gene expression analysis between these two groups (named “bulkRNA DEGs”) revealed a similar set of biological function terms with our scRNA DEGs (Additional file 1: Fig. S5f-g). Specifically, bulkRNA DEGs of *CD69*^+^CD34^+^CD38^−^ cells were associated with the activation of adhesion (*CXCR4*, *DUSP1, CXCL2, CCL3/5, CCL3L1/3*), viability (*MCL1*, *LYZ*), cell cycle repression (*SPARC*, *CDKN1A, BTG1/2*), and suppression of differentiation (*RUNX1*, *ZFP36*), as revealed by IPA biological function analysis (Additional file 1: Fig. S5f,h and Additional file 8: Table S7). In agreement with these findings, the known signatures relevant to leukemia quiescence and adhesion to vascular endothelial cells were enriched in *CD69*^+^CD34^+^CD38^−^ populations (Additional file 1: Fig. S5i). Therefore, this dataset supported the notion that the *CD69*^+^CD34^+^CD38^−^ combination serves as a surrogate for enriching the *CD69*^+^ HSC-like subpopulation.

**Fig. 6:**
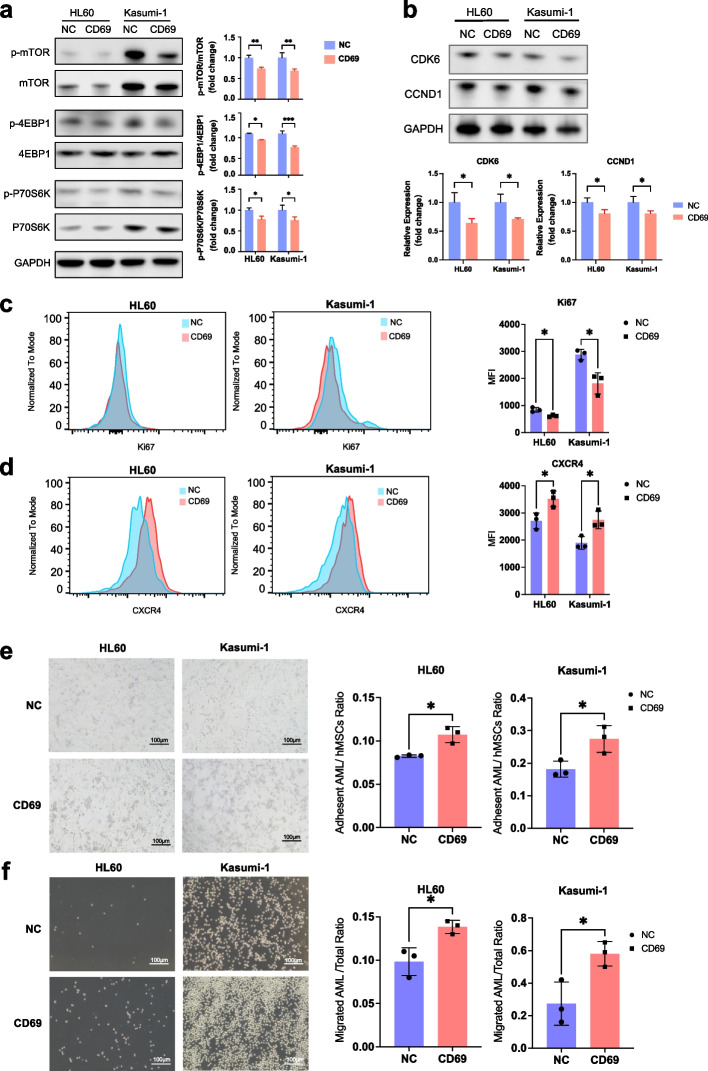
*CD69* overexpression inhibits the mTOR pathway and enhances AML cell adhesion and migration. **a** (Left) Western blot showing total and phosphorylated protein levels of mTOR, 4EBP1, and P70S6K in negative control (NC) and *CD69*-overexpressing HL60 and Kasumi-1 cells. (Right) Bar plots displaying relative quantification by densitometry. **b** (Top) Western blot showing total protein levels of CDK6 and CCND1 in NC and *CD69*-overexpressing HL60 and Kasumi-1 cells. (Bottom) Bar plots displaying relative quantification by densitometry. **c** Representative histograms (left) and corresponding statistical results (right) of flow cytometry analyses showing protein levels of the classic proliferation marker Ki67 in NC and *CD69*-overexpressing HL60 and Kasumi-1 cells. **d** Representative histograms (left) and corresponding statistical results (right) of flow cytometry analyses showing protein levels of surface chemokine receptor CXCR4 on NC and *CD69*-overexpressing HL60 and Kasumi-1 cells. **e** Representative images (left) and corresponding statistical results (right) showing adhesion capacity of NC and *CD69*-overexpressing HL60 and Kasumi-1 cells to hMSCs. **f** Representative images (left) and corresponding statistical results (right) showing migration of NC and *CD69*-overexpressing HL60 and Kasumi-1 cells toward CXCL12 and S1P respectively. * *p* < 0.05; **p < 0.01; ***p < 0.001; ns, not significant; t test. Mean ± SEM values are shown for panels a, c-f

In addition, we were particularly interested in investigating the regulatory network to provide mechanistic information related to drug resistance. Upstream regulator analysis identified MTOR and STAT3 as two major suppressed hubs, which were associated with decreased expression of cell cycle regulators (e.g., *CDK6* and *CCND1*) as well as upregulation of CXCR4-mediated microenvironmental interaction molecules (e.g., *PIM1*) in *CD69*^+^ HSC-like subpopulations (Fig. 5f). As the expression level of CD69 in AML cell lines was either very low or undetectable, *CD69*-overexpressing AML cell lines were established (Additional file 1: Fig. S6a), to address the functional role of CD69 in regulating its downstream pathways. We found that *CD69* overexpression resulted in reduced phosphorylated protein levels of mTOR and its key downstream effectors (P70S6K and 4EBP1), as well as decreased total protein levels of mTOR and P70S6K in both HL60 and Kasumi-1 cells (Fig. 6a). The relative levels of phosphorylation of mTOR, P70S6K and 4EBP1, shown as the fold change in the levels of phosphorylated protein over total protein levels, were significantly lower in *CD69*-overexpressing cell lines than those in controls. The total and phosphorylated protein levels of STAT3 were comparable in control and *CD69*-overexpressing HL60 or Kasumi-1 cells, respectively (Additional file 1: Fig. S6b). Moreover, *CD69* overexpression decreased the expression of the classic proliferation marker Ki67 and the regulators CCND1 and CDK6, and increased the adhesion molecule CXCR4 expression (Fig. 6b-d). Subsequently, we analyzed the adhesive interaction of these cell lines with human mesenchymal stem cells (hMSCs). Cell adhesion assays showed that *CD69* overexpression significantly increased the ratio of adherent cells to hMSCs (Fig. 6e). Since homing to bone marrow is a crucial step for AML cells to interact with stromal cells, we used a Transwell assay to determine if CD69 affects AML cell migration to CXCL12, which is expressed in BM niches. *CD69* overexpression increased cell migration toward a high gradient of CXCL12 (Fig. 6f). These data suggested that CD69 enhanced cell adhesion and homing to the BM niche through the CXCR4-CXCL12 interaction. In concordance with our findings in AML cell lines, the protein levels of Ki67 were significantly reduced and the protein levels of CXCR4 were increased in CD69^high^CD34^+^CD38^−^ populations from primary AML patients (Additional file 1: Fig. S7a-c).

Collectively, these findings suggest that *CD69*^+^ HSC-like cells possess enhanced abilities to adhere to the microenvironment and maintain cellular quiescence via dysregulated mTOR signaling, potentially contributing to their resistance to chemotherapy.

### The *CD69*^+^ HSC-like cell subpopulation was associated with adverse clinical outcomes

To explore the clinical relevance of the *CD69*^+^ HSC-like subpopulation, we utilized two large public cohorts, TARGET and TCGA, containing mRNA expression data from pediatric and adult AMLs, respectively. We employed the EPIC deconvolution, a widely used quantification algorithm [31], to infer the cellular composition of a mixed population from bulk gene expression data. As EPIC utilizes cell type-specific mRNA expression for the inference of subpopulation abundance, we first compiled a list of expression signatures for 11 commonly observed leukemic cell types (Additional file 9: Table S8; Additional file 1: Fig. S8a). To test the performance of EPIC in the deconvolution of leukemia populations, we carried out a simulation analysis on artificial bulk data of 2,529 samples with known cell identity derived from our scRNA-seq profiles (see “Material and methods”). This analysis showed that EPIC predicted the abundances of all 11 leukemic cell types with high agreement with their known proportions (*R* = 0.95–0.99, *p* < 10^–10^) (Additional file 1: Fig. S8b).

**Fig. 7:**
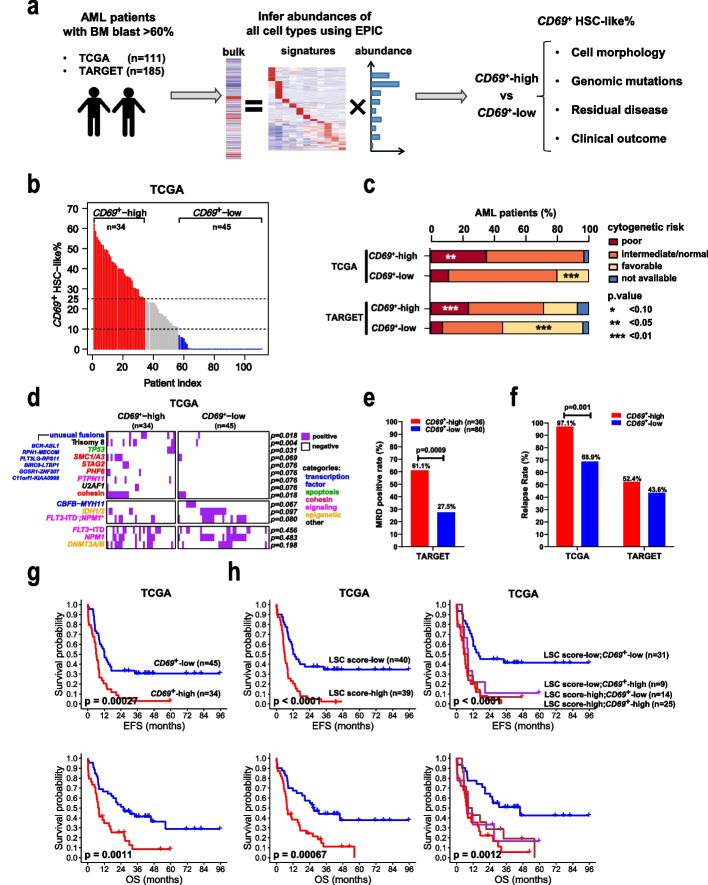
Clinical and genomic features of AML patients with different *CD69*^+^ HSC-like cell proportions. **a** Schematic workflow illustrating the exploration of the clinical relevance of the *CD69*^+^ HSC-like subpopulation in two large public AML cohorts. **b** Estimated proportions of *CD69*^+^ HSC-like subpopulation (*CD69*^+^ HSC-like%) in pre-therapy samples from TCGA-AML patients. Patients were grouped into *CD69*^+^-high (red), *CD69*^+^-middle (grey), and *CD69*^+^-low (blue) according to *CD69*^+^ HSC-like%, with dashed black lines indicating the cutoffs. **c** Histogram showing the percentages of patients with different cytogenetics-based prognostic risk categories in each group. **d** Heatmap showing the presence of genomic alterations in samples from TCGA-AML patients. Genomic alterations (rows) are colored according to the biological functions of their corresponding genes. The cohesin term includes mutations of the core complex subunits *STAG2*, *RAD21*, *SMC1A/3/5*, or the modulator *PDS5B*. *FLT3*-ITD^−^;*NPM1*^+^ represents mutated *NPM1 without FLT3*-internal tandem duplication (ITD). Unusual fusions are indicated. **e** Flow cytometry-based measurable residual disease (MRD) positive rates in the TARGET-AML patients at the end of the first cycle of chemotherapy regimen. A patient was defined as MRD-positive if the MRD level was equal to or greater than 0.1%. **f** Relapse rates in two groups of AML patients from each cohort. **g** Kaplan–Meier curves showing the event-free survival (EFS) and overall survival (OS) of TCGA-AML patients stratified by *CD69*^+^ HSC-like%. **h** Kaplan–Meier curves showing the survivals of TCGA-AML patients stratified by LSC score alone or combined with *CD69*^+^ HSC-like%. All *p* values in panels **c**, **d**, **e**, and **f** were calculated using Fisher’s test. All *p* values in panels g and h were calculated using the log-rank test

We further applied this algorithm to estimate the abundances of various leukemic cell types in AML patients from bulk mRNA-seq data of TARGET (*n* = 185) and TCGA (*n* = 111) (Fig. 7a). The inferred cell type compositions were generally consistent with the morphology-based FAB classification (Additional file 1: Fig. S8c and Additional file 9: Table S8). The majority (75–80%) of AML-M0 patients were estimated to contain more than 30% HSC/LMPP-like cells with few mature myeloid cell types (Additional file 1: Fig. S8c). This result was consistent with the notion that the M0 subtype is characterized by a high proportion (> 30%) of undifferentiated blasts [32]. AML-M3 is known to be characterized by the accumulation of immature promyelocytes that account for at least 30% of marrow cells [33]. In accordance with this, nearly all (9/10) AML-M3 patients were dominated by GMP-like cells resembling promyelocytes (Additional file 1: Fig. S8c). In addition, more than 75% of patients with EPIC-inferred monocyte components at least 5% (84.5% and 75.0% for TARGET and TCGA, respectively) fell into the M4/M5 subtypes (Additional file 1: Fig. S8c). This observation was consistent with the morphological characteristics of AML-M4/M5 that are enriched for monoblasts, promonocytes, and monocytes. Furthermore, AML patients with higher expression of the HSC/progenitor-like signature were reported to have significantly shortened overall survival (OS) and event-free survival (EFS) than those with higher expression of the GMP-like signature in a single-cell transcriptomic study [22]. Consistently, patients with more HSC/LMPP-like cells inferred by our analysis had significantly worse outcomes than those with more GMP-like cells (*p* < 0.05, Additional file 1: Fig. S8d, e). Altogether, these data showed that cellular fraction inference by EPIC could largely recapitulate the hierarchical composition of AML patients.

Notably, the estimated proportions of *CD69*^+^ HSC-like cells ranged from 0 to 65% (TARGET mean = 13.3%; TCGA mean = 16.3%; Fig. 6b, Additional file 1: Fig. S9a, and Additional file 9: Table S8). Based on the abundance of this subpopulation, we separated patients into *CD69*^+^-high (> 25% of total cells are *CD69*^+^ HSC-like) and *CD69*^+^-low (< 10% of total cells are *CD69*^+^ HSC-like) groups (TARGET *n* = 42 and 110, respectively; TCGA *n* = 34 and 45, respectively). Approximately 23–30% and 40–60% of patients were *CD69*^+^-high and *CD69*^+^-low, respectively (Fig. 7b and Additional file 1: Fig. S9a). Differential expression and IPA function analyses showed that *CD69*^+^-high patients from the two cohorts consistently exhibited key transcription features revealed by single-cell analysis of *CD69*^+^ HSC-like cells, including significant repression of cellular movement and migration (e.g., *SPINT2*, *S100A8/9*, *CSF3R*, *TLR2*, *CXCL16*), hematopoietic differentiation (e.g., *CEBPA*, *SPI1*, *ZFP36*) and cell death, as well as activation of self-renewal (e.g., *ERG*, *GATA2*) and cell survival (e.g., *HOPX*, *BMI1*) (Additional file 1: Fig. S9b-c, Additional file 10: Table S9, and Additional file 11: Table S10).

We next examined whether AML patients with different *CD69*^+^ HSC-like cell proportions would be associated with specific known AML characteristics. The *CD69*^+^-high group had more patients with AML-M0 subtypes (TARGET, 9.5% vs. 2.7%, *p* = 0.092; TCGA, 23.5% vs. 2.2%, *p* = 0.004), while the *CD69*^+^-low group was enriched for AML-M2/M4 subtypes (TARGET, 56.4% vs. 19.1%, *p* = 0.000; TCGA, 44.5% vs. 23.6%, *p* = 0.062, Table 1). Interestingly, patients from the *CD69*^+^-high group corresponded closely to genetic alterations that are associated with poor prognosis, such as *FLT3*-internal tandem duplication (ITD) with high allelic ratios in the TARGET cohort (19.0% vs. 5.5%) and *TP53* mutations in the TCGA cohort (11.8% vs. 0.0%) (Fig. 7c, d, and Additional file 1: Fig. S9d). AML patients harboring trisomy 8, *PTPN11* alterations, and/or unusual fusions largely fell into the *CD69*^+^-high group. In contrast, *CD69*^+^-low patients significantly overlapped with patients carrying known favorable mutations such as *CBFB*-*MYH11* fusions and *NPM1* mutations without *FLT3*-ITD (Fig. 6d and Additional file 1: Fig. S8d). Notably, in our scRNA-seq cohort, the *PTPN11* mutation was only present in the resistant patient (P105), while *CBFB-MYH11* fusions exclusively occurred in the sensitive patients (Additional file 1: Fig. S9d).

**Table 1:** The numbers and relative frequencies of the different AML subtypes within each group of patients

**Cohort**	**TARGET**			**TCGA**			
**Group**	**High (*n* = 42)**	**Low (*n* = 110)**	***p*.value**	**High (*n* = 34)**	**Low (*n* = 45)**	***p*.value**	
**FAB subtype**							
**M0**	**9.5**	**2.7**	0.092^*^	**23.5**	**2.2**	0.004^***^	
**M1**	**26.2**	**15.5**	**0.160**	**32.4**	**37.8**	**0.481**	
**M2**	**4.8**	**26.4**	0.003^***^	**8.8**	**15.6**	**0.749**	
**M4**	**14.3**	**30.0**	0.061^*^	**11.8**	**28.9**	0.097^*^	***p*.value**
**M5**	**19.0**	**19.1**	**1**	**14.7**	**15.6**	**1**	^*^ < 0.10
**M6**	**0**	**0**	**NA**	**2.9**	**0**	**0.430**	^**^ < 0.05
**M7**	**2.4**	**0**	**0.276**	**0**	**0**	**NA**	^***^ < 0.01
**NOS**	**14.3**	**0.9**	0.002^***^	**0**	**0**	**NA**	
**unknown**	**9.5**	**5.5**	**0.464**	**5.9**	**0**	**0.182**	

Fisher*-*exact* P* values were calculated when compared two groups within each cohort

To investigate whether the *CD69*^+^-high group of patients had worse clinical outcomes, we first analyzed the TARGET cohort, which contains clinical measurable residual disease (MRD) data determined by flow cytometry analysis. At the end of the first cycle of the chemotherapy regimen, 37.9% (44/116) of patients were found to be MRD-positive, with a clinical cutoff of 0.1%. Notably, the MRD positivity rate for the *CD69*^+^-high group was 61.1% (22/36), which was significantly higher than that of the *CD69*^+^-low group, irrespective of the therapeutic regimen used (22/80, 27.5%, *p* = 0.0009; Fig. 7e and Additional file 1: Fig. S9e). Additionally, the *CD69*^+^-high group exhibited an increased relapse rate and worse EFS and OS than the *CD69*^+^-low group, regardless of whether they had undergone transplantation (Fig. 7f, g and Additional file 1: Fig. S9f). Our univariate and multivariate analyses identified the abundance of *CD69*^+^ HSC-like cells, in addition to the LSC score and MRD status, as independent factors for poor EFS and OS (Tables 2 and 3). Furthermore, the *CD69*^+^ HSC-like proportion enabled further stratification of patients in the LSC-defined low-risk group of adult patients and the high-risk group of pediatric patients, respectively (Fig. 6h and Additional file 1: Fig. S9g).

**Table 2:** Univariate and multivariate analyses for clinical characteristics of EFS and OS in pediatric AML patients

TARGET cohort (*n* = 152)	Univariate				Multivariate			
*Variable*	EFS HR (95% CI)	EFS *p*.value	OS HR (95% CI)	OS *p*.value	EFS HR (95% CI)	EFS *p*.value	OS HR (95% CI)	OS *p*.value
*Age* > *10*	1.24 (0.826–1.86)	0.299	1.41 (0.853–2.34)	0.179				
*BM blast* > *70%*	1.36 (0.822–2.25)	0.231	1.25 (0.675–2.3)	0.482				
***CD69***^**+**^ ***HSC-like%*** ***(high*** ***vs*** ***low)***	**1.51 (0.984–2.33)**	0.059^*^	**2.02 (1.22–3.36)**	0.007^***^	**1.05 (0.565–1.95)**	**0.880**	**1.96 (0.891–4.3)**	0.095^*^
***LSC score (high vs low)***	**2.02 (1.34–3.06)**	0.001^***^	**1.97 (1.17–3.3)**	0.010^**^	**2.39 (1.35–4.23)**	0.003^***^	**2.12 (0.993–4.53)**	0.052^*^
*WBC* > *30*	1.20 (0.781–1.86)	0.400	1.08 (0.632–1.83)	0.787				
*Gender (male vs female)*	0.894 (0.596–1.34)	0.589	0.85 (0.516–1.4)	0.522				
*Cytogenetic inv(16)*	0.811 (0.441–1.49)	0.502	0.481 (0.192–1.2)	0.118				
*Cytogenetic MLL*	1.34 (0.804–2.22)	0.263	1.26 (0.667–2.38)	0.475				
*Cytogenetic Normal*	0.661 (0.408–1.07)	0.093^*^	0.766 (0.425–1.38)	0.377				
*Cytogenetic Other*	**2.80 (1.75–4.47)**	0.000^***^	**2.45 (1.4–4.28)**	0.002^***^				
*Cytogenetic t(8;21)*	**0.495 (0.256–0.957)**	0.037^*^	0.629 (0.285–1.39)	0.251				
*Risk group High*	1.37 (0.746–2.52)	0.310	1.24 (0.561–2.73)	0.597				
***Risk group Standard***	**2.55 (1.67–3.89)**	0.000^***^	**2.36 (1.39–4.01)**	0.001^***^	**1.34 (0.5–3.6)**	**0.560**	**1.30 (0.397–4.23)**	**0.667**
***Risk group Low***	**0.337 (0.214–0.531)**	0.000^***^	**0.369 (0.207–0.658)**	0.001^***^	**0.694 (0.244–1.98)**	**0.494**	**0.681 (0.181–2.56)**	**0.570**
*FLT3-ITD*	**1.63 (0.972–2.72)**	0.064^*^	1.23 (0.622–2.41)	0.556				
*FLT3-ITD AR high*	1.53 (0.795–2.95)	0.203	1.28 (0.552–2.98)	0.564				
*FLT3-ITD AR low*	1.60 (0.774–3.3)	0.205	1.11 (0.403–3.06)	0.840				
*FLT3-PM*	1.31 (0.68–2.53)	0.418	0.794 (0.318–1.98)	0.620				
*CEBPA mutation*	**0.259 (0.0819–0.818)**	0.021^**^	0.481 (0.151–1.54)	0.217				
*NPM1 mutation*	**0.30 (0.11–0.819)**	0.019^**^	**0.255 (0.0622–1.04)**	0.057^*^				
*RUNX1 mutation*	0.963 (0.237–3.91)	0.958	1.51 (0.37–6.21)	0.564				
*WT1 mutation*	1.21 (0.646–2.27)	0.549	1.16 (0.55–2.43)	0.702				
*Anti-CD33 treatment*	0.891 (0.564–1.41)	0.620	1.23 (0.679–2.23)	0.495				
***MRD at end of course 1***	**2.49 (1.56–3.97)**	0.000^***^	**2.38 (1.31–4.3)**	0.004^***^	**1.71 (0.956–3.05)**	0.071^*^	**1.51 (0.7–3.26)**	**0.293**
*MRD at end of course 2*	**3.41 (1.93–6.01)**	0.000^***^	1.69 (0.8–3.58)	0.168				
***SCT in first CR***	**0.326 (0.132–0.808)**	0.016^**^	**0.314 (0.0979–1.01)**	0.052^*^	**0.366 (0.136–0.983)**	0.046^**^	**0.367 (0.103–1.3)**	**0.121**

Factors with *P* < 0.10 in the univariate analyses were subjected to multivariate analysis. HR and CI represent hazard ratio and confidence interval respectively

^*^p < 0.10

^**^p < 0.05

^***^p < 0.01

**Table 3:** Univariate and multivariate analyses for clinical characteristics of EFS and OS in adult AML patients

TCGA cohort (*n* = 79)	Univariate				Multivariate			
*Variable*	EFS HR (95% CI)	EFS *p*.value	OS HR (95% CI)	OS *p*.value	EFS HR (95% CI)	EFS *p*.value	OS HR (95% CI)	OS *p*.value
***Age* > *60***	**1.93 (1.18–3.17)**	0.009^***^	**2.24 (1.32–3.81)**	0.003^***^	**1.45 (0.846–2.47)**	**0.178**	**1.58 (0.883–2.82)**	**0.124**
*BM blast* > *70%*	1.54 (0.804–2.96)	0.192	1.48 (0.747–2.94)	0.260				
***CD69***^**+**^ ***HSC-like% (high*** ***vs*** ***low)***	**2.47 (1.5–4.07)**	0.000^***^	**2.36 (1.39–4.02)**	0.001^***^	**1.85 (1.04–3.27)**	0.035^**^	**2.1 (1.17–3.76)**	0.013^**^
***LSC score (high*** ***vs*** ***low)***	**2.87 (1.72–4.81)**	0.000^***^	**2.47 (1.44–4.23)**	0.001^***^	**2.5 (1.34–4.66)**	0.004^***^	**2.11 (1.12–3.98)**	0.021^**^
*Gender (male vs female)*	0.988 (0.604–1.62)	0.962	0.943 (0.56–1.59)	0.824				
*Cytogenetic Risk Favorable*	0.606 (0.261–1.41)	0.243	0.514 (0.205–1.29)	0.157				
*Cytogenetic Risk Intermediate/Normal*	1.1 (0.648–1.88)	0.719	1.22 (0.689–2.16)	0.495				
*Cytogenetic Risk Poor*	1.21 (0.668–2.2)	0.529	1.18 (0.624–2.25)	0.606				
*Molecular Risk Good*	0.606 (0.261–1.41)	0.243	0.514 (0.205–1.29)	0.157				
*Molecular Risk Intermediate*	0.958 (0.575–1.6)	0.870	0.833 (0.486–1.43)	0.504				
***Molecular Risk Poor***	**1.39 (0.806–2.41)**	**0.234**	**1.87 (1.05–3.33)**	0.033^**^	**1.13 (0.625–2.04)**	**0.689**	**1.88 (1.01–3.47)**	0.045^**^
*cytogenetic BCR-ABL1*	12.9 (2.75–60.1)	0.001^***^	1.73 (0.237–12.7)	0.588				
*cytogenetic CBFB-MYH11*	0.847 (0.307–2.34)	0.749	0.603 (0.188–1.94)	0.395				
*cytogenetic Complex*	1.1 (0.439–2.74)	0.844	1.6 (0.636–4.02)	0.318				
*cytogenetic Intermediate Risk Abnormality*	1.56 (0.788–3.1)	0.201	1.52 (0.741–3.13)	0.252				
*cytogenetic MLL, poor risk*	0.826 (0.202–3.38)	0.791	1.03 (0.25–4.23)	0.969				
*cytogenetic MLL, t(9;11)*	2.55 (0.345–18.8)	0.360	4.01 (0.533–30.1)	0.177				
*cytogenetic Normal*	0.843 (0.514–1.38)	0.497	0.798 (0.471–1.35)	0.399				
*cytogenetic Poor Risk Abnormality*	1.13 (0.407–3.11)	0.819	1.57 (0.564–4.34)	0.389				
*cytogenetic RUNX1-RUNX1T1*	0.41 (0.1–1.68)	0.215	0.463 (0.113–1.9)	0.285				
*FLT3-ITD*	1.41 (0.828–2.4)	0.206	1.12 (0.628–2.01)	0.693				
*FLT3-PM*	**2.05 (0.929–4.53)**	0.075^*^	**2.71 (1.2–6.08)**	0.016^**^				
*CEBPA mutation*	0.663 (0.241–1.83)	0.427	0.708 (0.256–1.96)	0.507				
*DNMT3A/B mutation*	1.54 (0.898–2.64)	0.117	**2.13 (1.22–3.74)**	0.008^***^				
*NPM1 mutation*	0.839 (0.503–1.4)	0.502	0.765 (0.443–1.32)	0.336				
*RUNX1 mutation*	1.39 (0.552–3.51)	0.484	1.89 (0.742–4.84)	0.181				
*TP53 mutation*	2.34 (0.846–6.47)	0.101	**3.62 (1.29–10.2)**	0.015^**^				
*WT1 mutation*	0.988 (0.45–2.17)	0.977	0.733 (0.314–1.71)	0.474				
***SCT***	**0.642 (0.392–1.05)**	0.078^*^	**0.483 (0.284–0.822)**	0.007^***^	**0.497 (0.282–0.874)**	0.015^**^	**0.353 (0.19–0.657)**	0.001^***^

Factors with *P* < 0.10 in the univariate analyses were subjected to multivariate analysis. HR and CI represent hazard ratio and confidence interval respectively

^*^p < 0.10

^**^p < 0.05

^***^p < 0.01

Overall, our findings demonstrate that the *CD69*^+^ HSC-like leukemic subpopulation is present in various subtypes of AML and is associated with primitive phenotypes, unfavorable genetic backgrounds, and poor clinical outcomes.

## Discussion

In this study, we applied scRNA-seq to dissect the cellular heterogeneity in chemo-treated AML patients. Using mouse models transplanted with AML cells from adult patients, previous studies have revealed four chemoresistant features [6–8, 27, 28]. Building upon these findings, we found that in both pediatric and adult AML patients, LSC and OXPHOS expression signatures are mapped onto HSPC-like leukemic populations and are the two major resistance features. Notably, we identified the adhesion molecule *CD69* as a potential biomarker in defining a subpopulation of LSCs that is quiescent and stroma-interacting, and associated with chemoresistance and an increased relapse rate in AML patients.

AML generally consists of immunophenotypically heterogeneous cell populations without universal markers to purify them, even though the often-used CD34 is expressed in only approximately 75% of patients [4]. Thus, AML patient-derived whole BM/PB samples that are routinely utilized in single-cell transcriptomic studies are presented as a complicated mixture of normal and leukemic cells [14, 20, 22, 34]. Unbiasedly distinguishing normal and malignant cells by scRNA-seq represents a unique analytical challenge due to similarities between these cells and the complex differentiation hierarchies in which they reside [35]. Two approaches are used to reliably identify tumor cells in a mixture when the tumor burden is high. A straightforward approach is leveraging genomic mutations (point mutation, fusion gene, or chromosomal copy number variation) detected in full-length and 5’-end scRNA-seq data to annotate tumor cells [9, 22, 36–39]. Alternatively, scRNA-seq data from healthy donors as the reference are integrated into those obtained from samples with high tumor burden. While normal cells cocluster with healthy cells, tumor cells with distinct transcription features form isolated clusters [14, 19, 20]. This alternative strategy has been widely adopted, as high-throughput 10X Genomics scRNA-seq has constrained mutation detection due to 5’- or 3’-biased transcript coverage (Additional file 1: Fig. S2h). Despite these technical improvements, it remains unexplored whether tumor cells can be reliably identified from post-therapy remission samples where the tumor burden is usually low (5%-20%). In the present study, scRNA-seq data of paired pre- and post-therapy samples from the same patient were compiled together with healthy reference using unsupervised clustering. We reasoned that cells from healthy donors and tumor burden-high samples could serve as reliable anchors to distinguish normal and tumor cells, and the AML cells with low abundance presented in the post-therapy samples could be identified by coclustering with the isolated tumor cell population from pre-therapy samples. The feasibility of our approach was validated using van Galen P et al.’s 2019 dataset in which genomic mutation data from third generation sequencing were available (Additional file 1: Fig. S2). Post-therapy leukemic cells defined by this approach were independently confirmed by mutational data and chromosomal aberration-associated expression signatures (Fig. 2).

**Fig. 8:**
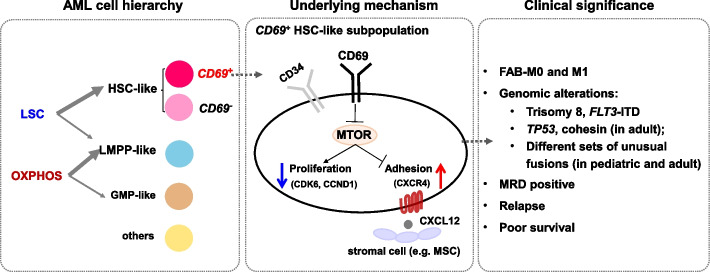
Working model. In AML, HSPC-like leukemic cell populations exhibited one of two known chemoresistance-related expression signatures (LSC and OXPHOS) derived from mouse models (left panel). Among them, HSC-like leukemic cells characterized by the surface marker CD69 possessed chemoresistant capacity possibly via the CD69-mTOR axis. Suppression of the mTOR signaling pathway, in the *CD69*^+^ HSC-like cell subpopulation and *CD69*-overexpressing cell lines, might lead to cell quiescence via suppression of cell cycle regulators CCND1 and CDK6 (as shown in Figs. 5a and 6b, and Fig. S5a) and cell adhesion to stromal cells such as MSCs through the CXCR4-CXCL12 interaction (as shown in Fig. 6d, f). Patients with the *CD69*^+^ HSC-like subpopulation were associated with M0/M1 subtypes and specific genomic alterations and had worse clinical outcomes (right panel)

Chemoresistance has been proposed to be associated with inherent features of LSCs, including metabolic dormancy, self-renewal and BM niche-leukemic cell interactions [4, 40]. As these cellular functions can provide protection from cell cycle-specific chemoagents, LSCs are believed to be the seed cells that mediate disease relapse [41, 42]. However, there is a lack of direct evidence for the capability of metabolically dormant LSCs to survive chemotherapy in either PDX mouse models or patients. Our analysis of primary patient samples provided evidence that a small fraction of *CD69*^+^ HSC-like cells in the diagnostic samples possessed the LSC signature and could persist after intensive chemotherapy in AMLs (Figs. 3d, 4a and 8). This was further supported by the activation of self renewal-associated signaling pathways, including hypoxia and NF-κB in post-therapy HSC-like populations (Fig. 4c). During the follow-up of those patients, one patient relapsed in fifteen months, and another patient remained in partial remission even after the second cycle of induction chemotherapy and went for transplantation at the fourth month. This indicates the long-term persistence of drug-resistant cells. These results were further supported by our analysis of two large public AML cohorts, which showed that the presence of a high percentage of *CD69*^+^ HSC-like cells (*CD69*^+^-high) was associated with significantly higher rates of MRD positivity and relapse, as well as decreased survival rates (Fig. 7e, h and Additional file 1: Fig. S9e, f). Our results correspond with evidence found in chronic lymphocytic leukemia, where the expression of *CD69* also predicted a shorter duration of response and survival [43, 44]. Furthermore, we also revealed that active OXPHOS, a status mostly present in leukemic progenitor cells (LMPP-like and GMP-like), was associated with chemoresistance in these children (Fig. 8). In support of this hypothesis, we found that the heme metabolism pathway was activated in AML cells surviving chemotherapy, which was reported to maintain the electron transport chain during the OXPHOS process (Fig. 4d, e) [45]. Interestingly, active oxidative phosphorylation, but not quiescent dormancy, was recently shown to be a dominant feature in cytarabine-treated AML cells in PDX mouse models [6]. The lack of molecular profiles of LSCs in these mice may be due to the following reasons that may not be mutually exclusive: 1) LSCs were minimally present in the specific patient samples employed in the PDX models. For example, LSC frequency is known to be rare in non-M0 subtypes of AMLs compared with M0 subtypes (2.5% vs. 40%) [46]. 2) LSCs were present in small numbers and were not detected by bulk RNA expression analysis in the study. 3) This chemoresistant model preferentially selected for cells with active OXPHOS. This potential bias may result from species differences, specific chemoregimens applied, and/or retention of a high number of AML cells after treatment.

In addition to the intrinsic properties of HSPCs, resistance to chemotherapy can be mediated by features adaptively acquired during treatment. Upon series cycle application of cytarabine in PDX models, cells that persisted after chemotherapy manifested a senescence-like or leukemia regenerating cell-like feature, and these cells were capable of repopulating leukemia [7, 8]. Intriguingly, the induction of senescence-like cells by chemotherapy was found to be contingent on the stage of therapy and the dosage used, and was often observed in nonremission AML patients, which typically maintained a high tumor burden throughout the therapy with minimal recovery of normal hematopoiesis. However, it remains unclear whether the senescence feature is also present in partial or complete remission patients, in which the proportions of leukemia cells are less than 20% or 5%, respectively, in the bone marrow. In Cihangir Duy’s study, remission samples as a whole, consisting of tumor cells and a large fraction of normal cells, were shown to possess a senescence-like expression signature [7]. In our scRNA-seq analysis of remission patients, examination of the residual AML cells (~ 3.58% of the total population) did not reveal the senescence-like expression signature (Fig. 3d). Interestingly, we did observe this molecular feature in 10 out of 13 patients when the entire population of the remission samples was analyzed as a whole (Additional file 1: Fig. S4d). A plausible explanation for this discrepancy is that normal cells, as a significant portion in the remission sample, could also undergo cellular senescence in response to chemo-agent-induced stress [47]. Nevertheless, given our small pediatric AML cohort, it remains to be investigated whether senescence can be induced in remission patients in future studies. Finally, Boyd et al. revealed that LRC displayed unique characteristics of G-protein-coupled receptor (GPCR) signaling (e.g., *DRD2*, *GRM5*, *and HTR4*) [8]. The features emerged in AML patients approximately 3 weeks following the completion of standard induction chemotherapy. However, we could not perform assessment of the LRC expression signature due to limitations with the sequencing data: more than half of the genes related to GPCR signaling were not detected by 10X scRNA-seq.

Various surface markers, such as CD93, CD69, and CD36, have been found to demarcate distinct subpopulations of immunophenotypically sorted HSC-like cells (CD34^+^CD38^−^) that differ in leukemia initiating activity and cell cycle status [48–50]. In MLL-rearranged AML, CD93 expression marks a discrete subpopulation of immunophenotypic HSC-like cells that actively cycle and are required for leukemogenesis via regulation of self-renewal [51]. Exclusive expression of the adhesion molecule CD69 and the fatty acid transporter CD36 delineate two subpopulations of CD34^+^CD38^−^ HSC-like leukemic cells with varying self-renewal potential and proliferation, respectively: CD69^+^ cells are relatively quiescent and able to self-renew, whereas CD36^+^ cells are highly proliferative but have poor stemness [52]. In extension of these previous studies, our work showed that transcriptomically defined *CD69*^+^ HSC-like cells possess chemo-resistance capacity in pediatric and adult AML patients. Interestingly, we also noted several potential differences between pediatric and adult AML. First, while a high proportion of the *CD69*^+^ HSC-like subpopulation (*CD69*^+^-high) could be used to further stratify the well-known LSC expression scoring-based patient survival, the grouping of patients affected by *CD69*^+^-high was different. Specifically, *CD69*^+^-high could further divide the LSC-defined low-risk group of adult patients, while *CD69*^+^-high led to further stratification of the high-risk group of pediatric patients (Fig. 7d, Additional file 1: Fig. S9d). These differential prognostic values may reflect the distinct stem cell biology associated with specific age groups, such as the self-renewal capacities of LSCs, as well as interactions of LSCs with the BM niche and the immune system [53–55]. Second, the presence of *CD69*^+^-high was associated with specific genomic alterations. Several alterations were common between pediatric and adult AML, including trisomy 8 in the *CD69*^+^-high group and *CBFB-MYH11* fusions in the *CD69*^+^-low group (Fig. 7d, Additional file 1: Fig. S9d). In addition, some genomic alterations enriched in the *CD69*^+^-high group were distinctive between pediatric and adult AML. Mutations in *TP53* and the cohesin genes were observed in the *CD69*^+^-high group of adult AML, while they were nearly absent in that of pediatric AML (Fig. 7d, Additional file 10: Table S9). Furthermore, different sets of unusual fusion genes in the *CD69*^+^-high group were observed between pediatric and adult AML (Fig. 7d, Additional file 1: Fig. S9d). These findings are consistent with the distinct genomic landscape reported in pediatric and adult AML patients [56, 57]. Nevertheless, further studies are warranted to confirm our observations and to explore the underlying regulatory mechanisms.

Furthermore, our regulatory network analysis showed that *CD69*^+^ HSC-like cells may maintain chemoresistance-associated quiescent and adhesive characteristics through repression of mTOR programs (Fig. 5f, Additional file 1: Fig. S5a and Additional file 7: Table S6). This was further validated in AML patient-derived cell lines and primary samples. (Fig. 6, Additional file 1: Fig. S6-7). Overexpression of *CD69* resulted in suppression of the mTOR signaling pathway and promotion of cell quiescence and adhesion in vitro. The functional role of the CD69-mTOR axis in LSC chemoresistance was supported by several studies [58–60]. CD69 inhibition was shown to promote the mobilization and proliferation of HSPCs by inducing mTOR signaling in a mouse model study [60]. Mechanistically, the CD69-mTOR axis enhanced cell adhesion capacity by promoting the CXCR4-CXCL12 interaction. Previous studies have showed that the ability of anti-CD69 to enhance HSPC mobilization was dependent on surface expression of sphingosine 1-phosphate receptor 1 (S1P1) and S1P-S1P1 binding [58, 59], by increasing the release of CXCL12 from BM to the circulation [58]. Nevertheless, future functional studies are needed to demonstrate the ability of *CD69*^+^ HSC-like leukemia cells to mediate chemoresistance and delineate the underlying mechanisms.

## Conclusions

In summary, we investigated the biology of pediatric malignant hematopoiesis over the course of chemotherapy. We revealed the cellular identity and dynamic changes in the molecular properties of chemoresistant leukemic cells in AML. These findings have important implications in designing targeted therapy to eradicate residual chemo-surviving AML cells.

## Material and methods

### Clinical samples

All pediatric AML patients evaluated in this study were enrolled in a randomized, phase III, non-inferiority clinical trial of low- or standard-dose chemotherapy for induction remission (Registration number: ChiCTR1800015883). The low-dose regimen (LDC) was comprised of cytarabine (10 mg/m^2^) and mitoxantrone or Idarubicin, and concurrently administered with G-CSF (5 ug/kg). The SDC regimen was comprised of cytarabine (100 mg/m^2^), daunorubincin, and etoposide. Fresh whole bone marrow (BM) AML samples at diagnosis (pre-therapy) and at the end of the first induction chemotherapy (post-therapy, ~ 26 days of chemotherapy course) were collected from the Children’s Hospital of Soochow University. Fresh BM and peripheral blood (PB) samples from healthy donors were also obtained from the Children’s Hospital of Soochow University. To enrich stem and progenitor cells from fresh PB or BM healthy samples, CD34^+^ cells were sorted using antiCD34 (Miltenyi,130–046-702). All participants in this study provided written informed consent for the sample collection and detailed analyses.

Drug response after each course of induction chemotherapy was evaluated as previously described [61, 62]. Briefly, complete remission (CR) was defined as less than 5% leukemia cells in BM, no leukemia cells in PB and no extramedullary leukemia. CR patients were further classified as MRD (measurable residual disease)-positive CR and MRD-negative CR according to their MRD levels greater or less than 0.1%, respectively. Partial remission (PR) was defined as more than 5% and less than 20% leukemic cells, and at least a 50% decrease in the leukemic burden of pre-treatment BM, and no extramedullary leukemia. No remission (NR) was defined as 20% or more leukemic cells in the BM. Relapse following CR was defined as more than 5% BM leukemic cells unrelated to recovery from the preceding course of chemotherapy or new extramedullary leukemia in patients with previously documented CR. Detailed clinical information was provided in Additional file 4: Table S3.

### Cell lines

Human AML cell lines, HL60 and Kasumi-1, were purchased from Cell Bank of Type Culture Collection Chinese Academy of Sciences (Shanghai, China) and ﻿cultured in RPMI 1640 medium (Hyclone#SH30605.01) containing 10% fetal bovine serum (Gibco), 2 mM L-glutamine (Gibco), 100 units/mL of penicillin, and 100 µg/mL of streptomycin (Gibco). AML cell lines including HL60 and Kasumi-1 were infected with PLVX-CD69 lentivirus to overexpress human CD69 or with PLVX lentivirus as control. ﻿Cells were maintained in a humidified 5% CO_2_ incubator at 37 ℃.

Human MSC cell lines were purchased from Cord Blood Bank of Shandong Province (Shandong Province, China) and cultured in MesenCult™-ACF Plus Medium (Human) from MesenCult™-ACF Plus Umbilical Cord Culture Kit (STEMCELL, Canada). ﻿Cells were maintained in a humidified 5% CO_2_ incubator at 37 ℃.

### Targeted DNA sequencing

Briefly, genomic DNA was extracted from pre-chemotherapy samples of 13 AML patients using the QIAamp DNA Blood Mini Kit according to the manufacturer’s instructions. The DNA sample was quantified by Qubit dsDNA BR Assay kit, and DNA integrity was assessed by agarose gel electrophoresis. DNA was sheared on the Covaris M220 focused ultrasonicator. All libraries were prepared using the KAPA HTP Library Preparation Kit according to the manufacture’s instruction. Fragmented DNA was repaired, 3' dA-tailed, ligated with Illumina adapters, size selected, amplified, and assessed using the Agilent 2100 Bioanalyzer. To detect single nucleotide variation (SNV) and small indel, a customized panel of biotinylated oligoprobes (Roche NimbleGen) was designed to capture all the exons of 1,205 genes that have been identified in previous leukemia sequencing studies (Additional file 4: Table 3). The capture experiment was conducted according to the manufacturer’s protocol. The captured DNA library was finally amplified and sequenced on Illumina Novoseq 6000 sequencer for 2 × 150 bp paired-end reads.

### Somatic mutation calling in targeted DNA sequencing data

Sequence data were aligned to the GRCh38 (hg38) reference genome using BWA-MEM (version 0.7.17) [63], then deduplication and base quality score recalibration were performed using genome analysis toolkit (GATK, version v4.0.11) [64]. SNVs were detected using an ensemble mutation calling approach that considers the union of Mutect2 in GATK and strelka2 tools (version 2.9.10) [65]. As for the only one patient (P117) without normal control, tumor-only mode in Mutect2 was used. Annovar (24 Oct 2019) [66] was used to annotate the mutation sites. SNV and small indel were further filtered out if they either exceeded 1% minor allele frequency in gnomAD database or 1000 Genomes Project and EXAC database, had less than a 5% variant allele frequency (VAF), had less than 20 × coverage depth, or located in non-repetitive regions. Only non-synonymous or splice site mutations were retained for subsequent analyses.

### Single cell RNA-seq library preparation and sequencing

Bone marrow or peripheral blood samples were processed as soon as collected using the red blood cell (RBC) lysis to remove erythrocyte (Beyotime C3702-500ml). To enrich stem and progenitor cells from fresh PB or BM samples, CD34^+^ cells were sorted using anti-CD34 (Miltenyi, 130–046-702). Single cell libraries were prepared using Single Cell 3' Library and Gel Bead Kit V2 (10X Genomics, 120,237) or V3 (10X Genomics, 1,000,075), and Chromium Single Cell B Chip Kit (10X Genomics, 1,000,074) according to the standard manufacturer’s protocols. The quality of the complimentary DNA (cDNA) after reverse transcription and amplification was assessed using Agilent 4200. Libraries were then sequenced on the Illumina Novaseq 6000 Platform (performed by CapitalBio Technology, Beijing).

### Single cell RNA-seq data analysis

Sequencing data were trimmed using Trimmomatic (version 0.36) [67] and Cutadapt [68] to remove the low-quality reads and consecutive As. The scRNA-seq data were aligned to the GRCh38 (hg38) reference genome and quantified using CellRanger (the “count” option; version 3.0.1) that was provided by the 10X Genomics platform. Scrublet (version 0.2) [69] was used to predict doublets in each sample.

Quality control, variable gene selection, dimensionality reduction, and clustering for cells were performed using the Seurat package (version 3.1.5) [13] in R program (version 3.6.0). We removed cells with low quality (< 200 genes expressed, < 500 UMI (unique molecular index), > 15% of the reads mapping to the mitochondria or predicted as doublets), and removed genes that were rarely expressed (< 10 cells). For the remaining cells, expression of each gene in a cell was normalized to the sequencing depth of this cell, scaled to a constant depth (10,000) and log-transformed with the “NormalizedData” function. Cell cycle score was calculated using the “CellCycleScoring” function. Variable genes were selected with default settings. For merging multiple datasets, the integration analysis was applied to remove batch effects caused by different sequencing platforms and experimental processes. Specifically, the “FindIntegrationAnchors” function was firstly used to identify 3,000 anchors with canonical correlation analysis (CCA) dimensional reduction. These anchors were used to integrate the datasets together using the “IntegrateData” function with default parameters. Then, an ‘integrated’ data assay was created for downstream analyses. The “ScaleData” function was used to regress out the differences in cell cycle, the number of genes, and the number of transcripts. Principal component (PC) analysis was performed on the variable genes. The optimal number of PCs for each sample was used for dimensionality reduction if those PCs explained at least 5% of the variance, exhibited cumulative percent greater than 90% and the differences between variations of PCs and a subsequent PC were less than 0.1%. Dimensionality reduction and visualization were performed with the UMAP algorithm using the PCs selected above. Cluster identification mainly relied on “FindNeighbors” and “FindClusters” functions, based on a cosine distance to construct a nearest neighbor graph (Shared Nearest Neighbor, SNN) to group cells. We used built-in “FindMarkers/FindAllMarkers” functions with Wilcoxon Rank Sum test in Seurat (version 3.1.5) to identify differential expressed genes (DEGs) in scRNA-seq analyses. The DEGs were filtered with the percentage of expressed cells, fold change (FC), *p* value, and FDR value.

### Cell type annotation of normal hematopoiesis map

To have a comprehensive characterization of hematopoietic cell types, we collected three publicly available scRNA-seq datasets [10–12]. Samples were further excluded if they were derived from healthy donors with over 60 years old, or if they had UMI less than 2000, genes less than 700, or cells less than 2000. Finally, a total of 23 public healthy donor samples were remained for downstream analyses. Detail clinical information was summarized in Additional file 2: Table S1. All public healthy donors and our healthy donors were integrated and clustered according to the methods described in the “Single cell RNA-seq data analysis” section. This yielded 54 clusters. Then we determined the highly expressed genes in each cluster using the “FindAllMarkers” function in Seurat (described in the “Single cell RNA-seq data analysis” section). We also calculated the pairwise correlations among average expression profiles of different clusters, ranked them by hierarchical clustering, and then identified 20 groups using the “corrplot” function. Clusters were merged if they belonged to a same group in hierarchical clustering. The cell identity of each cluster was inferred if the cells highly expressed known cell type-specific genes.

### Identification of malignant AML cells

Malignant cells are often mixed with normal cells in AML samples, especially for the post-therapy samples. We wanted to distinguish leukemic cells based on their distinct transcription profiles from healthy hematopoietic cells. First, we evaluated the performance of this approach by using published data from Van Galen P et al., in which both high-quality scRNA-seq and mutational genotyping data were available [21]. Processed scRNA-seq data of AML and healthy donors as well as the annotation files were downloaded under GEO accession GSE116256. Seven patients with pre- and post-therapy malignant cells of more than 100 and five healthy donors were included in this analysis. To determine the malignant AML cells, all the cells from paired pre- and post-therapy samples of the same patient together with that of healthy donors were integrated and clustered, using methods as described in the “Single cell RNA-seq data analysis” section. We defined a cluster to be malignant if a high percentage (at least 80%) of the cells in this cluster were from pre-therapy samples. Our predictions were further validated by the presence of somatic mutation and compared with previous classifications using a machine learning algorithm. We found a remarkable agreement between their classifications and our predictions. Next, we identified malignant cells in our AML patients. scRNA-seq data of paired samples (pre- and post-chemotherapy) from each patient were integrated and clustered with that of healthy donors using methods as described in the “Single cell RNA-seq data analysis” section. We classified a cluster as malignant if more than 80% of the cells in this cluster were derived from the pre-therapy sample, while the remaining clusters were classified as normal. For some samples, the malignant cells were revised as the normal cells if they transcriptionally resembled mature lymphoid lineage cells (e.g., B, CTL, and NK). To independently evaluate the malignant and normal predictions, we further identified the cells expressing somatic mutations and the cells showing coexpression of the representative leukemia-associated immunophenotype (LAIP) markers (described in the “Single cell mutation detection” and “Identification of the cells coexpressing representative LAIP markers” section).

### Single cell mutation detection

To accurately identify different types of mutations in single cell, we took advantage of different tools. Firstly, the point mutations (SNV and small indel) identified by targeted DNA sequencing were examined in each cell using the scRNA-seq data of the same patients using VarTrix (version 1.1.16) [70] with default parameters. The numbers and percentages of the mutant cells detected by scRNA-seq were shown in the Additional file 4: Table S3, and Additional file 1: Fig. S2g. Specifically, the mutant cells were detectable in all 13 pre-therapy samples and accounted for a median of 2.29% (range: 0.14%-17.47%) in transcriptionally predicted leukemic cells, which showed a comparable sensitivity in the van Galen P et al.’s 2019 dataset (Additional file 1: Fig. S2g, h). The mutant cells were also detected in post-therapy samples from three patients (P105, P115, and P116) with sufficient cell numbers. A median of 4.20% (range: 2.86%-14.99%) of transcriptionally predicted leukemic cells were found to be mutant cells (Additional file 1: Fig. S2g). Next, chromosomal structural variations were detected in single cell by our in-house pipeline. Briefly, reads mapped to either of the specific fusion gene partners were extracted using SAMtools ‘view’ (version 1.9) [71]. Then, cells were identified as mutant cells with translocation in either of the following situations: (1) cells contained at least one soft clip read around the junction, which could be realigned to the fusion gene pairs simultaneously using blastn (version 2.9.0) [72]; (2) cells contained reads that shared the same UMI but mapped to different fusion gene pairs. In addition, the chromosome Y deletion was determined by a lack of the expression of Y chromosome located-genes. Specifically, we examined the expression of all Y chromosome located-genes [73] that have no homologs on any other chromosomes in scRNA-seq data of healthy donors. Among them, *RPS4Y1* was only one highly and pervasively expressed gene in male while not expressed in female, which made it as a good indicator of the presence of chromosome Y deletion. Thus, we examined the expression of the *RPS4Y1* gene in predicted normal and malignant cells.

### Identification of the cells coexpressing representative LAIP markers

To validate the accuracy of leukemic/normal cell classification, we determined the cells coexpressing representative LAIP markers. First, flow cytometry was used to identify LAIP as previously described [24]. Eleven out thirteen patients had suitable LAIP for defining and monitoring leukemia cells at pre- and post-therapy (listed in Additional file 4: Table S3), which allowed for subsequent validation analyses. Second, cells coexpressing representative LAIP (about 4–5 markers highlighted in Additional file 4: Table S3) were identified using scRNA-seq data if the expression levels of these markers were simultaneously greater than zero. Due to the allele dropout of scRNA-seq, a low fraction of cells coexpressing the representative LAIP were detected. We detected LAIP-coexpressing cells in eleven pre-therapy samples, and a median of 14.32% (range: 4.54%-40.53%) of the predicted leukemia cells coexpressing LAIP markers (Additional file 1: Fig. S2g). Additionally, we also identified the LAIP-coexpressing cells in seven out of eleven post-therapy samples, where a median of 13.33% (range: 2.50%-33.33%) of the predicted leukemia cells coexpressed LAIP markers (Additional file 1: Fig. S2g). The numbers and percentages of the LAIP-coexpressing cells were shown in the Additional file 4: Table S3 and Additional file 1: Fig. S2g.

### Cell type assignment of normal and malignant cells from AML patients

To characterize the heterogeneity of cells from AML patients, we projected all cells from AML patients onto normal hematopoiesis map using the scmap-cell algorithm as implemented in the scmap package (version 1.8) [26] with default parameters. Briefly, the normalized expression data of normal hematopoiesis map were used as input for reference construction. We selected the top 100 highly expressed genes of each cell type according to the average fold change to calculate the expression profile similarity. Cells were projected onto the normal reference and were assigned to the nearest neighbors.

To investigate the performance of our projections, we considered two previously published scRNA-seq datasets, one dataset of sorted healthy BM cells and another dataset with AML and healthy BM cells. We first projected the healthy scRNA-seq data into our normal reference. We found the well-defined cell types showed reasonable agreement for mature cells and less agreement in “HSC” and “LMPP” classifications. We reasoned that this difference could be due to defining discrete populations in a continuous subspace. We then projected their “malignant” AML scRNA-seq data into our normal reference. Next, we applied this projection approach to our AML scRNA-seq data. For the malignant cells, they resembled one of the ten cell types along the HSC to myeloid axis and were named its healthy counterpart with “-like” suffix.

### Gene signature scoring

Fusion gene expression signature scores in single cell profiling were calculated using GSVA (version 1.38.2) [74] or the built-in “AddModuleScore” function in Seurat (version 3.1.5) with default parameters. The signatures were collected from previous publications, including ROSS_AML_WITH_AML1_ETO_FUSION [23], Ng_LSC_positive_2016Nature [28], Farge_HighOXIPHOS_2017CancerDiscovery [6]. The fully normalized FPKM read counts (Fragments Per Kilobase of transcript per Million mapped reads) were log_2_-transformed after incrementing by 1. The LSC score used in survival analyses was calculated for each patient using the scaled data according to the formula in a previous study as follows [28]: LSC score = (*DNMT3B* × 0.0874) + (*ZBTB46* ×  − 0.0347) + (*NYNRIN* × 0.00865) + (*ARHGAP22* ×  − 0.0138) + (*LAPTM4B* × 0.00582) + (*MMRN1* × 0.0258) + (*DPYSL3* × 0.0284) + (*KIAA0125* × 0.0196) + (*CDK6* ×  − 0.0704) + (*CPXM1* ×  − 0.0258) + (*SOCS2* × 0.0271) + (*SMIM24* ×  − 0.0226) + (*EMP1* × 0.0146) + (*NGFRAP1* × 0.0465) + (*CD34* × 0.0338) + (*AKR1C3* ×  − 0.0402) + (*GPR56* × 0.0501). As above- and below-median scores in the published cohorts are associated with adverse and favorable cytogenetic risk, respectively, a median threshold was used to discretize scores into LSC score-high and LSC score-low groups.

### Gene enrichment analysis

Enrichment analysis of DEGs was performed using Metascape (version 3.5) [75], Ingenuity Pathway Analysis (IPA), and Gene set enrichment analysis (GSEA, version 4.0.3) [76, 77] with the default parameters. GSEA was performed to evaluate the activities of chemoresistance-related gene signatures in tumor cell populations. Gene sets were download from the Molecular Signatures Database (MSigDB, version 7.0) of the Broad Institute or publications, and summarized in Additional file 5: Table S4. GSEApy (version 0.10.5) [78] was used to replot the enrichment results and produce publication quality figures. Metascape and IPA were used to evaluate the biological functions of DEGs between tumor and normal cells as well as between HSC-like cells with different chemotherapy responses. Upstream regulator was predicted by DEGs using IPA.

### RNA microarray analysis of CD34^+^CD38^−^ samples sorted from AML patients

We utilized a published microarray expression dataset for CD34^+^CD38^−^ cells from AML patients (GSE76008) [30], to validate the transcription features of chemoresistant HSC-like subpopulations. Each probe set was assigned to a gene using the AnnoProbe package (version 0.1.6) in R (version 4.0.5). For the cases of multiple probe sets representing the same gene, only the probe set with the maximum expression level was assigned to this gene. Fifty-four flow cytometry-sorted CD34^+^CD38^−^ samples from 78 AML patients were divided into the *CD69*^+^CD34^+^CD38^−^ group (expression of *CD69* higher than mean) and the *CD69*^−^CD34^+^CD38^−^ group (expression of *CD69* lower than mean) according to the mean RNA expression of *CD69*. Differential gene expression analysis between these two groups was performed using the limma package (version 3.46.0) [79] in R (version 4.0.5). A list of DEGs was determined if a gene exhibited ≥ 1.3-fold expression level differences (*p* < 0.05), and shown in Additional file 8: Table S7.

### Cell lysis and immunoblotting

AML cells were cultured in complete medium containing 10% serum, stimulated with 25 ng/ml IL6 for 30 min. Cells were harvested, washed with 1 × PBS and lysated with 1 × RIPA lysis buffer containing protease/phosphatase inhibitors (Thermo Fisher #78,441). The lysates were sonicated and centrifuged at 14,000 g for 15min. Western blot analysis was performed with the following primary antibodies, including anti-4EBP1 (CST#9644S), p-4EBP1 (CST#2855P), p-mTOR (CST#5536S), mTOR (CST#2983S), p-STAT3 (CST#9145S), STAT3 (CST#4904S), p-P70S6K (CST#9205S), P70S6K (CST#35,708), CDK6 (Proteintech#14,052), CCND1 (Abcam#ab54503), GAPDH (CST#5174), and HRP conjugated secondary antibodies (Abcam#ab205718, Abcam#ab205719). Detection was conducted using a chemiluminescence substrate (Omics Bio), and images were acquired using ImageQuantTM LAS 4,000 camera and quantified using lmageJ software version1.53 (NIH, Bethesda, MD, USA). To examine whether the phosphorylated protein levels of mTOR and its downstream effectors (P70S6K and 4EBP1) were significantly inhibited, we normalized the levels of phosphorylated protein to the corresponding total protein. Uncropped images for the blots were provided in Additional file 12.

### Flow cytometry and cell sorting

To investigate the correlation of CD69 mRNA and surface protein expression, we sorted CD34^+^CD38^−^ HSC-like populations from bone marrow aspirates of 5 AML patients using flow cytometry and examined the mRNA level of *CD69* by RT-qPCR. Specifically, bone marrow mononuclear cells (BMMCs) were suspended in FACS buffer (1 × Hank’s balanced salt solution with 2% FBS and 0.2% NaN3). Cells were counted using a Countess II Automated Cell Counter. BMMCs were stained with anti-human antibodies: CD34-APC (BD#340441), CD45-PE (BD#561866), CD38-FITC (Biolengend#356610), CD69-APC/CY7 (Biolengend#310913). CD34^+^CD38^−^ cells were sorted and analyzed using FACSAria III and FACSAria SORP cell sorters (BD Biosciences) with FlowJo Software (version 10.4.2). The mRNA level of CD69 were then evaluated by RT-qPCR (described in the “Real-time fluorescence quantitative PCR (RT-qPCR)” section).

To determine the expression levels of adhesion and proliferation-related molecules in HSC-like populations (CD34^+^CD38^−^) from primary AML patients, diagnostic BM aspirates were collected in 2-mL tubes containing ethylenediaminetetraacetic acid (EDTA) and processed by Ficoll density gradient centrifugation to isolate mononuclear cells within 6 h according to the manufacturer’s instructions (GE Healthcare Life Sciences, USA). Blood sample was diluted with an equal volume of PBS plus 2% FBS, added into a SepMate-15 tube (STEMCELL Technologies), and centrifuged at 400 × g for 30 min at room temperature. Enriched mononuclear cells were washed with PBS plus 2% FBS twice and centrifuged at 300 × g for 8 min. Cell count and viability were measured using a Countess II Automated Cell Counter (Thermo Fisher Scientific, USA). Then cells were firstly incubated with anti-CD16/32 for Fc block (1:50, BD Pharmingen™#564220), stained with Live/dye using LIVE/DEAD™ Fixable Dead Cell Stain Kit (Invitrogen#L34957). Then different cell staining was performed to measure the expression of surface proteins and intracellular proteins. 1) To measure the expression of surface proteins, cells were strained with anti-human: CD34-APC (BD#340441), CD45-PE/CY7 (BD#7348679), CD38-FITC (Biolengend#356610), CD69-APC/CY7 (Biolengend#310913), S1PR1 (ABclonal#A3997), CXCR4-Alexa Fluor® 700 (R&D#FAB173N) for 30 min on ice protected from light. The supernatant was then discarded by centrifugation and secondary antibody BV421 (Biolengend#406410) was conjugated with S1PR1 antibody. 2) To measure the expression of intracellular surface proteins, cells were strained with anti-human: CD69-APC/CY7 (Biolengend#310913), CD45-BV785 (BD#563716), CD34-BV421 (Biolengend#343609), and CD38-Alexa Fluor® 700 (Biolengend#303524). Then, cells were fixed and permeabilized using the Foxp3/Transcription Factor Fixation/Permeabilization set (ThermoFisher#00-5523-00). Intracellular surface proteins were stained with different antibodies purchased from Biolegend or Invitrogen (anti-CCND1-Alexa Fluor® 488, 1:100, Abcam#ab190194; anti-Ki67-APC, 1:100, Biolengend#350514; anti-PIM1, Abcam#ab54503; anti-CDK6-CoraLite®594, ThermoFisher#66278).

The protein levels were quantified by mean fluorescence intensity (MFI) in CD34^+^CD38^−^ cells. Cells were analyzed using FACSAria III and FACSAria SORP cell sorters (BD Biosciences) with FlowJo Software (version 10.4.2). We collected data from 21 patients, out of which 19 were successfully evaluated with all candidate molecules and included in subsequent analysis. To investigate the potential relationship between CD69 expression and adhesion/proliferation-related molecules, we adopted a previously described top and bottom 20% grouping method [80, 81] to select CD34^+^CD38^−^ samples for subsequent analyses. Specifically, CD34^+^CD38^−^ samples with CD69 MFI ranking in the top 20% in an ascending order were classified as the "CD69^low^" group (3 out of 19), while those with CD69 MFI ranking in the bottom 20% in an ascending order were classified as the "CD69^high^" group (3 out of 19). The remaining samples were classified as the “CD69^middle^” group (13 out of 19).

### Migration assays

Migration was evaluated using 5.0 um pore-size Transwell assays (Corning Inc, Corning, NY, USA). 2 × 10^5^ AML cells were suspended with serum-free RPMI 1640 medium and cell density was adjusted to 1–10 × 10^4^/mL. 100 μl of the cell suspension was added to the upper chamber of Transwell. 500 μl of complete medium (typically 10% FBS) with a final concentration of 10 ng/ul Recombinant Human SDF-1α (CXCL12, P48061, Peprotech) and 10 uM Sphingosine-1-phosphate(S1P) (HY-108496, MCE) was added to the lower chamber of the 24-well plate. After 3 h of culturing, we removed the cells in the lower chamber and counted the remaining cells using a Countess II Automated Cell Counter. Finally, we calculated the proportion of migrated cells towards a high gradient of CXCL12 or S1P.

### Adhesion assays

We seeded hMSCs in 1 × 10^6^ cells in advance in 10 cm dishes overnight (according to manufacturer's instructions) or 1 × 10^5^ in 6-well plates; Seeded them according to 1 × 10^5^ or 1 × 10^6^ AML cells in the culture system. After 6 h of co-culture, we discarded the supernatant, added 2 ~ 4ml of DPBS to elute adhered cells. Then we digested with 1 ~ 2ml Trypsin–EDTA 0.05% (Fisher Scientific#25-300-120) at 37 ℃ for 1 ~ 2 min, collected cells and counted them again. To detect the adhesion rates of AML cells and human MSC cells, cocultured cells were stained with anti-human antibodies: CD45-APC (Biolengend#304012), CD44-PE (Biolengend#338808), and CD90-PE/CY7 (Biolengend#328124) to measure the proportion of the two types of cells via flow cytometry. We incubated the mixed cells at 4 ℃ for 30 min and detected the percentages of CD45^+^ AML cells and CD44^+^CD90^+^ hMSCs with flow cytometry, and calculated the adhesion ratio of AML cells to hMSCs.

### Real-time fluorescence quantitative PCR (RT-qPCR)

Total RNA was extracted with the TRIzol reagent (Invitrogen; Thermo Fisher Scientific), and cDNA was synthesized using the Revert Aid First Strand cDNA Synthesis Kit (Invitrogen) according to the manufacturer’s instructions. RT-qPCR was carried out in an ABI 7500 instrument (Thermo Fisher Scientific, Singapore, Singapore) using FastStart Universal SYBR Green Master (Rox) (Roche Applied Science, Basel, Switzerland) with primers specific for *CD69* (forward 5'- ATTGTCCAGGCCAATACACATT-3' and reverse 5'- CCTCTCTACCTGCGTATCGTTTT-3'), and *GAPDH* (forward 5'- TGCACCACCAACTGCTTAG-3' and reverse 5'- GATGCAGGGATGATGTTC-3'). The reaction was performed at the following cycling conditions: denaturation at 95 ℃ for 10 min, followed by 40 cycles of 95 ℃ for 15 s and 60 ℃ for 1 min. The relative mRNA expression was calculated after normalization with GAPDH levels using the 2^−ΔΔCt^ method.

### Generation of the expression signatures for 11 leukemic cell identities

We obtained the expression signature for each of 11 leukemic cell types according to the user manual of EPIC, a widely used deconvolution tool. The detailed pipeline was described as follows: In total, 11 leukemic cell types were defined (Fig. 3b and Additional file 1: Fig. S8a), which included *CD69*^+^ HSC-like, *CD69*^−^ HSC-like, LMPP-like, GMP-like, MEP-like, E/B/M-like, CLP-like, monocyte-like, neutrophil-like, cDC-like, and pDC-like. The average expression profile for each type and the standard deviation of expressions were obtained to construct the reference matrix. The top 300 upregulated genes in each type were identified using the “Findallmarkers” function in Seurat (version 3.1.5) (pct > 0.3, FDR < 0.01, *p* < 0.01, and logFC > 0.2). As previously described [22], we examined expression specificity of each upregulated gene in all 11 leukemic cell types, and removed those that were highly expressed in more than two cell types, to ensure that a gene is specific to a certain cell type (and is not highly correlated to another cell type). Finally, a total of 231 genes constituted expression signatures of 11 leukemic cell identities, among which 69.3% have been also identified as cell type-specific genes in previous studies [22, 82]. The list of 231 genes was shown in Additional file 1: Fig. S8a and Additional file 9: Table S8.

### Deconvolution analysis to infer the abundances of leukemic cells

The EPIC algorithm was used to infer the fractions of 11 leukemic cell types in pre-therapy bulk RNA samples. EPIC (version 1.1.5), a widely used deconvolution tool, was obtained from https://github.com/GfellerLab/EPIC, and run with default parameters according to the user manual. Although previous studies have shown that EPIC can accurately quantify different immune cells from solid tumor samples [31], we performed a simulation analysis to evaluate the performance of EPIC on enumeration of different leukemic cells with our 231-gene signatures. Totally 2,529 artificial bulk RNA samples were generated using our scRNA-seq data. In each artificial sample, we pooled each identity of cells at expected ratios (ranging from zero to all) with other types of leukemic cells. We summed the normalized expression profiles of all mixed cells to create an artificial bulk RNA sample. We extrapolated the abundances of 11 leukemic cell identities from these artificial samples by EPIC with default parameters. The accuracy of EPIC on quantification of leukemic cells was evaluated by the correlation between the estimated and known abundances.

We further extrapolated the abundances of 11 leukemic cell types in pre-therapy bulk RNA samples from the TCGA and TARGET cohorts. A total of 296 AML cases (TCGA: *n* = 111; TARGET: *n* = 185) of non-repetitive samples with high blasts (> 60%) were selected for subsequent analyses, to reduce the impact of normal cells to estimate the leukemic cell proportion. EPIC with default parameters was used to infer the fractions of 11 leukemic cell identities in pre-therapy bulk RNA samples. The FPKM normalized bulk expression data were log_2_-transformed after incrementing by 1. The scaled RNA-seq data and the reference matrix of our 231-gene signature were used as the inputs.

### Differential expression analysis of AML patients from TCGA and TARGET

AML-M3 patients were excluded due to great therapeutic success of differentiation therapy replacing chemotherapy. To reduce the influence of ambiguous fractions on subsequent analyses, we separated patients into *CD69*^+^-high (> 25% of total cells are *CD69*^+^ HSC-like) and *CD69*^+^-low (< 10% of total cells are *CD69*^+^ HSC-like) groups. Differential gene expression analysis between the *CD69*^+^-high and *CD69*^+^-low groups was performed using a generalized linear model and the wilcox.test function in the edgeR (version 3.28.1) [83] package in R (version 3.6.0). A list of DEGs for each cohort was obtained if a gene exhibited ≥ 1.3-fold expression level differences (*p* < 0.01 and FDR < 0.05). The DEGs that exhibited consistent expression behaviors between two public cohorts were used in further analyses, which were shown in Additional file 10: Table S9 and Additional file 1: Fig. S9b.

### Statistical analysis

We used corr.test to calculate the correlation between leukemic cell fractions identified by scRNA-seq data and clinical morphological examination. We used Fisher’s exact test for analysis of clinical categorical parameters of TCGA and TARGET samples. A Cox regression model was used for the univariate and multivariate analyses of overall survival (OS) and event-free survival (EFS). Variables used in the univariable Cox regression model included age, gender, stem cell transplantation (SCT), the percentage of bone marrow blast, white blood cell count, MRD status defined by flow cytometry according to the clinical cutoff of 0.1%, cytogenetic karyotype, mutational status of *NPM1*, *FLT3*-ITD/Point mutation (PM) (allelic ratio if available), *TP53*, *RUNX1*, *WT1*, and *CEBPA*, cytogenetic or molecular risk stratifications, LSC score and the proportion of *CD69*^+^ HSC-like subpopulation. Factors with *p* < 0.10 in the univariate analyses without mutually strong correlations were subjected to multivariate analysis. The Kaplan–Meier method was used to estimate the probabilities of OS and EFS, and the log-rank test was used to calculate the *p* values. Statistical analysis was performed using R packages.

### Supplementary Information

**Additional file 1: Figure S1.** Single-cell profiling of normal hematopoietic cells from healthy donors. **Figure S2.** Identification and validation of leukemic and normal cells in published and our scRNA-seq datasets. **Figure S3.** Cell type annotation of leukemia cells by projection onto the hematopoietic hierarchy. **Figure S4.** Expression signatures and dynamic changes of leukemic cell populations in our and published AML scRNA-seq datasets. **Figure S5.** Gene expression profile of *CD69*^+^ HSC-like subpopulation. **Figure S6.** *CD69* overexpression did not affect the STAT3 signaling pathway and the S1P1R expression. **Figure S7.** Suppression of cell proliferation markers and upregulation of adhesion chemokine receptors in CD69^+^CD34^+^CD38^-^ cells sorted from AML patients. **Figure S8. **Estimating of cell type compositions using the EPIC deconvolution. **Figure S9.** Gene expression profiles and clinical outcomes of AML patients with different *CD69*^+^ HSC-like cell proportions.

**Additional file 2: Table S1.** Summary of scRNA-seq data sets used for normal reference construction.

**Additional file 3: Table S2.** List of highly expressed genes (top 100) for each normal cell types.

**Additional file 4: Table S3.** Patient information for all samples in this study.

**Additional file 5: Table S4.** List of chemo-resistance-related gene expression signatures derived from mouse model studies.

**Additional file 6: Table S5.** 117 scRNA Differential Expressed Genes (DEGs) in HSC-like cells between resistant and sensitive groups. These genes met with the differential expression criteria (min.pct ≥ 0.3, log-transformed fold change (FC) ≥ 0.3 or ≤ -0.3, *p* < 10^-10^ and q < 0.01), and exhibited consistent expression behaviors in at least two patients from the same group.

**Additional file 7: Table S6.** Upstream regulators and biological functions enriched by the 117 DEGs in pre-therapy HSC-like between resistant and sensitive groups using Ingenuity Pathway Analysis (IPA). Only those upstream regulators had an overlap *p* value of < 0.05 and a predicted activation state (activated or inhibited, with a z-score > 1.5 or < -1.5) were shown. Up- and downregulation of particular molecules were specified by red and blue arrows respectively. The front color indicated their predicted regulator activation state.

**Additional file 8: Table S7.** 252 bulkRNA DEGs between *CD69*^+^CD34^+^CD38^-^ and *CD69*^-^CD34^+^CD38^-^ groups defined according to expression of *CD69*. DEGs were identified if FC ≥ 1.3 and *p* < 0.05.

**Additional file 9: Table S8.** The estimated proportions of 11 leukemic cell types pre-therapy in the TARGET and TCGA AML patients by EPIC.

**Additional file 10: Table S9.** 368 DEGs in pre-therapy cells between *CD69*^+^-high and *CD69*^+^-low groups. These genes met with the differential expression criteria (FC ≥ 1.3, *p* < 0.01 and FDR < 0.05), and exhibited consistent expression behaviors between two public cohorts.

**Additional file 11: Table S10.** Upstream regulators and biological functions enriched by the 368 DEGs between *CD69*^+^-high and *CD69*^+^-low groups using IPA. Terms with a predicted activated or inhibited state (z-score ≥ 2 or ≤ -2) are shown only when they had an overlap *p* value of < 10^-5^ for upstream regulators, and *p*<0.01 & a network *p* value < 0.01 for biological functions.

**Additional file 12.** Uncropped western blot images.

**Additional file 13.** Review history.

## Data Availability

Targeted DNA sequencing and scRNA-seq data used in this study were deposited into the Genome Sequence Archive for Human at the BIG data center, Beijing Institute of Genomics, Chinese Academy of Sciences and China National Center for Bioinformation under accession number HRA001009 [84], HRA000996 [85], HRA001021 [86]. The release of these data is permitted by The Ministry of Science and Technology of the People’s Republic of China (permission number 2023BAT0907). Published scRNA-seq datasets of healthy donors were available under GEO accession GSE144568 [10], GSE120221 [11], GSE132509 [12], GSE116256 [21]. Bulk RNA-seq data of AML patients were obtained from TCGA [87] and TARGET [88] website. The clinical and genetic information were retrieved from their uploaded supplementary Table S1 [57] and the NCI TARGET website [89] for TCGA and TARGET, respectively. Published microarray expression dataset for flow cytometry-sorted CD34^+^CD38^−^ cells from AML patients was obtained under the GEO accession GSE76008 [30]. The authors declared that all other data supporting the findings of the study are within the paper and its additional files.
