# Supplementary material for: Single-cell transcriptomics reveals multiple chemoresistant properties in leukemic stem and progenitor cells in pediatric AML
Source: Genome Biol. 2023 Aug 31;24:199. doi: 10.1186/s13059-023-03031-7 (PMC10472599; doi:10.1186/s13059-023-03031-7)
Supplement: Supplementary file 1 — Additional file 1: Figure S1. Single-cell profiling of normal hematopoietic cells from healthy donors. Figure S2. Identification and validation of leukemic and normal cells in published and our scRNA-seq datasets. Figure S3. Cell type annotation of leukemia cells by projection onto the hematopoietic hierarchy. Figure S4. Expression signatures and dynamic changes of leukemic cell populations in our and published AML scRNA-seq datasets. Figure S5. Gene expression profile of CD69+ HSC-like subpopulation. Figure S6. CD69 overexpression did not affect the STAT3 signaling pathway and the S1P1R expression. Figure S7. Suppression of cell proliferation markers and upregulation of adhesion chemokine receptors in CD69+CD34+CD38- cells sorted from AML patients. Figure S8. Estimating of cell type compositions using the EPIC deconvolution. Figure S9. Gene expression profiles and clinical outcomes of AML patients with different CD69+ HSC-like cell proportions. [file 13059_2023_3031_MOESM1_ESM.pdf]

Figure S1

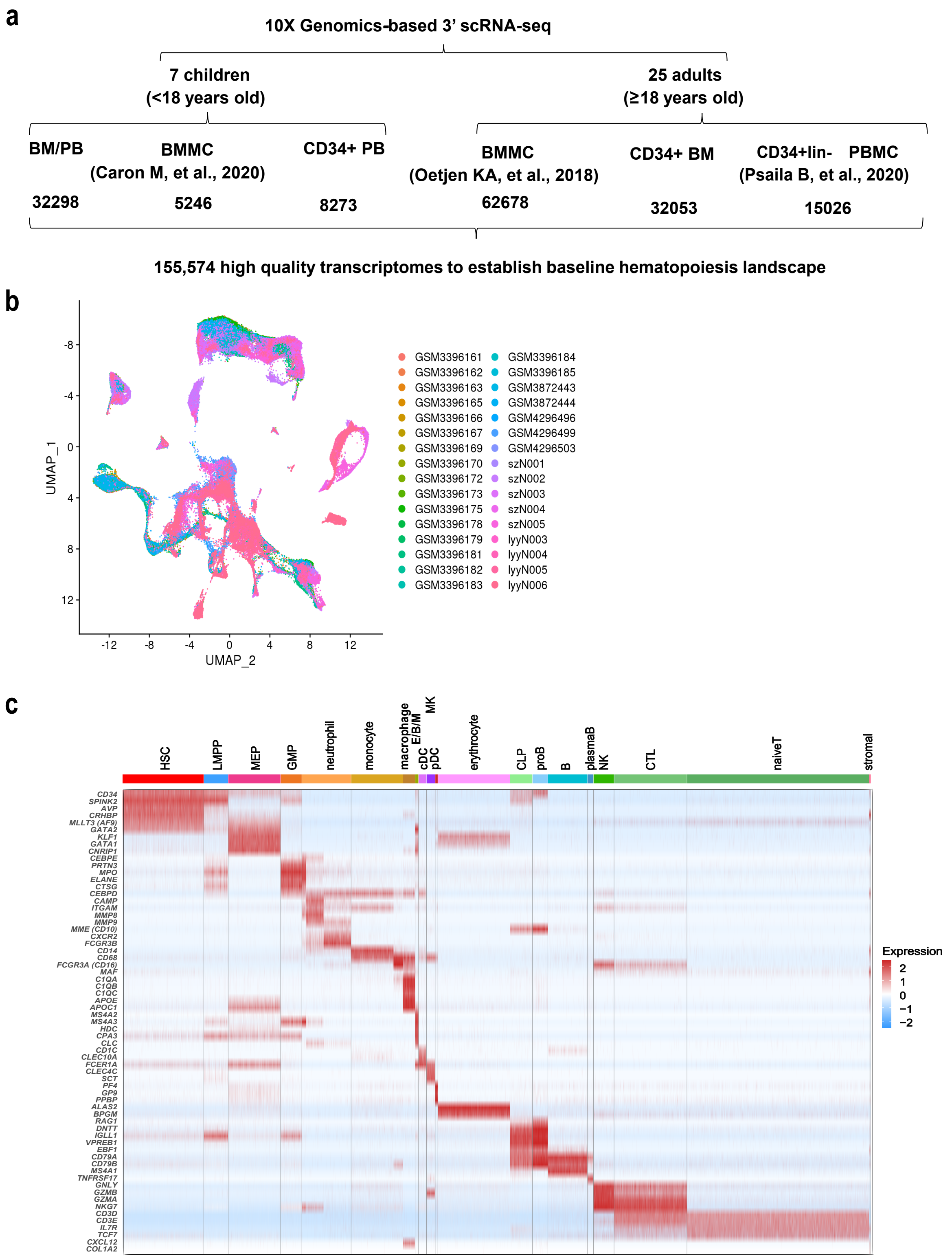

continued on next page

d

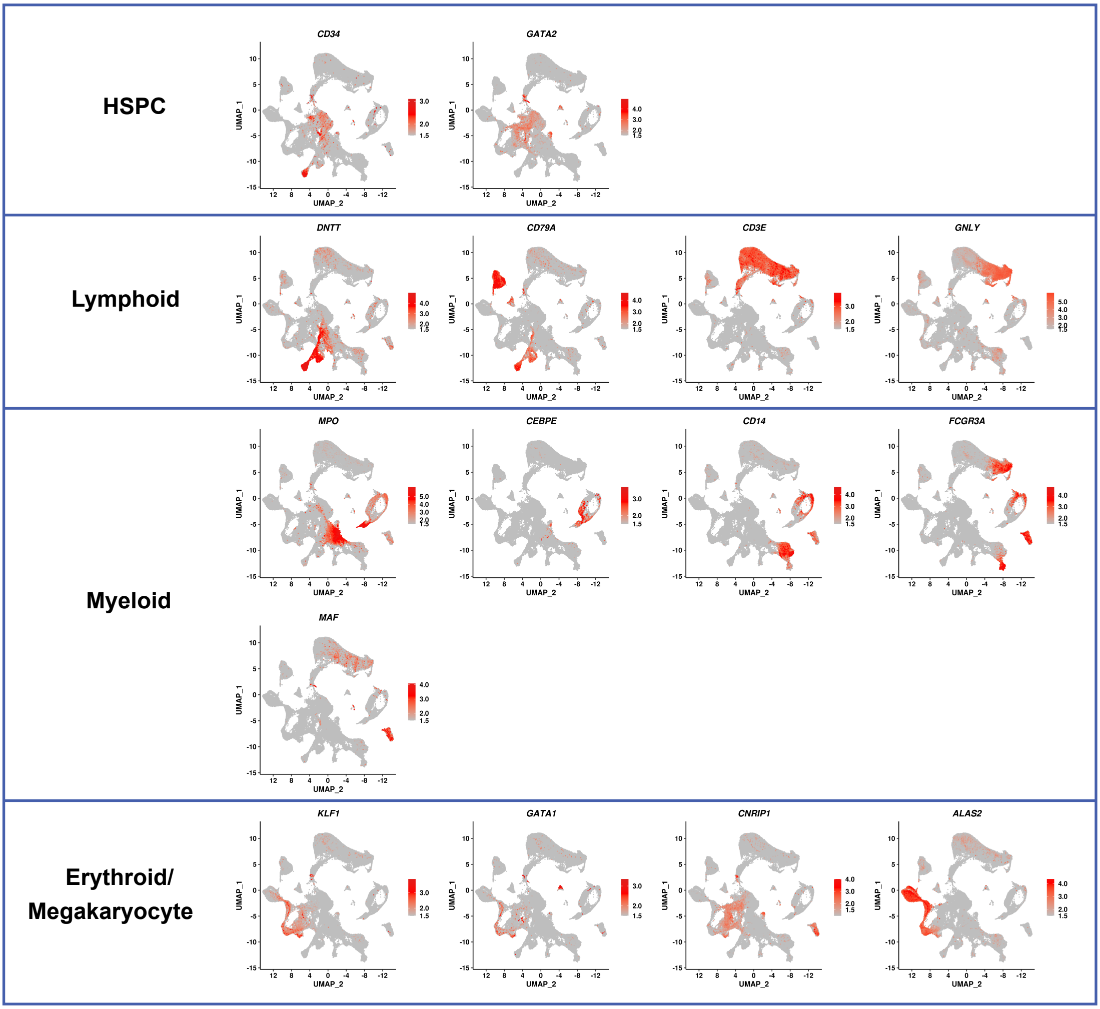
HSPC
CD34
GATA2
Lymphoid
DNTT
CD79A
CD3E
GNLY
Myeloid
MPO
CEBPE
CD14
FCGR3A
MAF
Erythroid/
Megakaryocyte
KLF1
GATA1
CNRIP1
ALAS2
UMAP_1
UMAP_2

e

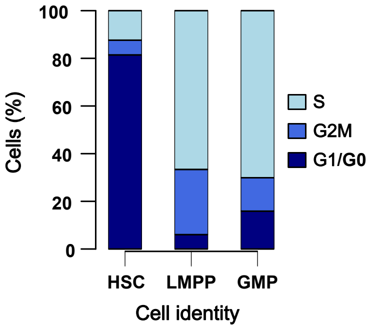
100
80
60
40
20
0
Cells (%)
S
G2M
G1/G0
HSC
LMPP
GMP
Cell identity

Figure S1. Single-cell profiling of normal hematopoietic cells from healthy donors

a Integration of scRNA-seq data from bone marrow (BM) and peripheral blood (PB) aspirates of healthy donors, including our samples and those from published studies. The numbers below each dataset represent high-quality single cells, and the numbers of pediatric and adult donors are indicated. BMMC: bone marrow mononuclear cell. PBMC: peripheral blood mononuclear cell.

b UMAP visualization of healthy donor data, with cells colored according to sample origins.

c Heatmap showing the relative expression of selected cell type-specific marker genes (rows) across all single cells, ordered by cell types (columns).

d Feature plots depicting the expression levels of representative known genes specific to hematopoietic stem/progenitor cells (HSPCs), as well as myeloid, erythroid, and lymphoid lineages in all single cells. Each dot represents a cell, and the shading denotes the relative expression level.

e Bar plot representing the frequency of cell cycle phases in HSPCs.

Figure S2

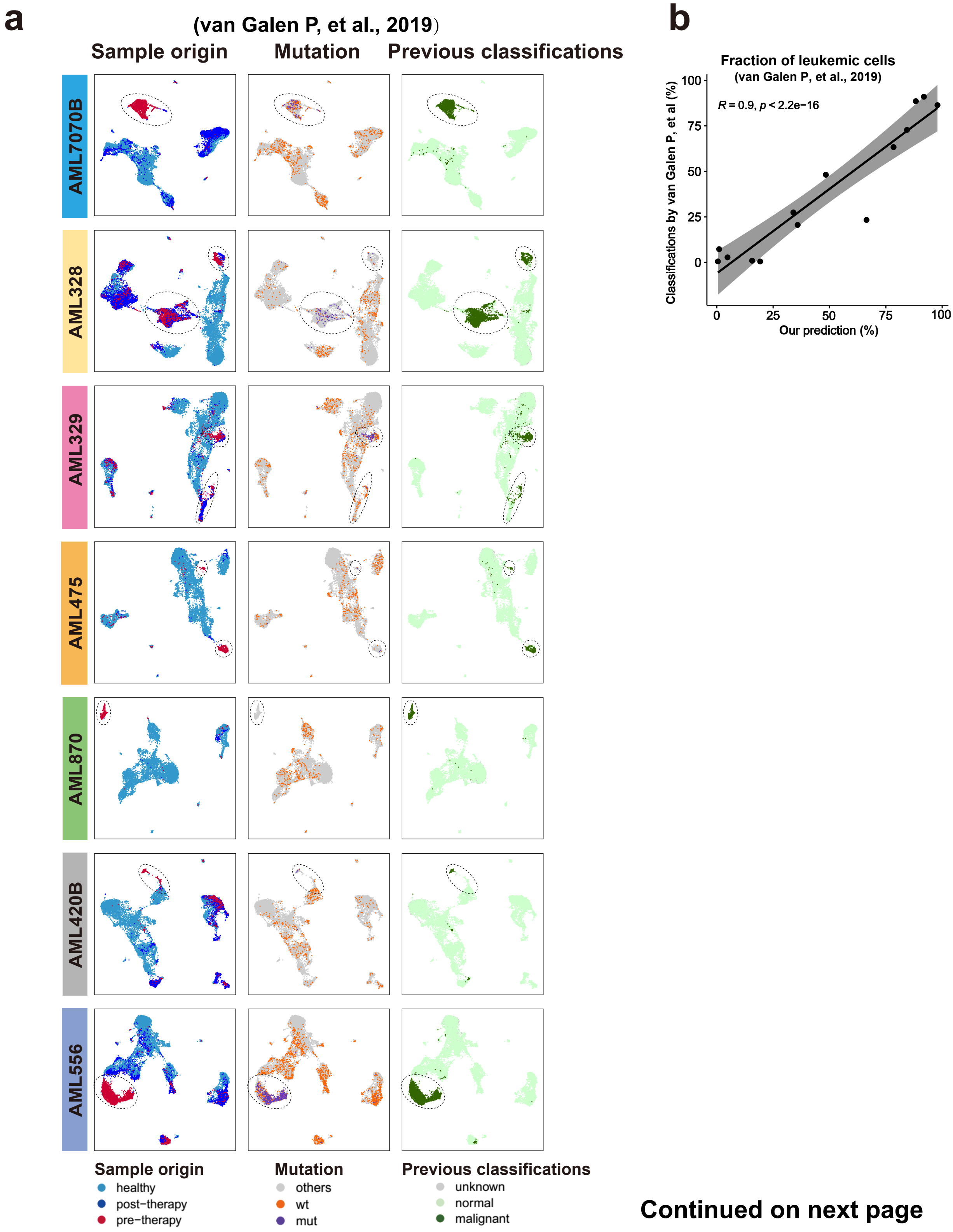

Continued on next page

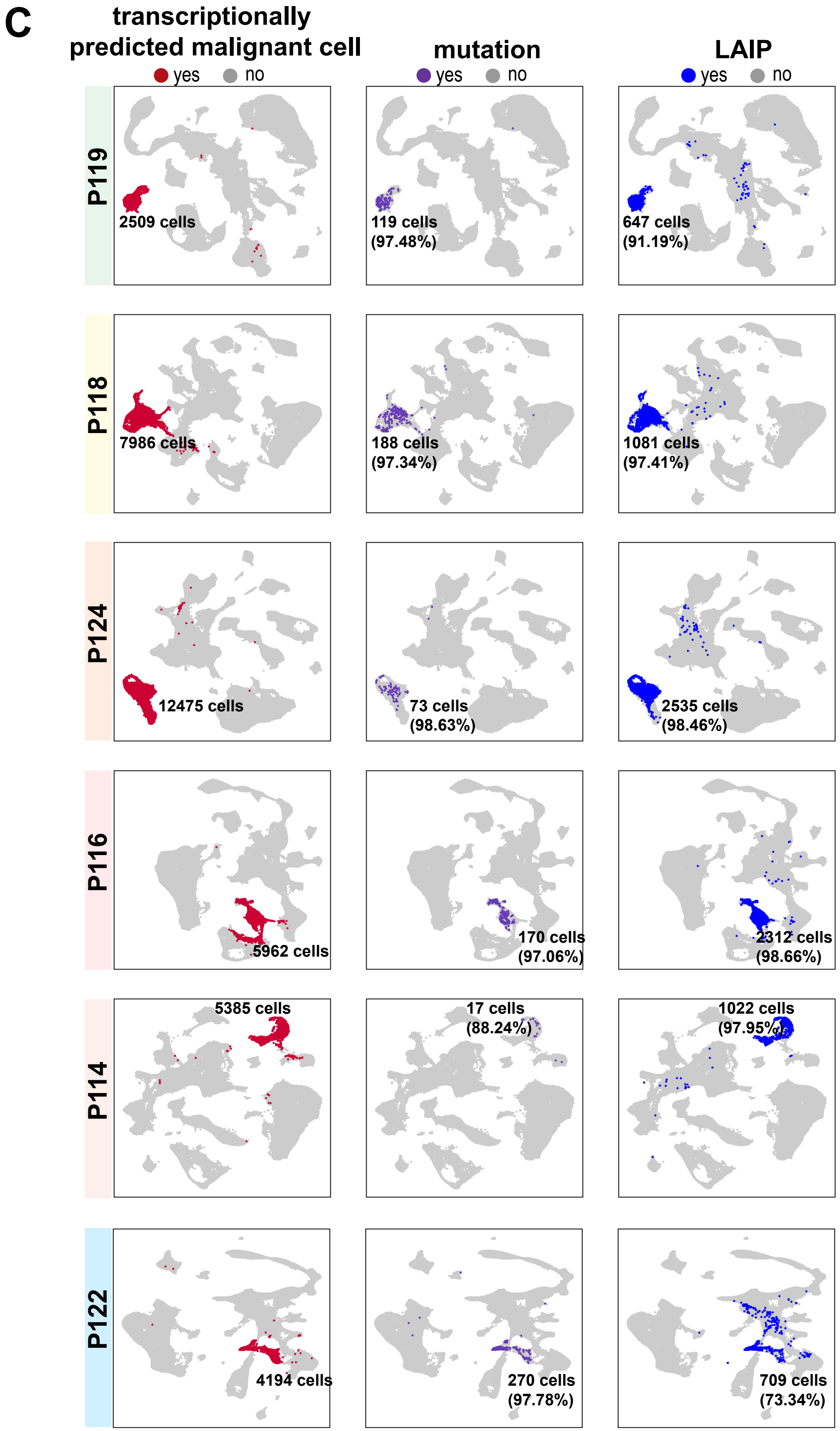

Continued on next page

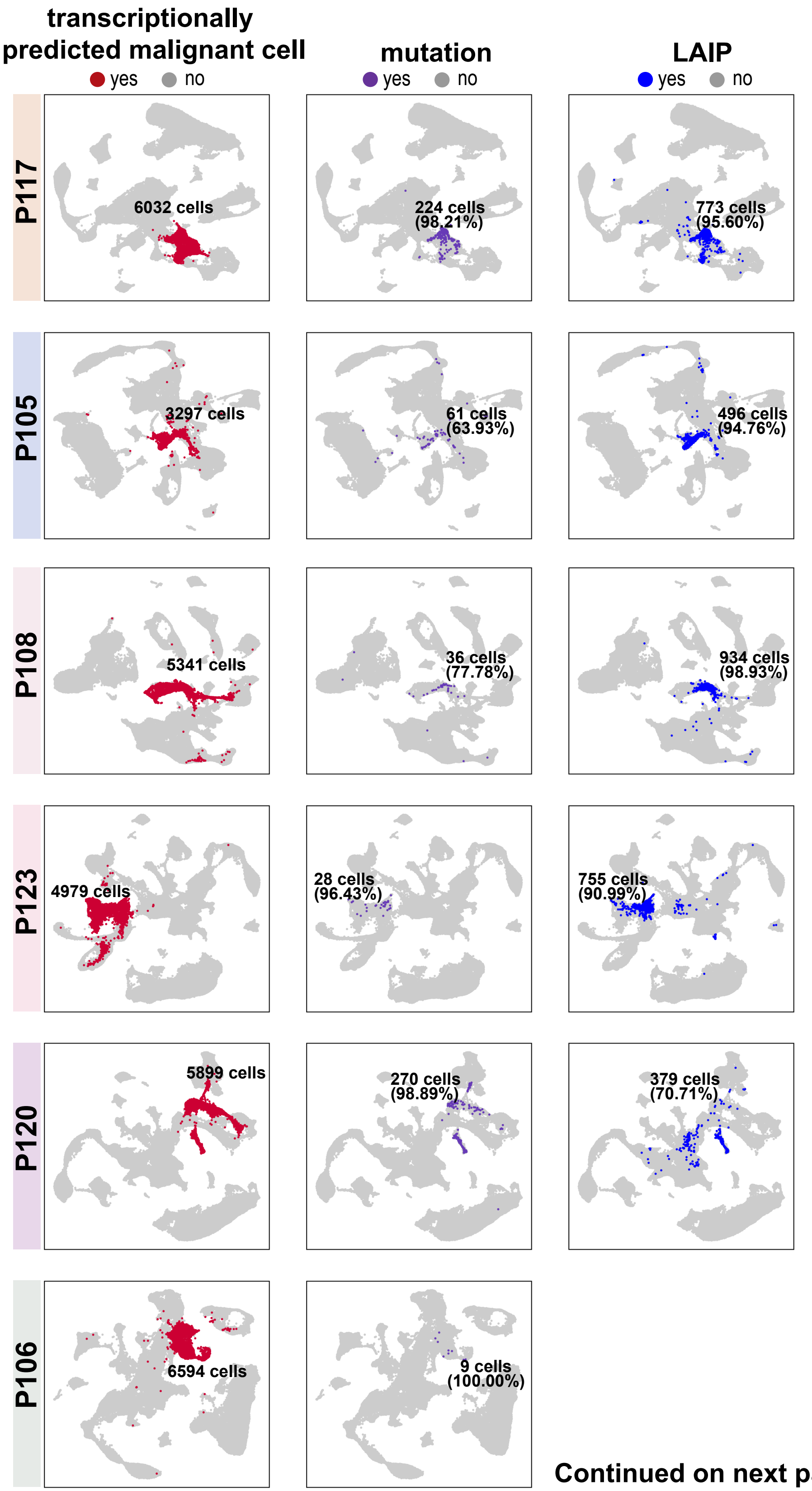

Continued on next page

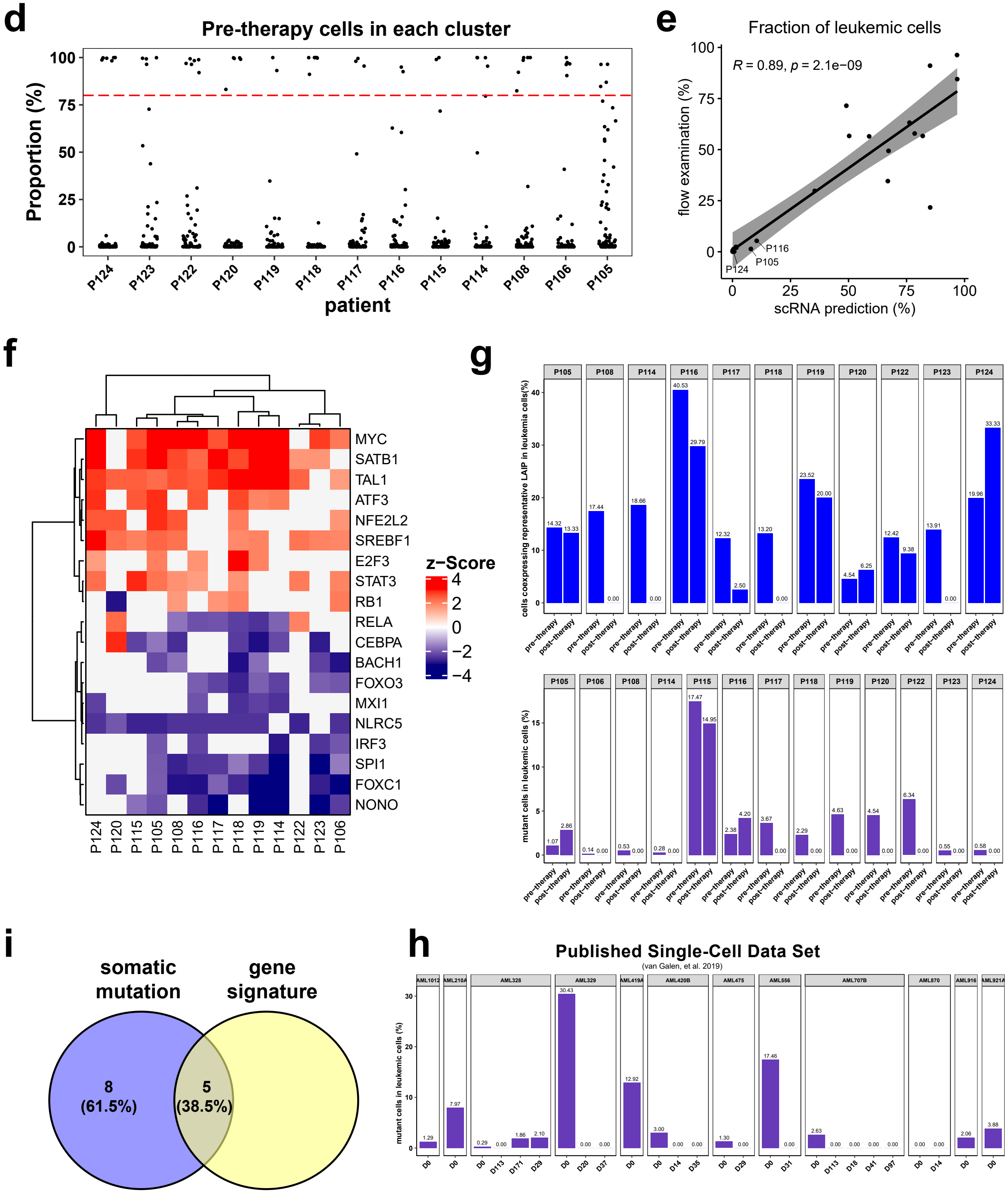
d
Pre-therapy cells in each cluster
Proportion (%)
patient
P124 P123 P122 P120 P119 P118 P117 P116 P115 P114 P108 P106 P105
e
Fraction of leukemic cells
R = 0.89, p = 2.1e−09
flow examination (%)
scRNA prediction (%)
P116
P105
P124
f
MYC
SATB1
TAL1
ATF3
NFE2L2
SREBF1
E2F3
STAT3
RB1
RELA
CEBPA
BACH1
FOXO3
MXI1
NLRC5
IRF3
SPI1
FOXC1
NONO
z−Score
P124 P120 P115 P105 P108 P116 P117 P118 P119 P114 P122 P123 P106
g
P105 P108 P114 P116 P117 P118 P119 P120 P122 P123 P124
cells coexpressing representative LAIP in leukemia cells(%)
pre−therapy
post−therapy
P105 P106 P108 P114 P115 P116 P117 P118 P119 P120 P122 P123 P124
mutant cells in leukemic cells (%)
i
somatic mutation
gene signature
8 (61.5%)
5 (38.5%)
h
Published Single-Cell Data Set
(van Galen, et al. 2019)
AML1012 AML210A AML328 AML329 AML419A AML420B AML475 AML556 AML707B AML870 AML916 AML921A
mutant cells in leukemic cells (%)

Figure S2. Identification and validation of leukemic and normal cells in published and our scRNA-seq datasets

a UMAP visualization of the published healthy and AML scRNA-seq data. Cells are colored according to sample origins (left, malignant cells predicted by our approach are circled), genotypes at specific mutation sites (middle), or previous classifications using a machine learning classifier (right), as indicated at the top.

b Scatterplot comparing the fractions of leukemic cells predicted by our approach and previous classification shown in panel a. Each point corresponds to a sample.

c UMAP plots showing the clustering of paired pre- and post-therapy samples from each patient with healthy donors. Transcriptionally predicted leukemia cells (left), mutant cells (middle), and LAIP-coexpressing cells (right) are highlighted, respectively. The numbers of highlighted cells are shown. Of the highlighted cells, the percentage of cells transcriptionally predicted to be leukemic is shown in parentheses.

d Proportion of cells from the pre-therapy sample in each cell cluster for individual patients. A point represents a cluster. Clusters for each patient were defined by clustering scRNA-seq data shown in panel c and Fig.2b.

e Scatterplot comparing the proportions of leukemic cells predicted by flow examination and scRNA-seq. A point represents a sample. Post-therapy samples from P116, P105, and P124 are indicated.

f Heatmap displaying the upstream regulators enriched by highly expressed genes in leukemic cells compared to normal cells within each patient using IPA.

g Bar plots show the fractions of LAIP-coexpressing cells (top) and mutant cells (bottom) in the predicted leukemic cells in each sample shown in panel c. In the upper panel, only eleven patients with suitable LAIP markers are shown.

h Bar plots show the fraction of mutant cells in the predicted leukemic cells in each sample by our approach shown in panel a.

i Venn diagram shows the number and percentage of AML patients whose predicted leukemic cells were validated by independent methods.

R and p values in panels b and e were calculated using Pearson's correlation test.

Figure S3

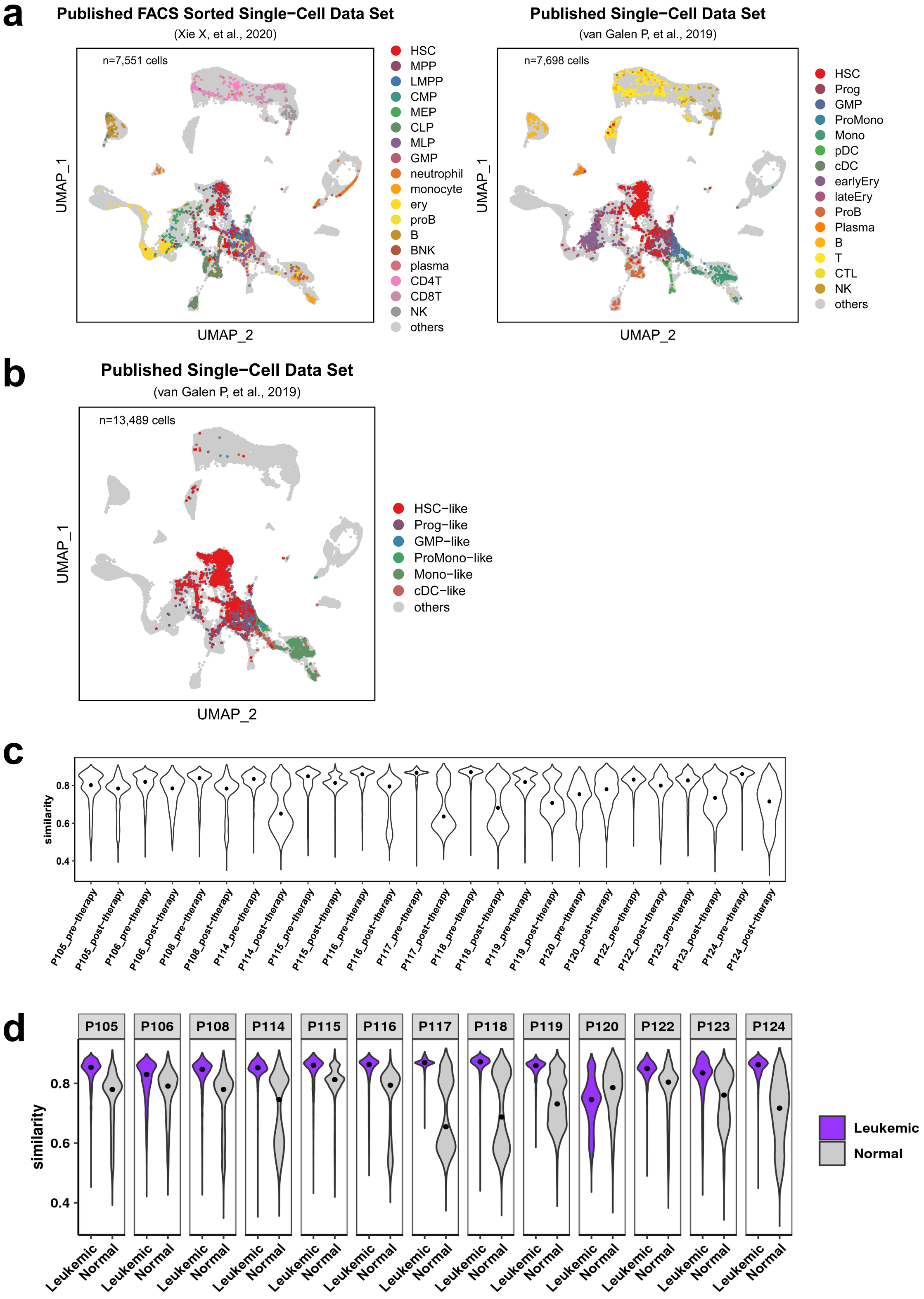

Continued on next page

e

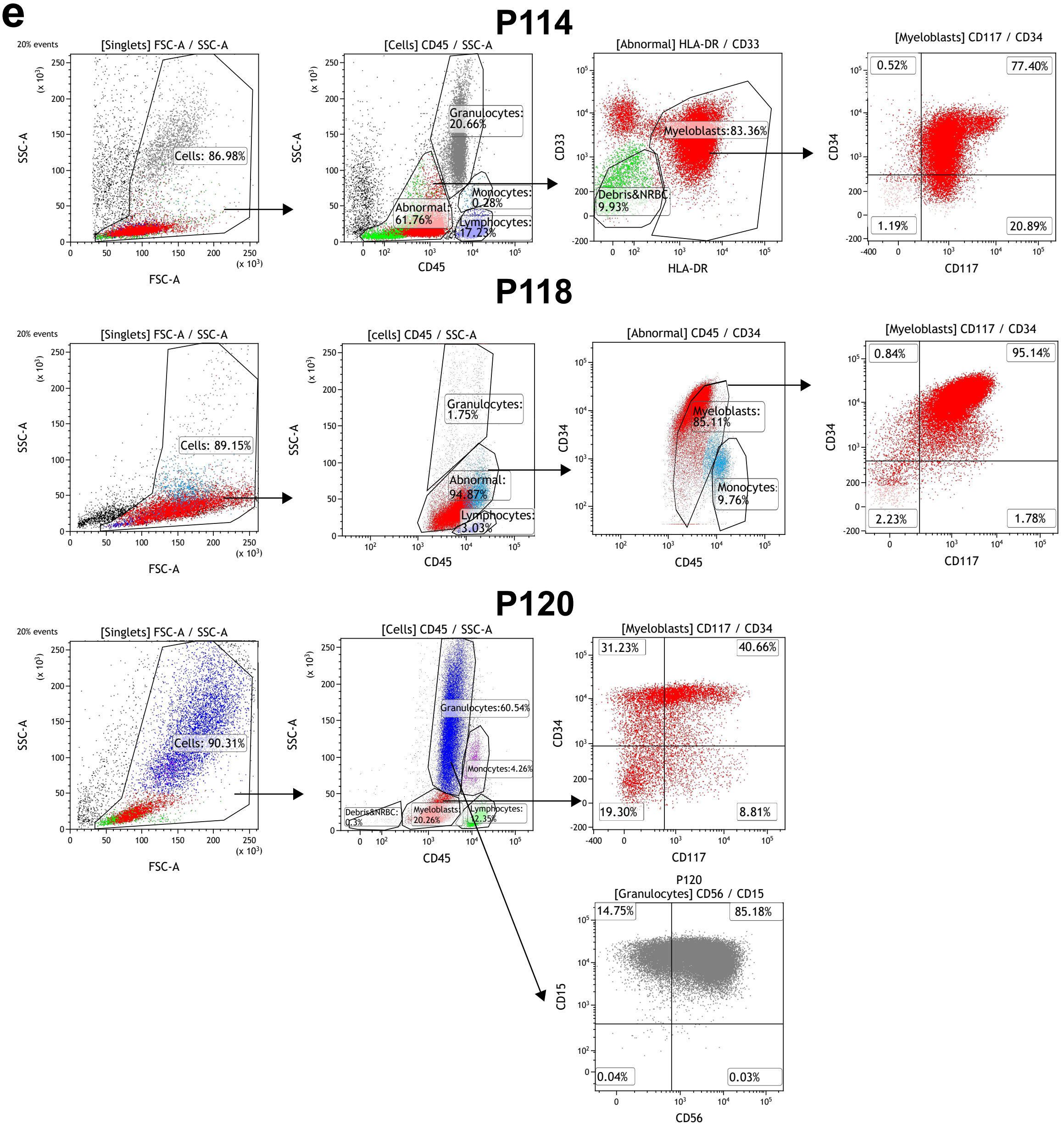

P114
[Singlets] FSC-A / SSC-A
Cells: 86.98%
[Cells] CD45 / SSC-A
Granulocytes: 20.66%
Monocytes: 0.28%
Abnormal: 61.76%
Lymphocytes: 17.23%
[Abnormal] HLA-DR / CD33
Myeloblasts:83.36%
Debris&NRBC 9.93%
[Myeloblasts] CD117 / CD34
0.52%
77.40%
1.19%
20.89%
P118
[Singlets] FSC-A / SSC-A
Cells: 89.15%
[cells] CD45 / SSC-A
Granulocytes: 1.75%
Abnormal: 94.87%
Lymphocytes: 3.03%
[Abnormal] CD45 / CD34
Myeloblasts: 85.11%
Monocytes: 9.76%
[Myeloblasts] CD117 / CD34
0.84%
95.14%
2.23%
1.78%
P120
[Singlets] FSC-A / SSC-A
Cells: 90.31%
[Cells] CD45 / SSC-A
Granulocytes:60.54%
Monocytes:4.26%
Debris&NRBC: 0.3%
Myeloblasts: 20.26%
Lymphocytes: 12.35%
[Myeloblasts] CD117 / CD34
31.23%
40.66%
19.30%
8.81%
P120
[Granulocytes] CD56 / CD15
14.75%
85.18%
0.04%
0.03%
20% events
FSC-A
SSC-A
CD45
CD33
HLA-DR
CD34
CD117
CD15
CD56

Figure S3. Cell type annotation of leukemia cells by projection onto the hematopoietic hierarchy

a, b UMAP plots display the projection of public flow cytometry-sorted healthy hematopoietic single-cell dataset (panel a, left), BMMC single-cell dataset (panel a, right), and malignant hematopoietic single-cell dataset (panel b) onto the hematopoietic hierarchy. Cells from each dataset are colored according to previously published classifications.

c Violin plots show the cosine similarity between cells and their nearest healthy neighbors in each sample.

d Violin plots show the cosine similarity between leukemia/normal cells and their nearest healthy neighbors in each patient.

e Flow cytometry plots show the gating of pre-therapy bone marrow cells derived from AML patients (P114, P118, and P120) and their expression of stem and progenitor markers (CD34 and CD117).

Figure S4

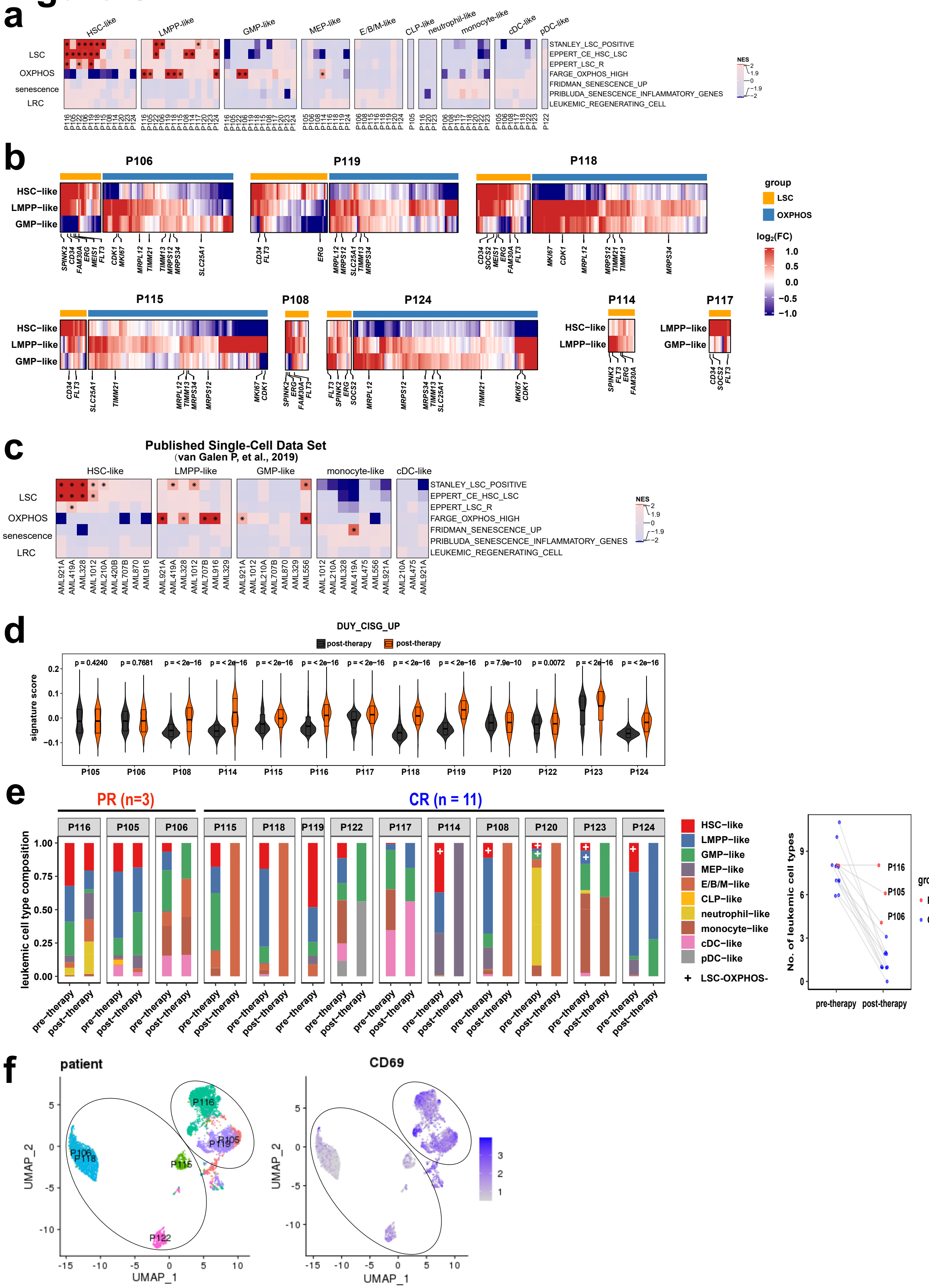
a
HSC-like
LMPP-like
GMP-like
MEP-like
E/B/M-like
CLP-like
neutrophil-like
monocyte-like
cDC-like
pDC-like
LSC
OXPHOS
senescence
LRC
STANLEY_LSC_POSITIVE
EPPERT_CE_HSC_LSC
EPPERT_LSC_R
FARGE_OXPHOS_HIGH
FRIDMAN_SENESCENCE_UP
PRIBLUDA_SENESCENCE_INFLAMMATORY_GENES
LEUKEMIC_REGENERATING_CELL
NES
b
P106
P119
P118
P115
P108
P124
P114
P117
HSC−like
LMPP−like
GMP−like
group
LSC
OXPHOS
log2(FC)
1.0
0.5
0.0
−0.5
−1.0
c
Published Single-Cell Data Set
(van Galen P, et al., 2019)
HSC-like
LMPP-like
GMP-like
monocyte-like
cDC-like
d
DUY_CISG_UP
post-therapy
post-therapy
signature score
P105
P106
P108
P114
P115
P116
P117
P118
P119
P120
P122
P123
P124
e
PR (n=3)
CR (n = 11)
leukemic cell type composition
HSC−like
LMPP−like
GMP−like
MEP−like
E/B/M−like
CLP−like
neutrophil−like
monocyte−like
cDC−like
pDC−like
LSC-OXPHOS-
pre−therapy
post−therapy
No. of leukemic cell types
group
PR
CR
f
patient
CD69
UMAP_1
UMAP_2

Figure S4. Expression signatures and dynamic changes of leukemic cell populations in our and published AML scRNA-seq datasets

a, c Heatmaps depicting the GSEA results of four known chemoresistance-related expression signatures (rows) for each leukemic population (columns) compared to all other leukemic populations within the same patient from our scRNA-seq dataset (panel a) and the published scRNA-seq dataset by van Galen, et al. 2019 (panel c). Colors indicate the normalized enrichment score (NES) values by GSEA analysis, and an asterisk signifies both NES>1.9 and false discovery rate (FDR) <0.001.

b Heatmaps showing expression fold changes (FC) of core enriched genes (columns) contributing to LSC and OXPHOS signatures in each HSPC-like population (rows) compared to all other leukemic populations within each patient. Core enriched genes are identified from GSEA results and those related to cell stemness and metabolism are indicated.

d Violin plot showing the signature scores of senescence in leukemic and normal cells resembling HSC, LMPP, GMP, monocyte, neutrophil, cDC, and pDC. P value for each patient was calculated between paired pre- and post-therapy samples using the Wilcoxon signed-rank test.

e (Left) Bar plot depicting the fractions of leukemic cell types in paired pre- and post-therapy samples from AML patients. A plus sign indicates the cell population that did not exhibit enrichment of LSC or OXPHOS signatures. (Right) Line plot showing the changes in leukemic cellular diversity. Three patients (P116, P105, and P106) who achieved partial remission (PR) are indicated.

f UMAP visualization of pre-therapy HSC-like leukemic cells from resistant patients (P116 and P105) and sensitive patients (P122, P106, P119, P118, and P115). Cells are colored by sample origin (left) and the expression level of *CD69* (right).

Figure S5

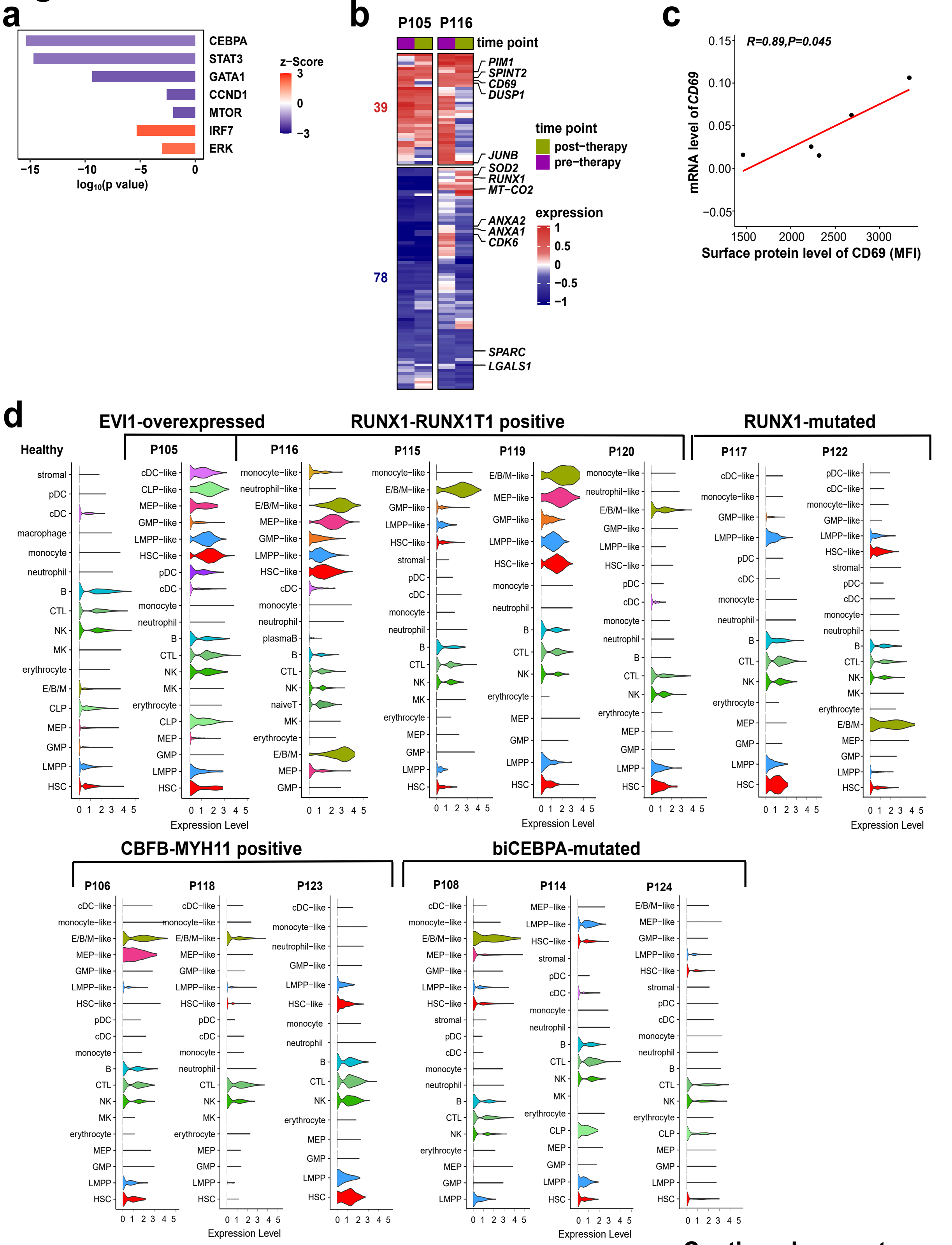

Continued on next page

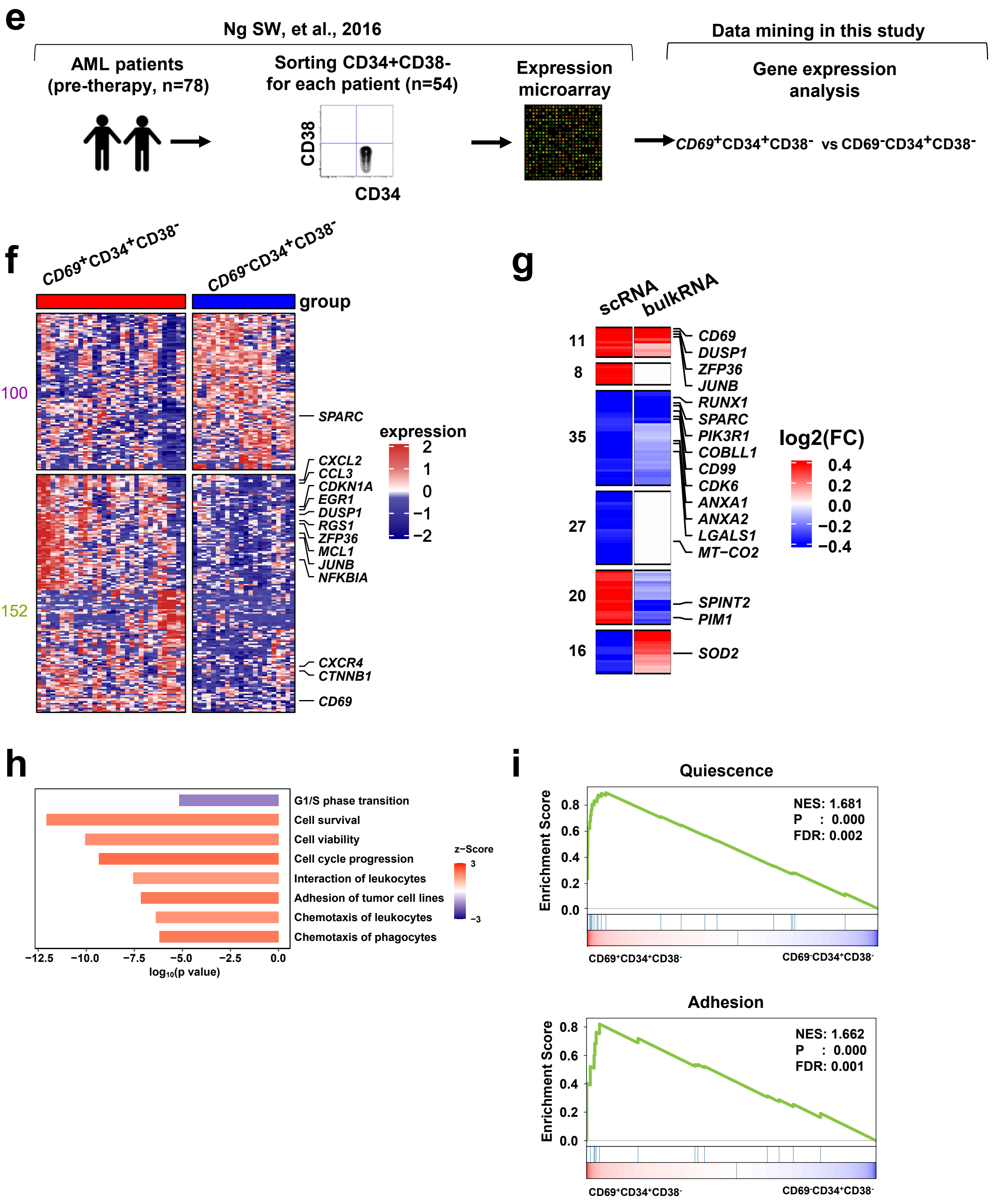
e
Ng SW, et al., 2016
AML patients
(pre-therapy, n=78)
Sorting CD34+CD38-
for each patient (n=54)
CD38
CD34
Expression
microarray
Data mining in this study
Gene expression
analysis
CD69+CD34+CD38- vs CD69-CD34+CD38-
f
CD69+CD34+CD38-
CD69-CD34+CD38-
group
100
152
SPARC
CXCL2
CCL3
CDKN1A
EGR1
DUSP1
RGS1
ZFP36
MCL1
JUNB
NFKBIA
CXCR4
CTNNB1
CD69
expression
2
1
0
−1
−2
g
scRNA
bulkRNA
11
8
35
27
20
16
CD69
DUSP1
ZFP36
JUNB
RUNX1
SPARC
PIK3R1
COBLL1
CD99
CDK6
ANXA1
ANXA2
LGALS1
MT−CO2
SPINT2
PIM1
SOD2
log2(FC)
0.4
0.2
0.0
−0.2
−0.4
h
G1/S phase transition
Cell survival
Cell viability
Cell cycle progression
Interaction of leukocytes
Adhesion of tumor cell lines
Chemotaxis of leukocytes
Chemotaxis of phagocytes
z−Score
3
−3
−12.5
−10.0
−7.5
−5.0
−2.5
0.0
log10(p value)
i
Quiescence
Enrichment Score
0.8
0.6
0.4
0.2
0.0
NES: 1.681
P : 0.000
FDR: 0.002
CD69+CD34+CD38-
CD69-CD34+CD38-
Adhesion
Enrichment Score
0.8
0.6
0.4
0.2
0.0
NES: 1.662
P : 0.000
FDR: 0.001
CD69+CD34+CD38-
CD69-CD34+CD38-

Figure S5. Gene expression profile of *CD69*⁺ HSC-like subpopulation

a Bar plot indicating representative upstream regulators predicted by the "scRNA DEGs" in Fig. 5a using IPA analysis.

b Heatmap showing the average expression levels of the "scRNA DEGs" (rows) in pre- and post-therapy HSC-like populations (columns) of resistant patients. The expression levels are normalized to those of sensitive patients.

c Correlation between mean fluorescence intensity (MFI) and mRNA expression of CD69 in $CD34^{+}CD38^{-}$ HSC-like populations sorted from primary AML patients (n=5). R and P values were calculated using Pearson's correlation test.

d Violin plots depicting the expression of *CD69* in different cell types from healthy donors and AML patients.

e Schematic diagram of bulk gene expression profiling and data analyses for AML patients performed by Ng SW, et al 2016 and this study. The sorted $CD34^{+}CD38^{-}$ cell populations were divided into *CD69*$^{+}$$CD34^{+}CD38^{-}$ (n=32) and *CD69*$^{-}$$CD34^{+}CD38^{-}$ (n=22) groups, based on the mean expression levels of *CD69*.

f Heatmap showing the relative expression of the DEGs between *CD69*$^{+}$$CD34^{+}CD38^{-}$ and *CD69*$^{-}$$CD34^{+}CD38^{-}$ groups ("bulkRNA DEGs").

g Heatmap showing the expression fold changes of "scRNA DEGs" (rows) between the resistant and sensitive groups in Fig. 5a (the scRNA column) and those between *CD69*$^{+}$$CD34^{+}CD38^{-}$ and *CD69*$^{-}$$CD34^{+}CD38^{-}$ groups in panel e (the bulkRNA column). Red and blue indicate a gene with over 1.05-fold increase and decrease in expression, respectively.

h Bar plot showing representative biological processes predicted by the "bulkRNA DEGs" in panel f using IPA analysis.

i GSEA plots showing the enrichment of the quiescence (top) and adhesion (GO term: Positive regulation of leukocyte adhesion to vascular endothelial cells) (bottom) signatures in *CD69*$^{+}$$CD34^{+}CD38^{-}$ cells compared to *CD69*$^{-}$$CD34^{+}CD38^{-}$ cells.

DEGs related to adhesion/migration are indicated in panels b, f, and g.

Figure S6

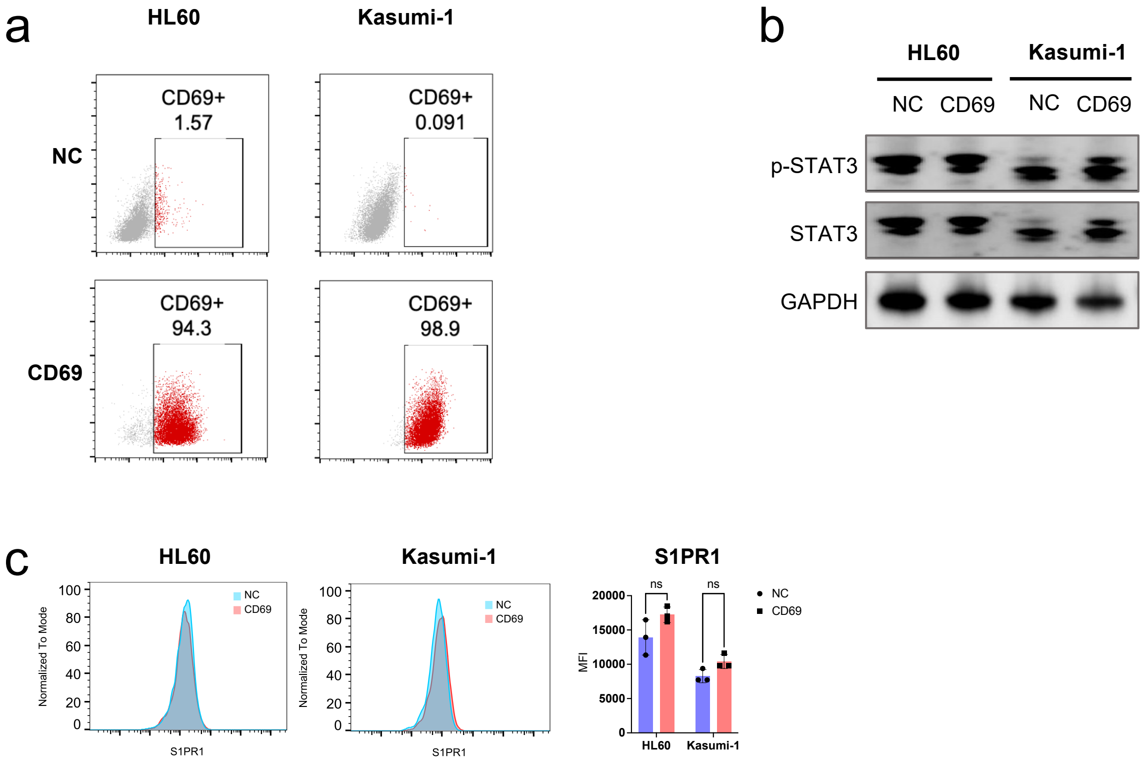

a
HL60
Kasumi-1
NC
CD69
CD69+
1.57
CD69+
0.091
CD69+
94.3
CD69+
98.9
b
HL60
Kasumi-1
NC
CD69
NC
CD69
p-STAT3
STAT3
GAPDH
c
HL60
Kasumi-1
S1PR1
NC
CD69
Normalized To Mode
S1PR1
100
80
60
40
20
0
ns
ns
MFI
20000
15000
10000
5000
0
HL60
Kasumi-1

Figure S6. ***CD69*** **overexpression did not affect the STAT3 signaling pathway and S1P1R expression**

a Flow cytometry analyses of the cell surface protein CD69 on negative control (NC) and *CD69*-overexpressing HL60 and Kasumi-1 cells. Percentages of $CD69^+$ cells within total cells are indicated.

b Western blot showing the total and phosphorylated protein levels of STAT3 in NC and *CD69*-overexpressing HL60 and Kasumi-1 cells.

c Histogram (left) and statistical results (right) of flow cytometry analyses showing the cell surface protein levels of S1P1R on NC and *CD69*-overexpressing HL60 and Kasumi-1 cells. * $P < 0.05$; ** $P < 0.01$; *** $P < 0.001$; ns, not significant; t test. Mean ± SEM values are shown.

Figure S7

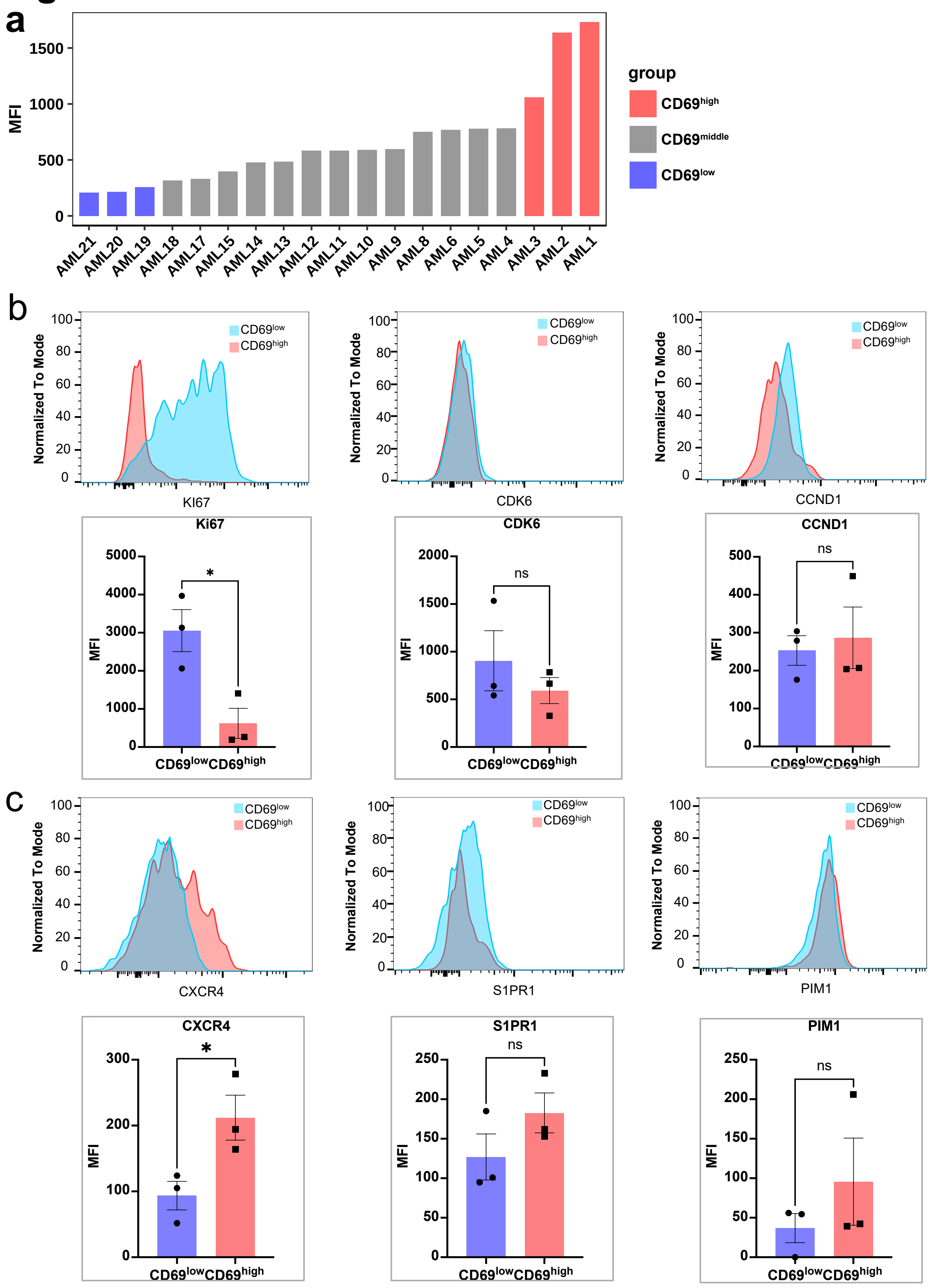
a
MFI
1500
1000
500
0
AML21
AML20
AML19
AML18
AML17
AML15
AML14
AML13
AML12
AML11
AML10
AML9
AML8
AML6
AML5
AML4
AML3
AML2
AML1
group
CD69high
CD69middle
CD69low
b
Normalized To Mode
KI67
CDK6
CCND1
Ki67
CDK6
CCND1
*
ns
ns
CD69low CD69high
c
CXCR4
S1PR1
PIM1
*
ns
ns

Figure S7. Suppression of cell proliferation markers and upregulation of adhesion chemokine receptors in $CD69^+CD34^+CD38^-$ cells sorted from AML patients

a Histogram shows the surface protein levels of CD69 in $CD34^+CD38^-$ (HSC-like) cell populations sorted from pre-therapy BM samples of AML patients (n=19), ranked from lowest to highest. Cell populations with CD69 MFI ranking in the top 20% and bottom 20% were classified as the "$CD69^{low}$" group (n=3) and the "$CD69^{high}$" group (n=3), respectively. The remaining cell populations were classified as the “$CD69^{middle}$" group (n=13).

b Histogram (top) and statistical results (bottom) showing the protein levels of Ki67, CDK6, and CCND1 in the $CD69^{high}$ and $CD69^{low}$ groups in panel a.

c Histogram (top) and statistical results (bottom) showing the protein levels of CXCR4, PIM1, and S1PR1 in the $CD69^{high}$ and $CD69^{low}$ groups in panel a.

* $P < 0.05$; **$P < 0.01$; ***$P < 0.001$; ns, not significant; t test. Mean ± SEM values are shown for panels b-c.

Figure S8

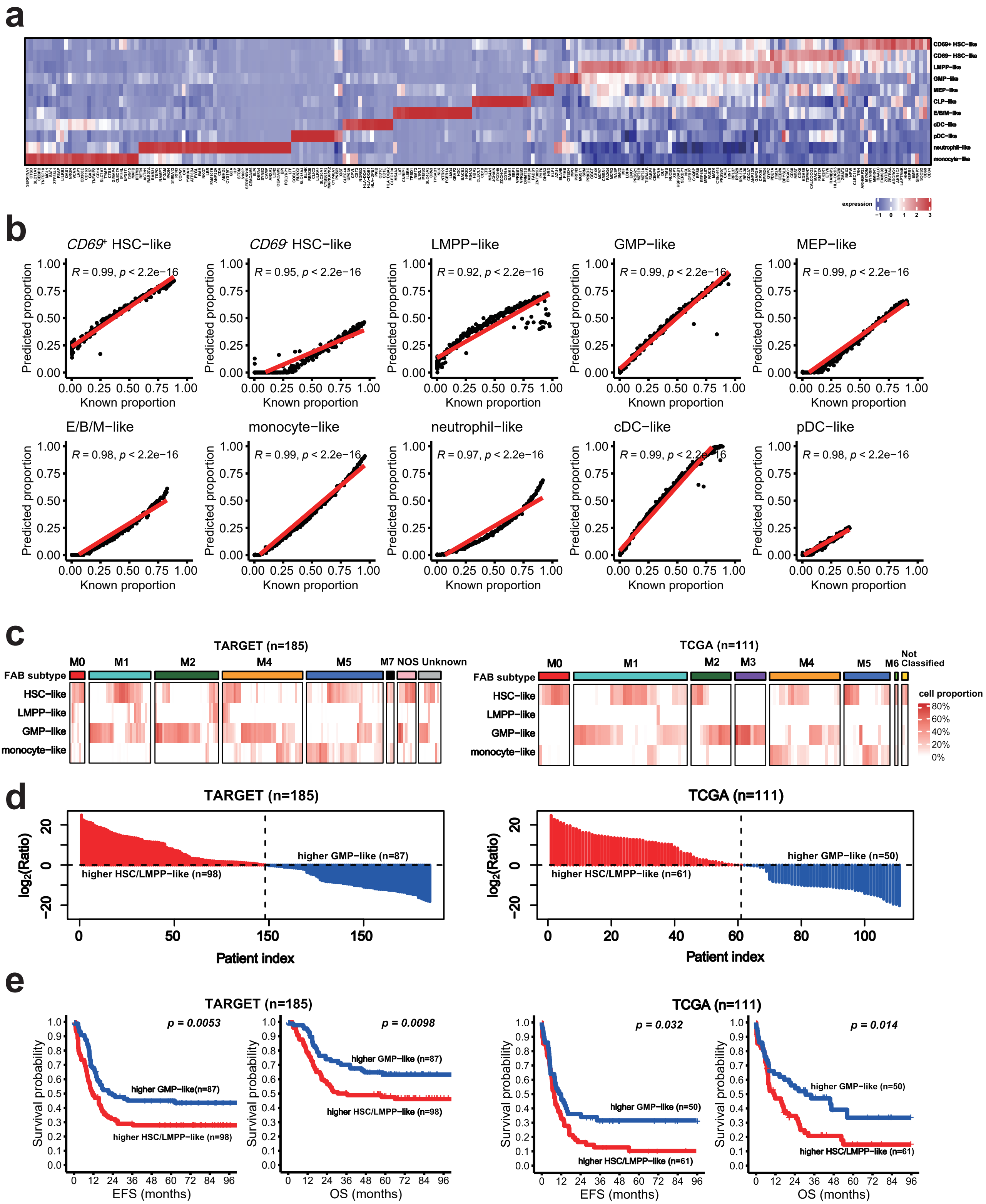

a
CD69+ HSC-like
CD69− HSC-like
LMPP-like
GMP-like
MEP-like
CLP-like
E/B/M-like
cDC-like
pDC-like
neutrophil-like
monocyte-like
expression
−1 0 1 2 3
b
CD69+ HSC−like
R = 0.99, p < 2.2e−16
CD69- HSC−like
R = 0.95, p < 2.2e−16
LMPP−like
R = 0.92, p < 2.2e−16
GMP−like
R = 0.99, p < 2.2e−16
MEP−like
R = 0.99, p < 2.2e−16
E/B/M−like
R = 0.98, p < 2.2e−16
monocyte−like
R = 0.99, p < 2.2e−16
neutrophil−like
R = 0.97, p < 2.2e−16
cDC−like
R = 0.99, p < 2.2e−16
pDC−like
R = 0.98, p < 2.2e−16
Predicted proportion
Known proportion
0.00 0.25 0.50 0.75 1.00
c
TARGET (n=185)
M0 M1 M2 M4 M5 M7 NOS Unknown
FAB subtype
HSC−like
LMPP−like
GMP−like
monocyte−like
TCGA (n=111)
M0 M1 M2 M3 M4 M5 M6 Not Classified
cell proportion
80%
60%
40%
20%
0%
d
TARGET (n=185)
higher GMP−like (n=87)
higher HSC/LMPP−like (n=98)
log2(Ratio)
20
0
−20
0 50 150 150
Patient index
TCGA (n=111)
higher GMP−like (n=50)
higher HSC/LMPP−like (n=61)
0 20 40 60 80 100
e
TARGET (n=185)
p = 0.0053
higher GMP−like(n=87)
higher HSC/LMPP−like (n=98)
p = 0.0098
higher GMP−like (n=87)
higher HSC/LMPP−like (n=98)
TCGA (n=111)
p = 0.032
higher GMP−like (n=50)
higher HSC/LMPP−like (n=61)
p = 0.014
higher GMP−like (n=50)
higher HSC/LMPP−like (n=61)
Survival probability
EFS (months)
OS (months)
0 12 24 36 48 60 72 84 96

Figure S8. Estimation of cell type compositions using the EPIC deconvolution

a Heatmap representing differential average expression profiles of the 231-gene signature in 11 leukemic cell types.

b Scatterplots showing correlations between the inferred (y-axis) and known (x-axis) proportions of transcriptionally-defined leukemic cell types in simulation data generated from our scRNA-seq data. R and p values were calculated using Pearson's correlation test.

c Heatmaps showing the estimated proportions of leukemic cell types in pre-therapy samples of AML patients with different French–American–British (FAB) subtypes from public cohorts.

d The log_2-transformed ratio of estimated HSC/LMPP-like and GMP-like cell proportions within each AML patient in panel c. Patients are ranked by the ratios from highest to lowest and stratified into higher HSC/LMPP-like and higher GMP-like groups.

e Kaplan-Meier curves show the event-free survival (EFS) and overall survival (OS) of AML patients stratified by the ratios in panel d. Patients with higher HSC/LMPP-like proportions have worse outcomes compared to those with lower HSC/LMPP-like proportions. P-values were calculated using the log-rank test.

Figure S9

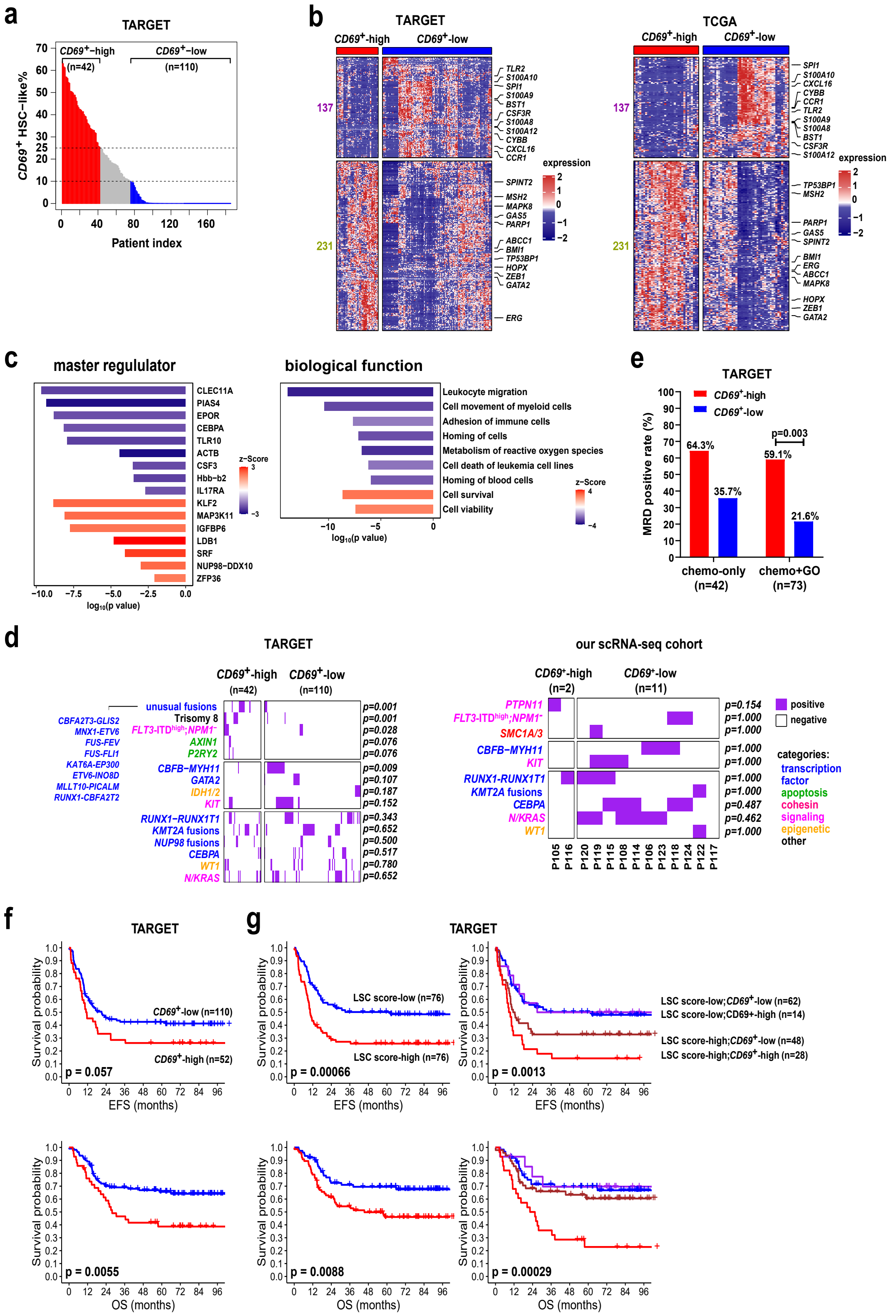

a
TARGET
CD69+-high (n=42)
CD69+-low (n=110)
CD69+ HSC-like%
Patient index
b
TARGET
TCGA
CD69+-high
CD69+-low
137
231
expression
c
master regulator
CLEC11A
PIAS4
EPOR
CEBPA
TLR10
ACTB
CSF3
Hbb-b2
IL17RA
KLF2
MAP3K11
IGFBP6
LDB1
SRF
NUP98-DDX10
ZFP36
z-Score
log10(p value)
biological function
Leukocyte migration
Cell movement of myeloid cells
Adhesion of immune cells
Homing of cells
Metabolism of reactive oxygen species
Cell death of leukemia cell lines
Homing of blood cells
Cell survival
Cell viability
e
TARGET
CD69+-high
CD69+-low
MRD positive rate (%)
64.3%
35.7%
p=0.003
59.1%
21.6%
chemo-only (n=42)
chemo+GO (n=73)
d
TARGET
our scRNA-seq cohort
unusual fusions
Trisomy 8
FLT3-ITDhigh;NPM1-
AXIN1
P2RY2
CBFB-MYH11
GATA2
IDH1/2
KIT
RUNX1-RUNX1T1
KMT2A fusions
NUP98 fusions
CEBPA
WT1
N/KRAS
CBFA2T3-GLIS2
MNX1-ETV6
FUS-FEV
FUS-FLI1
KAT6A-EP300
ETV6-INO8D
MLLT10-PICALM
RUNX1-CBFA2T2
p=0.001
p=0.001
p=0.028
p=0.076
p=0.076
p=0.009
p=0.107
p=0.187
p=0.152
p=0.343
p=0.652
p=0.500
p=0.517
p=0.780
p=0.652
CD69+-high (n=2)
CD69+-low (n=11)
PTPN11
SMC1A/3
p=0.154
p=1.000
p=1.000
p=1.000
p=1.000
p=1.000
p=1.000
p=0.487
p=0.462
p=1.000
positive
negative
categories:
transcription factor
apoptosis
cohesin
signaling
epigenetic
other
P105 P116 P120 P119 P115 P108 P114 P106 P123 P118 P124 P122 P117
f
TARGET
CD69+-low (n=110)
CD69+-high (n=52)
p = 0.057
p = 0.0055
Survival probability
EFS (months)
OS (months)
g
TARGET
LSC score-low (n=76)
LSC score-high (n=76)
p = 0.00066
p = 0.0088
p = 0.0013
p = 0.00029
LSC score-low;CD69+-low (n=62)
LSC score-low;CD69+-high (n=14)
LSC score-high;CD69+-low (n=48)
LSC score-high;CD69+-high (n=28)

Figure S9. Gene expression profiles and clinical outcomes of AML patients with different *CD69*$^{+}$ HSC-like cell proportions

a Estimated proportions of the *CD69*$^{+}$ HSC-like subpopulation (*CD69*$^{+}$ HSC-like%) in pre-therapy samples from TARGET-AML patients. Patients were grouped into *CD69*$^{+}$-high (red), *CD69*$^{+}$-middle (grey), and *CD69*$^{+}$-low (blue) according to the *CD69*$^{+}$ HSC-like%, with dashed black lines indicating the cutoffs.

b Heatmaps showing the relative expression of DEGs in pre-therapy samples between *CD69*$^{+}$-high and *CD69*$^{+}$-low patients in panel a. These DEGs exhibited consistent expression differences between the TARGET and TCGA cohorts. Some representative genes are indicated.

c Representative master regulators and biological functions with dysregulated transcription activities predicted by the DEGs in panel b using IPA analysis.

d Heatmaps showing the presence of genomic alterations in pre-therapy samples from TARGET-AML patients and our scRNA-seq cohort. Genomic alterations (row) are colored according to the biological functions of their corresponding genes. The cohesin term includes mutations of the core complex subunits *STAG2*, *RAD21*, *SMC1A/3/5*, or its modulator *PDS5B*. *FLT3*-ITD^{high};*NPM1*$^{-}$ represents wild type *NPM1* with *FLT3*-internal tandem duplication (ITD) with a high allelic ratio (≥ 0.5). Unusual fusions are indicated.

e Flow cytometry-based measurable residual disease (MRD) positive rates in TARGET-AML patients at the end of the first cycle of chemotherapy regimen. Patients were defined as MRD-positive with the clinical cutoff of 0.1% at the end of the first cycle of induction therapy. Chemo-only and chemo+GO represent patients who received standard chemotherapy without or with gemtuzumab ozogamicin (GO), respectively.

f Kaplan-Meier curves showing the survival of TARGET-AML patients stratified by *CD69*$^{+}$ HSC-like% in panel a.

g Kaplan-Meier curves showing the survival of TARGET-AML patients stratified by LSC score alone or combined with *CD69*$^{+}$ HSC-like% in panel a.

All p values in panel d were calculated using Fisher’s test.

All p values in panels e, f and g were calculated using the log-rank test.
