# Supplementary figures and images for: Single-cell transcriptomics reveals multiple chemoresistant properties in leukemic stem and progenitor cells in pediatric AML
Source: Genome Biol. 2023 Aug 31;24:199. doi: 10.1186/s13059-023-03031-7 (PMC10472599; doi:10.1186/s13059-023-03031-7)

# Additional file 12. Uncropped western blot images

## Main Fig6a

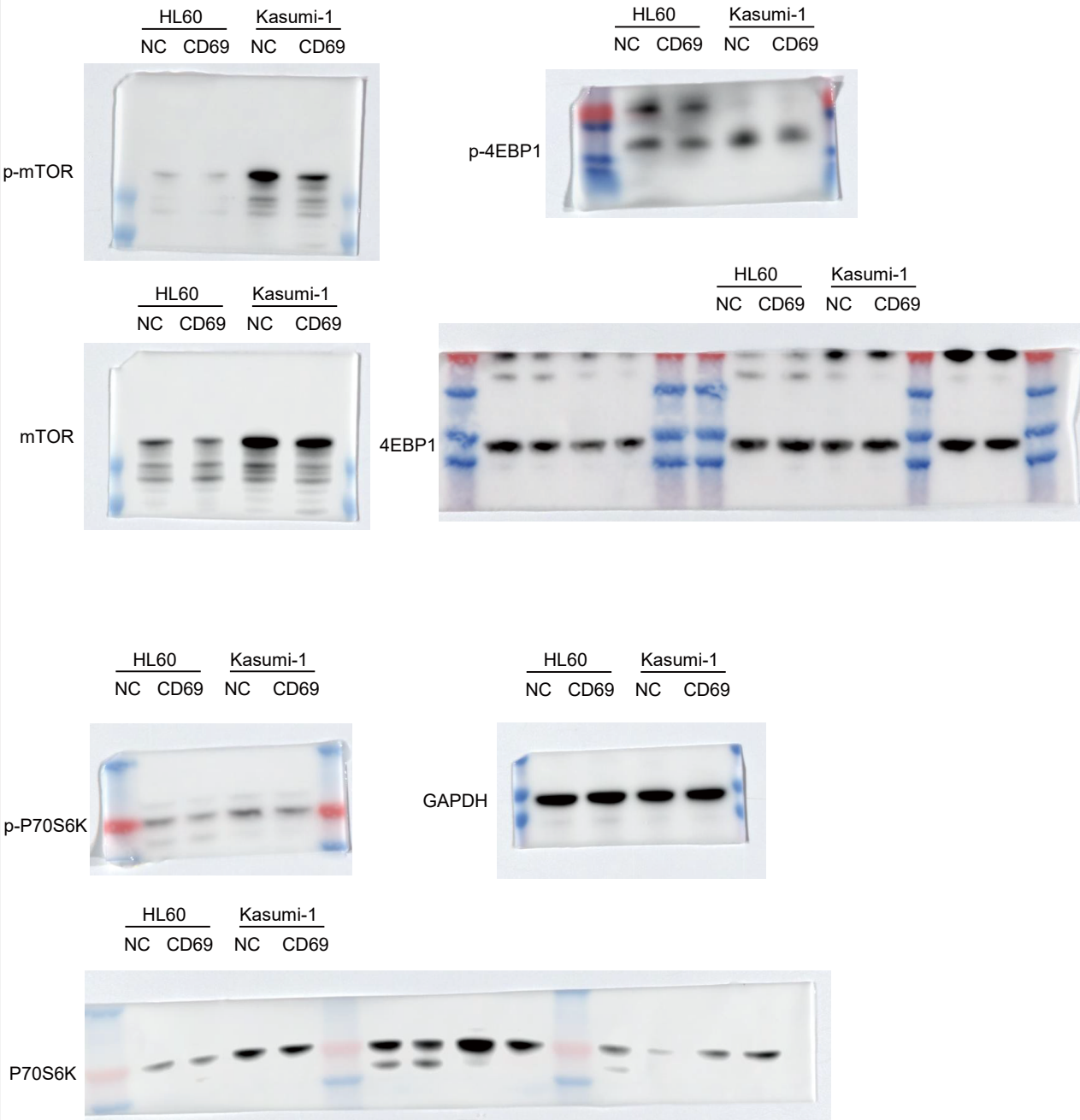

Main Fig6c

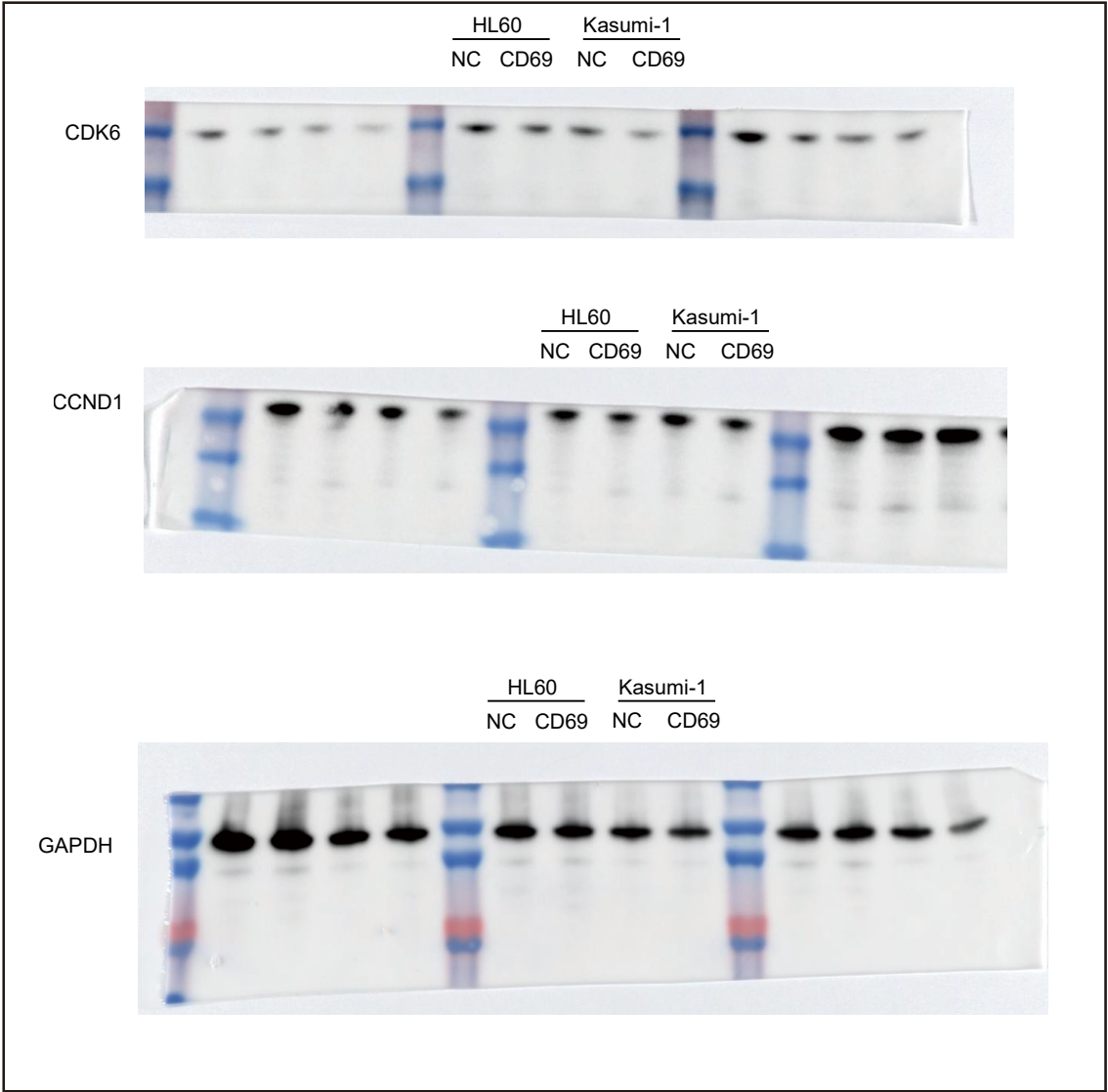

Supplement: Supplementary file 12 — Additional file 12. Uncropped western blot images. [file 13059_2023_3031_MOESM12_ESM.pdf]
