# Supplementary material for: Single-cell transcriptomics reveals multiple chemoresistant properties in leukemic stem and progenitor cells in pediatric AML
Source: Genome Biol. 2023 Aug 31;24:199. doi: 10.1186/s13059-023-03031-7 (PMC10472599; doi:10.1186/s13059-023-03031-7)
Supplement: Supplementary file 13 — Additional file 13. Review history. [file 13059_2023_3031_MOESM13_ESM.docx]

Review History

**First round of review**

**Reviewer 1**

**Are you able to assess all statistics in the manuscript, including the appropriateness of statistical tests used?** Yes, and I have assessed the statistics in my report.

**Comments to author:**

In their study "Single-cell Transcriptomics Reveals Multiple Chemo-Resistant Properties in Human Leukemic Stem and Progenitor Cells" the authors present a single cell study focusing on pediatric AML. The authors include also healthy BM controls that they generate, together with previously published profiles. The main methodology is single cell RNA-sequencing and the authors have included proper benchmarks to validate their approach to detect leukemic cells based on transcriptome clustering, and later to estimate their proportions of specific cell clusters from bulk studies. To support findings from the pediatric cohort characterized in this study (N=13), the authors re-analyse and integrated published dataset to add strength to their conclusions. The presented study focuses on AML and the study design and methodology follow established practices. It appears focused towards the field of leukemia, not broader audience.

The authors have examined two known chemo-resistance-related signatures identified from previous mouse studies. They demonstrate that these correspond to different cell populations in primary patient samples, as identified by scRNA-seq. Several interesting genes and pathways are highlighted by the analysis that could serve as prognostic biomarkers of cell populations in resistant cells. Among these the CD69+ stem-like cell population has also been recognized by earlier studies of normal bone marrow progenitors. Results on CD69+ cells found from the single cell AML profiles are interesting, however in part unclear to this reviewer, as outlined more in detail below, and additional experimental support would be needed to substantiate the relevance of this marker as a biomarker of drug resistance.

Major comments:

1) The description of material used should be more detailed regarding the patients included to this single cell study. The Methods section describes that participants belong to a clinical trial of low- or standard-dose chemotherapy for induction. Which treatment did these cases receive? More specifics on the drugs and their dosing regime should be provided since the definitions of resistance signatures and comparison of cases by drug response are central to this study.

2) The analysis of normal BM clusters in fig1A is used to guide the matching of leukemic cells later in the analysis. Overall, the analysis revealed expected cell populations. However, what is the E/B/M cluster? Later, the authors focus on HSC-, GMP- and LMMP-like populations, but there is one case where mainly all leukemic cells matched this cell cluster which is not described. In addition, it would be important to include a UMAP figure panel that shows the matching of cells to different donors since it is relevant for evaluating that technical batch effects are not having a major impact on cell clustering.

3) The authors show representative UMAP generated with healthy donor cells and leukemic samples from pre- and post-treatment (in Fig. 2) and compare leukemic cells to healthy bone marrow based on similarity to normal cell transcriptomes using scMap tool (Fig. 3). Based on the example case, leukemic cell transcriptomes are distict from their normal BM counterparts as they segregate in Fig. 2. How well matched are the cells based on the three correlation metrics that scmap calculates (cosine similarity and Pearson and Spearman correlations)? What percentage of cells (by sample) were matched by the criteria proposed by the tool developers (in the scmap publication "We required that at least two of the similarities be in agreement, and that at least one be >0.7 If these criteria were not met, then c was labeled as "unassigned" )? How well were the non-leukemic cells matched in comparison to the leukemic? This further clarification is important since majority of downstream analyses are based on the distinction of HSC-, GMP and LMMP-like cells and comparing their properties across cases and there is always uncertainty in matching cells between experiments and this is more challenging when matching cells from disease to healthy reference. The current evaluation of success is based on comparison to clinical morphology (M1-M7 categories). The authors argue that mainly there is agreement, but do not discuss what could underlie the discrepancies observed (P114 and P118 belong to M4/M5 categories but have few monocyte-like cells matched; in contrast M2 phenotype P120 has mainly monocyte-like cells. Do the authors have additional (e.g. flow cytometric by CD34 vs CD14 and other stem- vs monocyte markers ) data to ascertain which assignments are more accurate regarding blast phenotype?

4) More clarification is needed to interpret panel 3d. The color in this heatmap corresponds to enrichment score. The result text refers to "populations". Are these defined by donor e.g. HSC-like cells from donor 116 would correspond to the "population" shown on the left-most column? How is population with signature defined? Does this correspond to high enrichment that is also statistically significant? In this case, authors could also indicate significance of enrichment on the plot for easier interpretation. Based on panel b some patients have very few cells belonging to e.g. HSC-like cells. Are these low cell number populations included to the analyses? The text mentions "Among 77 AML populations derived from thirteen patients, those features were significantly enriched in the populations that resembled HSPC, including HSC, LMPP, and GMP (21/37 vs 0/40, p< 0.0001, Fig. 3d). What is 21/37 and how is it related to the data shown in the heatmap? It could be helpful to include a separate supplementary figure that shows the data for these 77 populations, to support the patient-level summary. In addition, clarify the coloring of patient codes under the heatmap. In figS4 a similar heatmap is shown. What case numbers shown correspond to the cases (five out of eight) with LSC+HSC-like?

Fig3e: The figure description and figure are contradicting: it appears that rows correspond to cells (e.g. HSPC-like population) and genes from the signatures are shown in columns.

5) The result text paragraph starting from end of p.7 is not matching to data shown in figure 4 "eleven complete remission patients displayed a decrease in cellular diversity in response to treatment" while only three patients are shown in fig4 (three with several hundred blasts remaining). Therefore, it is hard to follow description and not possible to judge this results. In general, it would be helpful that result text refers to specific cases by their patient number to allow readers to judge the data from these respective cases. Cellular diversity is shown only from three cases shown and the mentioned degrees appears to characterize the response of P115 which corresponds to the complete remission case. Post-therapy cells of P115 are almost exclusively mapping to E/B/M-like cells (unclear what this represents, refer to comment 1) in panel d. Based on panel f pathway analysis, these cells are characterized by apoptotic pathway activation. The authors should discuss this and also comment on whether lower viability was observed when processing this sample? Moreover, showing data from the other 10 complete remission samples is needed to demonstrate their argument on decreased cellular diversity.

6) Figure 5 categorises cases as resistant (P105, 116) vs sensitive. The supplementary table indicates PR status for three cases (also 106). How is the resistant/sensitive status related to the clinical data? Overall, the figure shows 7 cases but it is not clarified why. Are these the cases with any leukemic-matched mrd cells left? Throughout the manuscript more attention should be paid in explaining why data from only certain cases are shown.

7) The fig5c result text and figure do not agree: percentage of expression is shown in figure for marker genes (legend: ""Dot plots of the normalized expression level and expression percentage of differentially expressed surface marker genes between the resistant cases and sensitive cases.", while text refers to this panels with "HSC-like clusters that abled to survive chemotherapy were dominated by CD69+ leukemic cells (named as CD69+ HSC-like subpopulation), while those with better chemo-responses after chemotherapy were enriched for CD69- HSC-like cells (Fig. 5c)". This distinction of CD69+ vs CD69- HSC-like cells appears to be a major distinction that the authors follow-up and focus on. However, it is very unclear based on data shown to understand how these two groups of cells are distinguished. Is it just the detection of CD69? Or do the CD69+ HSC-like cells separate on UMAPs from CD69- cells? The authors mention "unique features of CD69+ HSC-like cells, we first compared expression profiles between the CD69+ and CD69- HSC-like subpopulations". Where are these markers listed? Comparison of these genes that distinguish these across cases could be visualized e.g. in a heatmap.

8) Attention should be paid to wording regarding properties of CD69+ cells. The authors state in results "Upstream regulator analysis identified MTOR and STAT3 as two major suppressed hubs, which led to decreased expression of cell cycle regulators (e.g. CDK6, CCND1) as well as upregulation of CXCR4-mediated microenvironmental interaction molecules (e.g. PIM1) in CD69+ HSC-like cells (Fig. 5f)." Firstly, there is no data shown to support that mTOR and STAT3 activity is present. This could be confirmed by additional flow cytometric or CYTOF analysis of phosphorylated targets of these pathways. Secondly, that these activities lead to decreased expression of cell cycle regulators should be confirmed in cell models with CD69+ status e.g. using small molecule inhibitors that are available for both pathways. The evidence to support these arguments derives from existing literature and it is misleading to state also in the manuscript abstract "Suppression of cell cycle genes (CDK6, CCND1) and activation of adhesion regulator (PIM1) indicated a mechanistic role of CD69-mTOR axis in regulating quiescence and adhesion in this subpopulation." when the authors have not actually performed additional experiments to confirm that this indeed explains the observed expression in the two resistant cases with high CD69 expression.

9) To support that CD69 is a good biomarker for treatment resistance, the authors should evaluate how well the mRNA and surface protein levels correlate. This could be achieved in bulk using RT-qPCR and western blot/immunostaining or evaluating correspondence at single cell using e.g. Amnis flow cytometer or by microscopy combining with mRNA probes and antibody staining.

10) The authors use microarray data stratified by CD69 to support their analysis. This data corresponds to cd34+ sorted fraction. How many cells assigned to the HSC-like CD69+ category in the single cell analysis are CD34+? Or is it assumed that all HSC-like cells are CD34+?

11) The data shown in figures 5 and S5 do not reveal consistency of results between this scRNAseq analysis and bulk profiles sufficiently. It appears that supplementary tables include the DEGs but a side-by-side heatmap would be more convincing. Presently, gene names are shown only for some markers and among the indicated genes e.g. lower runx is seen in scRNA-seq heatmap while upregulated in bulk, or only 2/4 up-regulated markers shown in single cell heatmap match those marked in bulk heatmap. What is shown in panel g is not supporting very consistent trends and the authors statements should be carefully revised here. They state "CD69 was significantly up-regulated and its downstream mTOR expression signature was preferentially inhibited in two out of five relapsed patients (Patient3 and Patient5; Fig. 5g). This is not accurate as mTOR level difference is only significant in patient 5. "quiescence and CXCR4-mediated microenvironmental interaction were enhanced" Again, quiescence was significantly increased only in patient 5. Overall, the authors statement "CD69+ HSC-like cells were characterized by enhanced properties in microenvironment adhesion and cellular quiescence" requires further support.

12) Additional file 9: Table S8 expression signatures for 11 commonly observed leukemic cell types; The table provides scores but what are the gene sets used? How were they defined?

Minor comments:

- Wording : publicly large AML cohorts > large public

- It would be helpful to show CD69 expression also on the UMAP of healthy BM and projected leukemic cells

- FigS1: include NK cell marker e.g. GNLY

- The manuscript title does not mention main disease context (pediatric AML)

- Provide a table for targeted capture regions used

- Clarify in methods that CD34+ cells were sorted only from healthy BM.

- Provide further details integration analysis Seurat not described well; project leukemia-only or with healthy cells? which algorithm?

- Clusters were merged if they had highly similar transcriptomes - specify by which metric and cutoff

- add reference for list of chrY genes

**Reviewer 2**

**Are you able to assess all statistics in the manuscript, including the appropriateness of statistical tests used?** Yes, and I have assessed the statistics in my report.

**Comments to author:**

To dissect cellular heterogeneity and underlying gene pathways associated with chemotherapy response, Zhang et al. performed scRNA-Seq analysis of 13 paired pediatric AML samples before and after chemotherapy. The authors presented the following major findings: 1) leukemic stem cells (LSCs) and OXPHOS signatures are prevalent among stem/progenitor-like leukemic cells, and mutually exclusive in HSC-like and LMPP-like populations; 2) CD69+ HSC-like leukemic cells are chemo-resistant and associated with poor clinical outcome. The manuscript provides a large amount of data that could be valuable to the field. However, there are multiple major concerns that diminishes my enthusiasm for publication in this journal.

Major comments:

1. The authors used computational approaches to predict leukemic cells by integration with data generated with healthy donor samples and using mutation information metric. However, the prediction of leukemic cells is not conclusive, especially for the post-therapy samples. It would be nice to provide more evidence such as: 1) the clinical flow data and correlation with the surface markers expression of scRNA-Seq to confirm the prediction accuracy; 2) in fig. 2c and fig. S2c, the authors presented the UMAP colored by the mutation status. It is hard to tell the fractions of mutant cells in both pre-therapy and post-therapy samples. Barplot might be more informative.

2. The authors identified CD69+ HSC-like leukemic cells by comparing sensitive and resistant pre-therapy samples. It would be more informative to provide the expression levels of CD69 and other discovered signatures (Figure 5a) in the paired post-therapy samples. This could be the direct evidence that CD69+ HSC-like leukemic cells are chemo-resistance.

3. Which stage of post-therapy samples were collected? After one month chemotherapy? If so, data presented on P115, P117, P118, P124 are not complete remission since the MRD > 0.1%. This is also related to figure 5a. How were the resistant and sensitive cases defined? This needs to be stated more clearly.

4. The mutation analysis (fig 6d & fig S7d) is interesting. However, it would be more convincing if the authors could find the overlapped mutations in the cohort in this manuscript. This could be done using targeted DNA-Seq at bulk level or GoT method on the leftover barcoded cDNA of scRNA-Seq samples.

5. The authors presented a model in fig 7. However, no data/analysis in this study support the claim that CD69-S1P1 downregulates mTOR pathway/promotes CXCL4-CXCL12 interaction. Thus, this needs to be deleted or rephrased or provide more data to support the model.

6. In general, all results are based on computational analysis. There is no experimental validation on any of the results.

Minor comments:

1. On P122 and P124 have >15000 cells captured. Did the authors use cell hashing? If not, these two samples were clearly overloaded. What are the percentages of doublets for these two samples?

2. Page 10 line 27. There is no figure 5h.

3. Table S8 only contains the results of deconvolution but no gene signatures of 11 leukemic cell types. Please explain more specifically how the deconvolution was performed.

**Authors Response**

**Point-by-point responses to the reviewers’ comments:**

We thank the Reviewers for their timely and constructive suggestions of our original manuscript. In the past more than two months, we made the best efforts to add more data or provide clarifications as suggested by reviewers. We believe that the revised manuscript has been strengthened as a consequence. The additions are marked in red in the revised version. Below please find the point-by-point response to Reviewers， with the page numbers and figures mentioned refer to the new ones in the revision.

Point-by-Point response to the reviewers’ comments:

**Reviewer reports:**

**Reviewer #1:**

**Major comments:**

**1) The description of material used should be more detailed regarding the patients included to this single cell study. The Methods section describes that participants belong to a clinical trial of low- or standard-dose chemotherapy for induction. Which treatment did these cases receive? More specifics on the drugs and their dosing regime should be provided since the definitions of resistance signatures and comparison of cases by drug response are central to this study.**

**Response:** We thank for the reviewer’s comments. The detailed treatment information including the drugs and dosing regimen are now provided in the “Material and methods” section on Page 21, as previously described (clinical trial registration number ChiCTR1800015883) [1, 2]. Those data showed that low-dose (with G-CSF, LDC/G-CSF) and standard-dose chemotherapy (SDC) for remission induction had similar mutation clearance levels, treatment response and clinical outcomes. In this scRNA-seq cohort, nine patients with AML were treated with the SDC regimen, and another four children with AML received LDC/G-CSF regimen. The treatment information of each case is now provided in Additional file 4: Table S3.

Of note, although the CD69 marker and resistance-related HSC-like signatures were first discovered in our clinical trial, this finding was validated in two large public cohorts (TCGA as adult and TARGET as pediatric cohort) with standard chemotherapy. In brief, we demonstrated the prevalence of this subpopulation and its related expression signature in AML (Fig. 7, S9 and Table 2, 3). Furthermore, we provided strong evidence that the presence of CD69+ HSC-like population and related resistance signature were associated with MRD positivity, higher relapse rate, as well as worse overall outcomes in two large cohorts (Fig. 7, S9, Table 2, 3, and new Fig. S9e).

**2) The analysis of normal BM clusters in fig1A is used to guide the matching of leukemic cells later in the analysis. Overall, the analysis revealed expected cell populations. However, what is the E/B/M cluster? Later, the authors focus on HSC-, GMP- and LMMP-like populations, but there is one case where mainly all leukemic cells matched this cell cluster which is not described. In addition, it would be important to include a UMAP figure panel that shows the matching of cells to different donors since it is relevant for evaluating that technical batch effects are not having a major impact on cell clustering.**

1. **Comments:” what is the E/B/M cluster? there is one case where mainly all leukemic cells matched this cell cluster which is not described”.**

**Response:** E/B/M cluster refers to eosinophil/basophil/mast cell progenitor, which has multiple differentiation potentials to mast cell, basophil, and eosinophil lineages. It has been identified in normal hematopoiesis and diagnosed AML in previous scRNA-seq studies[3-7]. They reported that the E/M/B progenitor is transcriptionally and immunophenotypically similar to megakaryocyte–erythroid progenitor (MEP), and transcriptionally shared a common progenitor with MEP. This new cell type discovered by scRNA data currently does not have a counterpart in the standard hematopoietic models. In the analysis of our scRNA data, we could transcriptionally define an E/B/M normal cluster in all CD34-sorted samples from healthy donors. We also identified an E/B/M-like leukemia cluster in majority (8/10) of pre-therapy samples of CBF-AML and biCEBPA-mutated AML, as well as in two post-therapy samples of CBF-AML. Consistent with previous reports, E/B/M and E/B/M-like clusters highly expressed mast cell, basophil, and eosinophil canonical markers (such as HDC, CLC, MS4A2/3) and transcription factors important for early MEP commitment (GATA1/2, KLF1, Fig. S1b).

Our analysis showed that the E/B/M-like cells expressed RUNX1-RUNX1T1 fusion transcripts in the patient P115 who had achieved clinical CR for 42 months until submission. After therapy, these cells accounted for the entire tumor population. In the whole bone marrow, the residual tumor cells represented 1.6%. The clinical significance of the E/B/M-like leukemic population remains unclear.

To make it clear, we added the full names of each cluster into the figure 1 legend on Page 17. It now reads “HSC: hematopoietic stem cell. LMPP: lymphoid-primed multi-potential progenitor. GMP: granulocyte-macrophage progenitor. CLP: common lymphoid progenitor. MEP: megakaryocyte (MK) and erythroid progenitor. E/B/M: eosinophil/basophil/mast cell progenitor that expresses three lineages-specific canonical markers and MEP commitment-essential transcription factors, consistent with previous reports.”

1. **Comments:”In addition, it would be important to include a UMAP figure panel that shows the matching of cells to different donors since it is relevant for evaluating that technical batch effects are not having a major impact on cell clustering”.**

**Response:** To address this question, we have now included a UMAP projection to show the origin of different donors (new Fig. S1b). Batch effect rises from different individual cell donors, sample groups, or experiment conditions. For our data, each cluster shown in Fig. 1a merged different batches of the same cell type. Cells from each sample / donor were represented across multiple cell-type clusters. This indicated that batch effects were corrected, and had no impact on cell clustering.

**3) The authors show representative UMAP generated with healthy donor cells and leukemic samples from pre- and post-treatment (in Fig. 2) and compare leukemic cells to healthy bone marrow based on similarity to normal cell transcriptomes using scMap tool (Fig. 3). Based on the example case, leukemic cell transcriptomes are distict from their normal BM counterparts as they segregate in Fig. 2.** **How well matched are the cells based on the three correlation metrics that scmap calculates (cosine similarity and Pearson and Spearman correlations)? What percentage of cells (by sample) were matched by the criteria proposed by the tool developers (in the scmap publication "We required that at least two of the similarities be in agreement, and that at least one be >0.7 If these criteria were not met, then c was labeled as "unassigned")?** **How well were the non-leukemic cells matched in comparison to the leukemic? This further clarification is important since majority of downstream analyses are based on the distinction of HSC-, GMP and LMMP-like cells and comparing their properties across cases and** **there is always uncertainty in matching cells between experiments and** **this is more challenging when matching cells from disease to healthy reference. The current evaluation of success is based on comparison to clinical morphology (M1-M7 categories).** **The authors argue that mainly there is agreement, but do not discuss what could underlie the discrepancies observed (P114 and P118 belong to M4/M5 categories but have few monocyte-like cells matched; in contrast M2 phenotype P120 has mainly monocyte-like cells.** **Do the authors have additional (e.g. flow cytometric by CD34 vs CD14 and other stem- vs monocyte markers) data to ascertain which assignments are more accurate regarding blast phenotype?**

1. **Comments**: How well matched are the cells based on the three correlation metrics that scmap calculates (cosine similarity and Pearson and Spearman correlations)? What percentage of cells (by sample) were matched by the criteria proposed by the tool developers (in the scmap publication "We required that at least two of the similarities be in agreement, and that at least one be >0.7 If these criteria were not met, then c was labeled as "unassigned")? How well were the non-leukemic cells matched in comparison to the leukemic?

**Response**: Considering leukemia cells will partially maintain the original cellular hierarchies and express a lineage-affiliated transcriptional program similar to the normal hematopoietic cells, the similarity-based projection strategy has been commonly used to discriminate leukemic cell types/differentiation stages in previous studies[8-11]. We took advantage of the widely-used scmap tool to assign the leukemic cell types. This tool has two modes of projection, scmap-cell and scmap-cluster, that allow to project a new cell onto a reference data set to identify the most similar cell or cluster respectively. As the tool developers recommended, scmap-cluster works well for discrete cluster labels with a similarity cutoff of 0.7(the reviewer referred here), while scmap-cell is more suitable for continuous differentiation trajectories using 0.5 as the similarity cutoff. As hematopoiesis is a continue process, we used scmap-cell in our study.

Our data showed that similarity between cells and their nearest healthy neighbor are high, with median cosine similarity of 0.85 (range: 0.35-0.91, new Fig.S3c). On average, 99.03% (range: 94.89%-99.95%) of cells (by sample) met the criterial proposed by the developers (using 0.5 as cutoff in scmap-cell mode).

The cosine similarity between leukemic cells and their nearest neighbor are high, with median of 0.86 (range: 0.35-0.91, new Fig.S3d). On average, 98.23 % (range: 80%-100%) of leukemia cells (by sample) met the criterial proposed by the developers. Similarly, the normal (non-leukemic) cells exhibited a median cosine similarity of 0.78 (range: 0.32-0.92) to their nearest neighbor. On average, 98.67% (range: 94.88%-99.93%) of normal cells (by sample) met the criterial proposed by the developers.

1. **Comments: The current evaluation of success is based on comparison to clinical morphology (M1-M7 categories). The authors argue that mainly there is agreement, but do not discuss what could underlie the discrepancies observed (P114 and P118 belong to M4/M5 categories but have few monocyte-like cells matched; in contrast M2 phenotype P120 has mainly monocyte-like cells. Do the authors have additional (e.g. flow cytometric by CD34 vs CD14 and other stem- vs monocyte markers) data to ascertain which assignments are more accurate regarding blast phenotype?**

**Response:** Following the reviewer’s suggestion, we added the flow data to evaluate the consistency of stem and progenitor cell fraction between scRNA-seq and flow in these three patients (new Fig. S3e). We identified the leukemic cells (myeloblasts) as previously described and assessed the proportion of stem and progenitor cells using flow cytometry by examination of the expression of canonical stem cell markers (CD34). Flow cytometry showed that 77.92% and 95.98% leukemic cells were CD34^+^ in P114 and P118 respectively, which were consistent with transcriptionally defined leukemic cells were mainly classified as HSPC-like (99.55% in P114, and 95.13% in P118). Although these two patients belong to M4/M5 by morphology examination, both flow cytometry and scRNA-seq identified high proportion of HSPC-like populations. As for M2 phenotype P120, the situation was complicated. The high fraction of CD34+ leukemic cells by flow examination was not consistent with our scRNA-seq prediction. However, the flow cytometry indicated the neutrophil is abnormal and showed high expression of leukemia-associated marker CD56. To a certain extent, this observation supported the neutrophil-like cells as leukemia and explained HSPC-like cells of relatively low proportion in scRNA-seq. Thus, scRNA-seq data were consistent with the estimation based on the surface protein level. We have added the result on Page 7.

**4) More clarification is needed to interpret panel 3d. The color in this heatmap corresponds to enrichment score. The result text refers to "populations". Are these defined by donor e.g. HSC-like cells from donor 116 would correspond to the "population" shown on the left-most column? How is population with signature defined? Does this correspond to high enrichment that is also statistically significant? In this case, authors could also indicate significance of enrichment on the plot for easier interpretation.  Based on panel b some patients have very few cells belonging to e.g. HSC-like cells. Are these** **low cell number populations included to the analyses? The text mentions "Among 77 AML populations derived from thirteen patients, those features were significantly enriched in the populations that resembled HSPC, including HSC, LMPP, and GMP (21/37 vs 0/40, p< 0.0001, Fig. 3d). What is 21/37 and how is it related to the data shown in the heatmap? It could be helpful to include a separate supplementary figure that shows the data for these 77 populations, to support the patient-level summary. In addition, clarify the coloring of patient codes under the heatmap. In figS4 a similar heatmap is shown. What case numbers shown correspond to the cases (five out of eight) with LSC+HSC-like?**

**Fig3e: The figure description and figure are contradicting: it appears that rows correspond to cells (e.g. HSPC-like population) and genes from the signatures are shown in columns.**

**Response:** To present our data more clearly, we have improved Fig. 3d and Fig. S4c to indicate significance of enrichment and provided a new supplementary figure Fig. S4a showing the expression of signatures in all 73 (not 77) populations. In addition, we added and corrected the corresponding details in our revised manuscript.

The reviewer is right about the presentation of heatmap, each column represents a population derived from the specific patient. The population with a signature was defined if the population showed normalized enrichment score (NES) more than 1.9 and false discovery rate (FDR) less than 0.001, which were indicated with asterisk in the revised Fig. 3d. In addition, these populations with a small number of cells (<50 cells) were excluded in the gene enrichment analysis, and not shown in the Fig. 3d. Therefore, there were totally 37 HSPC-like populations (columns) in all 13 patients. Among them, 20 HSPC-like populations significantly expressed chemo-resistant signatures (indicate with an asterisk). The colors of patient codes represent the resistant (red) cases whose HSC-like population possessing LSC signature persisted after therapy, and the sensitive (blue) cases whose HSC-like population possessing LSC signature were cleared after therapy.

As for the patient-level summary of all 73 (not 77) populations, we provided a new supplementary figure Fig. S4a as suggested by the reviewer.

A similar heatmap Fig. S4b was also revised and presented as new Fig. S4c. We added asterisk to show the population possessing a signature. As shown in the Fig. S4c, eight populations with LSC signature were indicated with asterisk, including five HSC-like populations derived from AML921A, AML419A, AML328, AML1012 and AML210A, two LMPP-like populations derived from AML419A and AML1012, one GMP-like population derived from AML556.

The legend of Fig. 3e were amended for better clarification in the revised Fig. 3e legend on Page 18.

**5) The result text paragraph starting from end of p.7 is not matching to data shown in figure 4 "eleven complete remission patients displayed a decrease in cellular diversity in response to treatment" while only three patients are shown in fig4 (three with several hundred blasts remaining). Therefore, it is hard to follow description and not possible to judge this results. In general, it would be helpful that result text refers to specific cases by their patient number to allow readers to judge the data from these respective cases. Cellular diversity is shown only from three cases shown and the mentioned degrees appears to characterize the response of P115 which corresponds to the complete remission case. Post-therapy cells of P115 are almost exclusively mapping to E/B/M-like cells (unclear what this represents, refer to comment 1) in panel d. Based on panel f pathway analysis, these cells are characterized by apoptotic pathway activation. The authors should discuss this and also comment on whether lower viability was observed when processing this sample? Moreover, showing data from the other 10 complete remission samples is needed to demonstrate their argument on decreased cellular diversity.**

**Response:** Accordingly, we have now showed the comparison of cell composition between pre- and post-therapy samples from the additional 10 patients in Fig. S4e of the revised manuscript.

The E/B/M-like exhibited increasing apoptosis pathway after therapy, which were possibly induced by chemotherapy treatment. This observation was consistent with the prior in vitro and in vivo studies, which showed the Ara-C would induced DNA double strand breaks and apoptotic morphology [12]. We have added this in the revised MS on Page 9.

To assess the cell viability of this sample (post-therapy sample of P115), two methods were used: cell viability measurement before library preparation and evaluation based on the proportion of mitochondrial reads. The cell viability measurement using Acridine orange (AO) and propidium iodide (PI) staining is a standard assay to accurately distinguish live cells from dead cells and debris. It showed that this sample had 86.69% live cells. In addition, a high fraction of mitochondrial counts is indicative of cells with broken membrane, and thus, cytoplasmic mRNA has leaked out and mitochondrial mRNA is still preserved. E/B/M-like cells had low fraction of mitochondrial reads (mean: 8.06%; range: 0.47%-14.55%). These data indicated high viability of E/B/M-like cells.

**6) Figure 5 categorises cases as resistant (P105, 116) vs sensitive. The supplementary table indicates PR status for three cases (also 106). How is the resistant/sensitive status related to the clinical data? Overall, the figure shows 7 cases but it is not clarified why. Are these the cases with any leukemic-matched mrd cells left? Throughout the manuscript more attention should be paid in explaining why data from only certain cases are shown.**

**Response:** Regarding to the definition of resistant and sensitive cases, we focused on the seven cases (out of the total thirteen patients) who possessed LSC signatures in the HSC-like population. Two of the seven HSC-like populations persisted after therapy (Fig. 3d, 4a-d). Therefore, the two patients with persistent HSC-like populations were referred to as “resistant cases”. In contrast, HSC-like cell clusters in the remaining five patients became undetectable after therapy. These five cases were referred to as “sensitive cases”. We have added this explanation in the revised manuscript on Page 9.

The resistant defined in our study showed consistence with clinical response. Specifically, of three patients who achieved clinical partial remission (PR, P105, P116 and P106), two (P105 and P116) were defined as resistant. And all 10 CR patients were defined as sensitive or without possessing LSC signatures.

**7) The fig5c result text and figure do not agree: percentage of expression is shown in figure for marker genes (legend: ""Dot plots of the normalized expression level and expression percentage of differentially expressed surface marker genes between the resistant cases and sensitive cases.", while text refers to this panels with "HSC-like clusters that abled to survive chemotherapy were dominated by CD69+ leukemic cells (named as CD69+ HSC-like subpopulation), while those with better chemo-responses after chemotherapy were enriched for CD69- HSC-like cells (Fig. 5c)". This distinction of CD69+ vs CD69- HSC-like cells appears to be a major distinction that the authors follow-up and focus on. However, it is very unclear based on data shown to understand how** **these two groups of cells are distinguished. Is it just the detection of CD69? Or do the CD69+ HSC-like cells separate on UMAPs from CD69- cells? The authors mention "unique features of CD69+ HSC-like cells, we first compared expression profiles between the CD69+ and CD69- HSC-like subpopulations". Where are these markers listed? Comparison of these genes that distinguish these across cases could be visualized e.g. in a heatmap.**

1. **Comment: The fig5c result text and figure do not agree: percentage of expression is shown in figure for marker genes (legend: ""Dot plots of the normalized expression level and expression percentage of differentially expressed surface marker genes between the resistant cases and sensitive cases.", while text refers to this panels with "HSC-like clusters that abled to survive chemotherapy were dominated by CD69+ leukemic cells (named as CD69+ HSC-like subpopulation), while those with better chemo-responses after chemotherapy were enriched for CD69- HSC-like cells (Fig. 5c)".**

**Response:** We recognized that we did not provide appropriate interpretation of fig. 5c in the text of the original submission. We have now rephrased and expanded the text on Page 10: “Among several differentially expressed cell surface marker genes (*CD69*, *CD79A*, *CD317/BST2*, *RGS10* and *B2M*), *CD69,* a type II transmembrane C-type lectin receptor, exhibited the most prominent change between resistant and sensitive HSC-like cells (fold change = 1.75; Fig. 5d and Additional file 6: Table S5). Furthermore, the two resistant cases have nearly 90% of HSC-like cells expressing *CD69* (90.00% in P105 and 89.20% in P116) while less than 40% of HSC-like cells (median: 39.52%, range: 24.40%-88.97%) from the sensitive cases expressing *CD69* (Fig. 5d). This data suggested that HSC-like populations that abled to survive chemotherapy were dominated by *CD69*^+^ cells (named as *CD69*^+^ HSC-like subpopulation), while those became undetectable after chemotherapy were enriched for *CD69*^-^ HSC-like cells (named as *CD69*^-^ HSC-like subpopulation, Fig. 5d).”.

1. **Comment: This distinction of CD69+ vs CD69- HSC-like cells appears to be a major distinction that the authors follow-up and focus on. However, it is very unclear based on data shown to understand how these two groups of cells are distinguished. Is it just the detection of CD69? Or do the CD69+ HSC-like cells separate on UMAPs from CD69- cells?**

**Response:** Following the reviewer’s suggestion, we have now provided the clustering and UMAP projection of HSC-like cells from these seven patients. The UMAP projection of HSC-like populations from these seven patients showed two major clusters (Fig. S4f). Resistant HSC-like populations were clustered together and showed significantly higher expression of *CD69*, while majority (four out of five) of sensitive HSC-like populations formed another cluster with lower expression of *CD69*. Together, these data supported that *CD69*^+^ HSC cells were distinct from *CD69*^-^ HSC cells, and that those two populations largely separate from each other on UMAP.

1. **Comment: The authors mention "unique features of CD69+ HSC-like cells, we first compared expression profiles between the CD69+ and CD69- HSC-like subpopulations". Where are these markers listed? Comparison of these genes that distinguish these across cases could be visualized e.g. in a heatmap.**

**Response:** Two methods, differential expressed gene analysis and Gene Set Enrichment Analysis (GSEA), were used to explore the molecular features between *CD69*^+^ and *CD69*^-^ HSC-like subpopulations in our original submission. The result mentioned here was performed by GSEA analysis to examine the specific gene expression signatures derived from hematopoietic cells. And the differentially expressed genes had shown in our original submission in Fig. 5a and Additional file 6: Table S5. To make it clearer, we have revised and simplified this statement on Page 9.

**8) Attention should be paid to wording regarding properties of CD69+ cells. The authors state in results "Upstream regulator analysis identified MTOR and STAT3 as two major suppressed hubs, which led to decreased expression of cell cycle regulators (e.g. CDK6, CCND1) as well as upregulation of CXCR4-mediated microenvironmental interaction molecules (e.g. PIM1) in CD69+ HSC-like cells (Fig. 5f)." Firstly, there is no data shown to support that mTOR and STAT3 activity is present. This could be confirmed by additional flow cytometric or CYTOF analysis of phosphorylated targets of these pathways. Secondly, that these activities lead to decreased expression of cell cycle regulators should be confirmed in cell models with CD69+ status e.g. using small molecule inhibitors that are available for both pathways. The evidence to support these arguments derives from existing literature and it is misleading to state also in the manuscript abstract "Suppression of cell cycle genes (CDK6, CCND1) and activation of adhesion regulator (PIM1) indicated a mechanistic role of CD69-mTOR axis in regulating quiescence and adhesion in this subpopulation." when the authors have not actually performed additional experiments to confirm that this indeed explains the observed expression in the two resistant cases with high CD69 expression.**

**Response:** Thank for the reviewer’s suggestion. Combined with the review#2 comment 5, we have performed a series of experimental validation with primary AML samples and CD69-overexpressing AML cell lines. Our new data supported the hypothesis that CD69-mTOR axis regulates quiescence and adhesion associated molecules. We added new figures (Fig. 6 and Fig. S6 in the revised MS) to present these new data and rephrased the sentence in abstract.

To address the function of CD69 in regulating its downstream pathways, CD69-overexpresing AML cell lines were established (Fig. S6a). We found that *CD69* overexpression resulted in reduced levels of p-mTOR and downstream p-P70 and p-4EBP1 in both HL60 and Kasumi-1 (Fig6a). The protein level of total STAT3 or p-STAT3 was comparable in control and CD69-overexpressing HL60 or Kasumi-1 cells (Fig. S6b). In the meanwhile, CD69 overexpression dramatically decreased the expression of CDK6 and Ki67 and increased CXCR4 expression (Fig. 6 b-d). Subsequently, we analyzed the adhesive interaction of these cell lines with human mesenchymal stem cells (hMSC). Cell adhesion assay showed CD69 overexpression significantly increased adherent cells versus hMSC ratios (Fig. 6e). Since homing to bone marrow (BM) is a crucial step for AML cells to interact with stromal cells, we used Transwell assay to determinate if CD69 affects AML cells migration to CXCL12, which is expressed in bone marrow niches. CD69 overexpression increased cell migration toward high gradient of CXCL12 (fig. 6f). These data suggested that CD69 enhanced cell adhesion and homing to BM niche through CXCR4-CXCL12 interaction. In concordance with our findings in AML cell line, we found cell cycle regulators including Ki67, CCND1 and CDK6 were reduced and adhesion molecule CXCR4 was dramatically increased in CD69^high^CD34^+^CD38^-^ HSC-like populations from primary AML patients. In supportive of these findings, previous functional studies documented a critical role of CD69 in sustaining quiescent and adhesive states of normal HSPC in bone marrow through inhibition of mTOR signaling. These results suggested a functional role of CD69 in regulating quiescence and adhesion via mTOR signaling in leukemia stem cells.

Regarding to the second question, CD69 overexpression inhibited mTOR pathway activity. As mTOR is activated in normal conditions, Leucine, the most common used agonist of mTOR, need starvation of cells to eliminate the effect of other nutrients in the medium. We have tried to performed mTOR inhibition and rescue assays to understand whether mTOR activity lead to decreased expression of cell cycle regulators in cell models with CD69+ status e.g. using small molecule inhibitors. However, we did not obtain related data within a limited time.

**9) To support that CD69 is a good biomarker for treatment resistance, the authors should evaluate how well the mRNA and surface protein levels correlate. This could be achieved in bulk using RT-qPCR and western blot/immunostaining or evaluating correspondence at single cell using e.g. Amnis flow cytometer or by microscopy combining with mRNA probes and antibody staining.**

**Response:** To address this question, we sorted CD34^+^CD38^-^ HSC-like populations from frozen bone marrow cells of 5 AML patients using flow cytometry and examined mRNA level of *CD69* by Q-PCR. CD69 protein level in CD34^+^CD38^-^ HSC-like cells was measured by flow cytometry and quantified by mean fluorescence intensity (MFI). The data showed that mRNA and surface protein levels of CD69 were well correlated (R=0.89, p=0.045, Fig. S5c).

**10) The authors use microarray data stratified by CD69 to support their analysis. This data corresponds to cd34+ sorted fraction. How many cells assigned to the HSC-like CD69+ category in the single cell analysis are CD34+? Or is it assumed that all HSC-like cells are CD34+?**

**Response:** The HSC-like were similar with HSC, which exhibited high expression of *CD34* (Fig. S1b-c). Accordingly, all HSC-like cells were supposed to exhibit *CD34*^+^. Our data showed that nearly 90% (1886/2169) of HSC-like cells were *CD34*^+^ in the *CD69*^+^ category. The remaining 10% cells did not have detectable *CD34* transcripts. This may be related to the drop-out events in single cell techniques, which usually is due to extremely low mRNA input or the stochastic nature of gene expression.

**11) The data shown in figures 5 and S5 do not reveal consistency of results between this scRNAseq analysis and bulk profiles sufficiently. It appears that supplementary tables include the DEGs but a side-by-side heatmap would be more convincing. Presently, gene names are shown only for some markers and among the indicated genes e.g. lower runx is seen in scRNA-seq heatmap while upregulated in bulk, or only 2/4 up-regulated markers shown in single cell heatmap match those marked in bulk heatmap. What is shown in panel g is not supporting very consistent trends and the authors statements should be carefully revised here. They state "CD69 was significantly up-regulated and its downstream mTOR expression signature was preferentially inhibited in two out of five relapsed patients (Patient3 and Patient5; Fig. 5g). This is not accurate as mTOR level difference is only significant in patient 5. "quiescence and CXCR4-mediated microenvironmental interaction were enhanced" Again, quiescence was significantly increased only in patient 5. Overall, the authors statement "CD69+ HSC-like cells were characterized by enhanced properties in microenvironment adhesion and cellular quiescence" requires further support.**

**Response:** We thank the reviewer for pointing out that DEGs identified in our scRNA analysis did not show strong consistency with those DEGs derived from public bulk RNA profiles. To address this concern, we performed additional analyses at both gene and biological function levels. In summary, two CD69-related signatures identified in different datasets had a general agreement in the biological functions.

At the gene level, a large proportion of DEGs identified in one dataset was not identified as differently expressed in the other assay. However, we could observe that about 40% of scRNA DEGs had a similar trend, although statistically marginal, in the change of their expression (over 1.05 fold difference) in bulk RNA dataset (new Fig. S5g). Specifically, 45% of downregulated scRNA DEGs exhibited a reduction in bulk RNA expression, and 28% of upregulated scRNA DEGs showed an increased expression in bulk RNA dataset. We note that differences in gene expression observed in scRNA analysis did not reach statistical significance in bulk RNA. This may presumably resulted from the intrinsic technical limitations of two different platforms employed, as reported in other studies[13-16] , such as high sparsity of scRNA-seq data, limited and variable sensitivity and average signals of detection for bulk microarrays. In addition, differences in age group (pediatric vs. adult), patient composition, cell population (immunophenotypically sorted CD34^+^CD38^-^ cells vs transcriptionally defined HSC-like cells) between these two datasets, may contribute to observed results as well.

At the biological function level, we included additional analyses to examine the degree of consistency of our finding between these two datasets. These new analyses showed that a similar set of biological function terms between our scRNA and public bulk profiles, such as cell adhesion and quiescence, were associated with resistant *CD69*^+^ HSC-like cells. Specifically, scRNA DEGs were mainly involved in repression of proliferation of stem cells, cellular movement, and cell death (Fig. 5b and S5a), and bulk RNA DEGs were related to activation of cell adhesion, chemotaxis and viability (new Fig. S5h), as revealed by IPA biological function analysis. Consistently, as shown in our original manuscript, known expression signatures including cell adhesion, quiescence, proliferation, and migration exhibited the enrichment/distribution bias between two conditions in both datasets (Fig. 5c and S5i). Therefore, both different datasets consistently support that *CD69*^+^ HSC-like cells were associated with activation of cell adhesion and quiescence, and inhibition of cellular movement and active cycling. Now we also have added this result in our revised manuscript on Page 10

Regarding to the second question about Fig. 5g, we thank the reviewer for the comments. We recognized that scRNA-seq data obtained in our reanalysis of Stetson LC. et al. dataset [17] was not sufficient to support the claim. We have removed this panel and its corresponding text in the revised manuscript.

Following the reviewer’s critique, we have performed a series of functional experiments to vigorously determine the role of CD69 in regulating cell adhesion and quiescence in primary AML patients and human AML cell lines. Specifically, experimental data from CD69 overexpressing AML cell lines showed that CD69 promoted cell quiescence, adhesion and migration toward CXCL12. This were further validated by the detection of quiescence and adhesion molecules. Overexpression of CD69 in AML cell lines result in reduction of the phosphorylation of mTOR and its downstream substrates (p-P70S6, p-4EBP1), cell cycle regulator CDK6 and upregulation of chemokine receptor CXCR4. Consistently, this were also observed in primary CD69^+^ HSC-like cells sorted from AML patients. Together, these data indicated that CD69 repressed mTOR signaling and promoted CXCR4-CXCL12 interaction and quiescence, which was consist with our scRNA-seq data of AML patients. Combined with the major comment Q8, major comment Q5 and Q6 raised by reviewer #2, we added three new figures (Fig. 6 and Fig. S6-7 in the revised MS) to present these data.

**12) Additional file 9: Table S8 expression signatures for 11 commonly observed leukemic cell types; The table provides scores but what are the gene sets used? How were they defined?**

**Response:** We have now provided the list of signature genes (Additional File 9, Table S8) and their average expression profiles for 11 leukemic cell types (Fig. S8a). Furthermore, we also precisely described the method for identifying signature genes on Page 31.

**Minor comments:**

**-(1) Wording: publicly large AML cohorts > large public**

**Response:** We have changed the words into “large public AML cohorts” in Page 20.

**-(2) It would be helpful to show CD69 expression also on the UMAP of healthy BM and projected leukemic cells**

**Response:** We thank for the reviewer’s comments, and have now showed *CD69* expression in healthy hematopoietic cells and in projected leukemic cells (new Fig. S5d).

In normal hematopoiesis, *CD69* expression was correlated with cell types along the HSC to lymphoid differentiation axis. Specifically, *CD69* was expressed actively in mature lymphoid cells, and weakly in HSC, LMPP, and CLP. This was in agreement with data in previous publications[18, 19]. In addition, each of these cell types showed heterogeneous levels of *CD69*, with a portion not expressed. We observed a similar expression pattern in normal hematopoietic cells that were defined in leukemia patients. Interestingly, the level of *CD69* showed a significant increase in the E/B/M cells from two AML patients (P116, P122).

In contrast, the projected leukemic cells expressed *CD69* more highly than all normal cells. Furthermore, the expression of *CD69* varied markedly between tumors and between leukemic populations. Primitive HSC-like and progenitor-like populations exhibited the most prominent expression of *CD69*, especially for two resistant patients. Interestingly, differentiated leukemic cells did not express *CD69*. These data were consistent with the notion that CD69-highly expressed AML cells were capable of self-renewal[20].

**-(3) FigS1: include NK cell marker e.g. GNLY**

**Response:** NK cell markers such as *GNLY*, *GZMB*, and *NKG7* were shown in Fig. S1c. To represent the data more clearly, we have moved all the gene names onto the left side of the heatmap (Fig. S1b), and visualized *NKG7* expression in a new UMAP figure (Fig. S1c).

**-(4) The manuscript title does not mention main disease context (pediatric AML)**

**Response:** We have revised the title, which now reads, “Single-cell Transcriptomics Reveals Multiple Chemo-Resistant Properties in Leukemic Stem and Progenitor Cells in Pediatric AML”.

**-(5) Provide a table for targeted capture regions used**

**Response:** We have now provided the list of our captured genes and also mentioned in the “Material and methods” section on Page 22, which now reads, “The targeted capture region for all exons of the 1025 genes that were provided in Additional file 4: Table S3”.

**-(6) Clarify in methods that CD34+ cells were sorted only from healthy BM.**

**Response:** We added this information in the “Material and Methods” section in Page 22. It now reads “To enrich stem and progenitor cells from fresh PB or BM healthy samples, CD34+ cells were sorted using antiCD34 (Miltenyi,130-046-702)”.

**-(7) Provide further details integration analysis Seurat not described well; project leukemia-only or with healthy cells? which algorithm?**

**Response:** We have now added the details of the integration analysis in the text in “Single cell RNA-seq data analysis” under “Material and methods” on Page 24. It now reads “…different sequencing platform and experimental process. Specifically, the “FindIntegrationAnchors” was firstly used to identify 3000 anchors with canonical correlation analysis (CCA) dimensional reduction. These anchors were then used to integrate the datasets together using “IntegrateData” function with default parameters. Then an ‘integrated’ data assay was created…”

All the cells from paired pre- and post-therapy samples of the same patient were integrated with healthy donors. To make it more clearly, we have improved the statement in “Identification of malignant AML cells” under “Material and methods” on Page 25. It now reads “…All the cells from paired pre- and post-therapy samples of the same patient together with that of healthy donors were integrated and clustered, using methods as described in the “Single cell RNA-seq data analysis” section….”

**-(8) Clusters were merged if they had highly similar transcriptomes - specify by which metric and cutoff**

**Response:** We have added the details of the cluster merging analysis in “Cell type annotation of normal hematopoiesis map” under “Material and methods” on Page 24. It now reads “We calculated the pairwise Pearson’s correlations among average expression profiles of different clusters, ordered by hierarchical clustering and identified 20 groups using “corrplot” function. Clusters were merged if they belong to same group in hierarchical clustering, and further revised and inferred by the expression of known cell type-specific genes.”

**-(9) add reference for list of chrY genes**

**Response:** We have now provided the list of chromosome Y-located genes and related reference, and also described the method more clearly in the Methods section on Page 26. It now reads, “In addition, the chromosome Y deletion was determined by the expression pattern of chromosome Y-located genes. Specifically, we examined the expression of all Y chromosome located-genes[21] that have no homologs on any other chromosomes in scRNA-seq data of healthy donors. Among them, *RPS4Y1* was only one highly and pervasively expressed gene in male while not expressed in female, which made it as a good indicator of the presence of chromosome Y.”

**Reviewer #2:**

**Major comments:**

**1.    The authors used computational approaches to predict leukemic cells by integration with data generated with healthy donor samples and using mutation information metric. However, the prediction of leukemic cells is not conclusive, especially for the post-therapy samples. It would be nice to provide more evidence such as: 1) the clinical flow data and correlation with the surface markers expression of scRNA-Seq to confirm the prediction accuracy; 2) in fig. 2c and fig. S2c, the authors presented the UMAP colored by the mutation status. It is hard to tell** **the fractions of mutant cells in both pre-therapy and post-therapy samples. Barplot might be more informative.**

**Response:** Following the reviewer’s suggestion, we have added the clinical flow data as a new independent method to further support our prediction. Flow cytometry was used to identify leukemia cells with leukemia associated immunophenotype (LAIP) as previously described [22]. Eleven out thirteen patients had suitable LAIP for defining and monitoring leukemia cells at pre- and post-therapy (List in Additional file 4: Table S3), which allowed for following validation analysis. The fraction of transcriptionally defined AML cells in each sample was highly correlated with leukemia cell fraction by flow cytometry (Additional File1, new Fig. S2e). To further confirm the prediction accuracy, cells co-expressing representative LAIP (about 4-5 markers highlighted in Additional file 4: Table S3) were identified in scRNA-seq. We observed that 94.73% of those cells were classified as our transcriptionally defined leukemia cells, while only 5.27% were classified as normal cells (Fig. S2c). Furthermore, the cells co-expressing representative LAIP were detected in eleven pre-therapy samples, with a median of 14.32% (range: 4.54%-40.53%) in the predicted pre-therapy leukemia cells (new Fig. S2g). We also observed that the cells co-expressing representative LAIP in seven post-therapy samples with a median of 13.33% (range: 2.50%-33.33%) the predicted post-therapy leukemia cells. We also noticed that a large fraction of predicted leukemia cells didn’t co-express the representative LAIP, which might relate to low efficiency of scRNA-seq to capture co-expression of multiple genes.

Regarding to the second question, we now provided two bar plots to show the fraction of mutant cells in transcriptional defined leukemia cells in both pre-therapy and post-therapy samples (new Fig. S2g). The mutant cells were detectable in all 13 pre-therapy samples, with a median of 2.29% (range: 0.14%-17.47%) in leukemia cells. The mutant cells were also detected in post-therapy samples from three patients (P105, P115 and P116) with sufficient cell numbers with a median of 4.20% (range: 2.86%-14.99%) in leukemic cells. No mutant cells detected in the remaining 10 post-therapy samples, which might relate to the very low fraction of leukemia cells in those samples and limited cell numbers captured by 10x. In addition, the fraction of mutant cell was consistent with previous finding using 3’ scRNA-seq, where they showed a median of 3.00% (range:0.29%-30.43%) mutant cells of pre-therapy leukemia cells and 1.98% (only in 2/14 samples: 1.86% and 2.10%) mutant cells of post-therapy leukemia cells (new Fig. S2h) [8]. This low fraction of mutant cells related to variant allele frequency, the mutation site far away from the 3’ end of the transcript/3’-biased transcript coverage and allelic dropout inherent to 3’ platforms[8, 11].

In summary, we showed the consistency of leukemia cell fraction between scRNA and flow, the expression of LAIP and the fraction of mutant cells in transcriptional predicted leukemia cells from pre- and post-therapy samples. These new data now have been incorporated into the revised manuscript on Page 6-7.

**2.    The authors identified CD69+ HSC-like leukemic cells by comparing sensitive and resistant pre-therapy samples. It would be more informative to provide the expression levels of CD69 and other discovered signatures (Figure 5a) in the paired post-therapy samples. This could be the direct evidence that CD69+ HSC-like leukemic cells are chemo-resistance.**

**Response:** We focused on the seven patients (out of the total thirteen patients) who possessed LSC signatures in the HSC-like population. Two of the seven HSC-like populations persisted after therapy, which were referred to as “resistant cases” (Fig. 3c). In contrast, HSC-like cell clusters in the remaining five patients became undetectable after therapy. Therefore, we provided a new figure to visualize the expression levels of *CD69* and our discovered signatures in paired pre- and post-therapy HSC-like cells of those two resistant cases (P105 and P116, new Fig. S5b). For the patient P105, post-therapy HSC-like cells maintained the *CD69*-included expression signature. In addition, the patient P116 kept the expression levels of about 75% of genes including *CD69* after therapy. Interestingly, several key genes such as *CDK6* and *ANXA1/2* involving in cell cycle and migration were further reduced in P116. This new data showed that *CD69* and related expression signature was still maintained after treatment, which indicates that this signature related to chemo-resistance. This result has also been incorporated on Page 10, which now reads “The expression of CD69 were still maintained in post-therapy HSC-like populations of the two resistant patients (Fig. S5b).”

**3. Which stage of post-therapy samples were collected? After one month chemotherapy? If so, data presented on P115, P117, P118, P124 are not complete remission since the MRD > 0.1%. This is also related to figure 5a. How were the resistant and sensitive cases defined? This needs to be stated more clearly.**

**Response:** The post-therapy samples were collected at the end of the first cycle induction chemotherapy, which are about 26 days of chemotherapy course. Complete remission (CR) was defined as ＜5% bone marrow blasts and further classified as CR with MRD positivity or negativity according to the MRD levels. Accordingly, these four patients showed bone marrow blast < 5% and MRD > 0.1%, therefore they achieved CR with MRD positivity. This was revised in the Additional file 4: Table S3. We also added the text in “Clinical samples” under “Material and Methods” on Page 21 to indicate the exact time of sample collections and response criteria definitions.

Regarding to the definition of resistant and sensitive cases, we focused on the seven cases who possessed LSC signatures in the HSC-like population. Two of the seven HSC-like populations persisted after therapy (Fig. 3d, 4a-d). Therefore, the two patients with persistent HSC-like populations were referred to as “resistant cases”. In contrast, HSC-like cell clusters in the remaining five patients became undetectable after therapy. These five cases were referred to as “sensitive cases”.

**4.    The mutation analysis (fig 6d & fig S7d) is interesting. However, it would be more convincing if the authors could find the overlapped mutations in the cohort in this manuscript. This could be done using targeted DNA-Seq at bulk level or GoT method on the leftover barcoded cDNA of scRNA-Seq samples.**

**Response:** Following the reviewer’s suggestion, we examined whether the mutated genes found in TCGA and TARGET cohorts (Fig. 7d and Fig. S9d) were overlapped with those in AML patients from our cohort. In the TCGA cohort, mutations of *PTPN11* were specifically enriched in the *CD69*^+^-high group (3/34 vs. 0/45, p=0.076, Fig. 7d). For our cohort, *PTPN11* alteration was only present in the resistant patients but not in those sensitive cases (1/2 vs 0/11, p=0.154). *CBFB*-*MYH11* fusion was completely mapped onto *CD69*^+^-low patients in two public cohorts. Interestingly, this fusion was exclusively occurred in the sensitive cases in our cohort (0/2 vs 3/11). This new data has been shown in Fig. S9d and stated in the main text on Page 13. It now reads, “Notably, in our scRNA-seq cohort, *PTPN11* mutation was only present in the resistant patient, while *CBFB-MYH11* fusions were exclusively occurred in the sensitive cases (Fig. S9d)”.

**5. The authors presented a model in fig 7. However, no data/analysis in this study support the claim that CD69-S1P1 downregulates mTOR pathway/promotes CXCL4-CXCL12 interaction. Thus, this needs to be deleted or rephrased or provide more data to support the model.**

**Response:** Thank for the reviewer’s suggestion. We have performed a series of functional experiments to supported that CD69 repressed mTOR signaling and promoted CXCR4-CXCL12 interaction and quiescence in primary AML patients and human AML cell lines. Specifically, experimental data from CD69 overexpressing AML cell lines showed that CD69 promoted cell quiescence, adhesion and migration toward CXCL12. This were further validated by the detection of quiescence and adhesion molecules. Overexpression of CD69 in AML cell lines result in reduction of the phosphorylation of mTOR and its downstream substrates (p-P70S6, p-4EBP1), cell cycle regulator CDK6 and upregulation of chemokine receptor CXCR4. Consistently, this were also observed in primary CD69^+^ HSC-like cells sorted from AML patients. Together, these data indicated that CD69 repressed mTOR signaling and promoted CXCR4-CXCL12 interaction and quiescence, which was consist with our scRNA-seq data of AML patients. Combined with the responses to major comment Q6, and Q8 raised by reviewer #1, we added three new figures (Fig. 6 and Fig. S6-7 in the revised MS) to present these data.

In addition, we noticed that we have no data to shown the role of S1P1 in CD69 regulation. Therefore, we have rephrased in the revised MS and revised the working model.

**6. In general, all results are based on computational analysis. There is no experimental validation on any of the results.**

**Response:** Thanks for the kindly consideration. We performed a series of functional experiments to support our analysis, including (1) Using adhesion assay, Transwell assay and examination of proliferation marker in CD69-overexpressing AML cell line, we showed that CD69 promoted cell quiescence, adhesion and migration toward CXCL12; (2) Using CD69-overexpressing AML cell lines, we validated the role of CD69-mTOR axis in regulating quiescence and adhesion molecules. This were also observed in primary CD69^+^ HSC-like cells sorted from AML patients; (3) Using RT-qPCR and flow cytometry, we showed that the mRNA and surface protein levels of CD69 were well correlated in primary AML samples, which supported CD69 as a potential biomarker; (4) We added flow cytometry data to demonstrate the accuracy of leukemic/normal prediction and cell type assignment.

Combined with the responses to Q1, Q5 and Reviewer #1 Q3, Q8 and Q9, we added data into the revised manuscript on Page 6,7,9-11.

**Minor comments:**

1.    On P122 and P124 have >15000 cells captured. Did the authors use cell hashing? If not, these two samples were clearly overloaded. What are the percentages of doublets for these two samples?

**Response:** We performed 10 X genomics scRNA-seq on each sample without using cell hashing. Technically, 10x genomics captures 100-20,000+ cells per sample without cell hashing. Published studies showed that it is common to obtain over 15,000 high-quality cells per sample, such as averagely 17,000 cells (range: 11,620 - 21,731) by Allegra A. Petti, et al., 2019 [11]and more than 15,000 cells in five samples by Xianwen Ren, et al. 2021[23]. Moreover, cell viability assessment before library construction showed that both post-therapy samples had low doublet rates, with 0.94% for P122 and 0.51% for P124. We also validated their low doublet rates using Scrublet[24], an established computational approach to identify the potential doublets and/or multiplets. Together, these indicated that both samples with high cell number had low doublet artifacts and single cell high-quality transcriptomes.

**2.    Page 10 line 27. There is no figure 5h.**

**Response:** We have removed Fig. 5g (mislabeling as Fig. 5h) and the related text.

**3.    Table S8 only contains the results of deconvolution but no gene signatures of 11 leukemic cell types. Please explain more specifically how the deconvolution was performed.**

**Response:** We have now provided the list of signature genes (Additional file 9: Table S8) and represented their differential average expression profiles in 11 leukemic cell types (Fig. S9a). We have also modified the text about deconvolution analyses in the revised manuscript methods to clarify our approach on Page 31. It now reads, “The EPIC algorithm was used to infer the fractions of 11 leukemic cell types in pre-therapy bulk RNA samples. EPIC (version 1.1.5), a widely used deconvolution tool, was obtained from <https://github.com/GfellerLab/EPIC>, and run with default parameters according to the user manual. …The FPKM (Fragments Per Kilobase of transcript per Million mapped reads) normalized bulk expression data and the reference matrix of our 231-gene signature were used as the inputs.”

Reference:

1.	Hu Y, Chen A, Gao L, He H, Hu S: **Minimally myelosuppressive regimen for remission induction in pediatric AML: long-term results of an observational study.** *Blood Advances* 2021, **5:**1837-1847.

2.	Hu Y, Chen A, Zheng X, Lu J, He H, Yang J, Zhang Y, Sui P, Yang J, He F, et al: **Ecological principle meets cancer treatment: treating children with acute myeloid leukemia with low-dose chemotherapy.** *Natl Sci Rev* 2019, **6:**469-479.

3.	Velten L, Haas SF, Raffel S, Blaszkiewicz S, Islam S, Hennig BP, Hirche C, Lutz C, Buss EC, Nowak D: **Human haematopoietic stem cell lineage commitment is a continuous process.** *Nature Cell Biology* 2017, **19:**271-281.

4.	Roy A, Wang G, Iskander D, O'Byrne S, Thongjuea S: **Transitions in lineage specification and gene regulatory networks in hematopoietic stem/progenitor cells over human development.** *Cell Reports* 2021, **36:**109698.

5.	Zheng S, Papalexi E, Butler A, Stephenson W, Satija R: **Molecular transitions in early progenitors during human cord blood hematopoiesis.** *Mol Syst Biol* 2018, **14:**e8041.

6.	Qin P, Pang Y, Hou W, Fu R, Zhang Y, Wang X, Meng G, Liu Q, Zhu X, Hong N, et al: **Integrated decoding hematopoiesis and leukemogenesis using single-cell sequencing and its medical implication.** *Cell Discov* 2021, **7:**2.

7.	Velasco-Hernandez T, Trincado JL, Vinyoles M, Closa A, Gutiérrez-Agüera F, Molina O, Rodríguez-Cortez VC, Petazzi P, Beneyto-Calabuig S, Velten L, et al: **A comprehensive single-cell expression atlas of human AML leukemia-initiating cells unravels the contribution of HIF pathway and its therapeutic potential.** *bioRxiv* 2022**:**2022.2003.2002.482638.

8.	van Galen P, Hovestadt V, Wadsworth Ii MH, Hughes TK, Griffin GK, Battaglia S, Verga JA, Stephansky J, Pastika TJ, Lombardi Story J, et al: **Single-Cell RNA-Seq Reveals AML Hierarchies Relevant to Disease Progression and Immunity.** *Cell* 2019, **176:**1265-1281.e1224.

9.	Granja JM, Klemm S, McGinnis LM, Kathiria AS, Mezger A, Corces MR, Parks B, Gars E, Liedtke M, Zheng GXY, et al: **Single-cell multiomic analysis identifies regulatory programs in mixed-phenotype acute leukemia.** *Nat Biotechnol* 2019, **37:**1458-1465.

10.	**Elucidating minimal residual disease of paediatric B-cell acute lymphoblastic leukaemia by single-cell analysis.** *Nature Cell Biology* 2022, **24:**242-252.

11.	Petti AA, Williams SR, Miller CA, Fiddes IT, Srivatsan SN, Chen DY, Fronick CC, Fulton RS, Church DM, Ley TJ: **A general approach for detecting expressed mutations in AML cells using single cell RNA-sequencing.** *Nat Commun* 2019, **10:**3660.

12.	Mesner PW, Jr., Budihardjo, II, Kaufmann SH: **Chemotherapy-induced apoptosis.** *Adv Pharmacol* 1997, **41:**461-499.

13.	Qiu P: **Embracing the dropouts in single-cell RNA-seq analysis.** *Nat Commun* 2020, **11:**1169.

14.	Hicks SC, Townes FW, Teng M, Irizarry RA: **Missing data and technical variability in single-cell RNA-sequencing experiments.** *Biostatistics* 2018, **19:**562-578.

15.	Lun AT, Bach K, Marioni JC: **Pooling across cells to normalize single-cell RNA sequencing data with many zero counts.** *Genome Biol* 2016, **17:**75.

16.	Canales RD, Luo Y, Willey JC, Austermiller B, Barbacioru CC, Boysen C, Hunkapiller K, Jensen RV, Knight CR, Lee KY, et al: **Evaluation of DNA microarray results with quantitative gene expression platforms.** *Nat Biotechnol* 2006, **24:**1115-1122.

17.	Stetson LC, Balasubramanian D, Ribeiro SP, Stefan T, Gupta K, Xu X, Fourati S, Roe A, Jackson Z, Schauner R, et al: **Single cell RNA sequencing of AML initiating cells reveals RNA-based evolution during disease progression.** *Leukemia* 2021, **35:**2799-2812.

18.	Lauzurica P, Sancho D, Torres M, Albella B, Marazuela M, Merino T, Bueren JA, Martínez AC, Sánchez-Madrid F: **Phenotypic and functional characteristics of hematopoietic cell lineages in CD69-deficient mice.** *Blood* 2000, **95:**2312-2320.

19.	Cibrián D, Sánchez-Madrid F: **CD69: from activation marker to metabolic gatekeeper.** *Eur J Immunol* 2017, **47:**946-953.

20.	Sachs K, Sarver AL, Noble-Orcutt KE, LaRue RS, Antony ML, Chang D, Lee Y, Navis CM, Hillesheim AL, Nykaza IR, et al: **Single-Cell Gene Expression Analyses Reveal Distinct Self-Renewing and Proliferating Subsets in the Leukemia Stem Cell Compartment in Acute Myeloid Leukemia.** *Cancer Res* 2020, **80:**458-470.

21.	Quintana-Murci L, Fellous M: **The Human Y Chromosome: The Biological Role of a “Functional Wasteland”.** *Journal of Biomedicine and Biotechnology* 2001, **1:**18-24.

22.	Schuurhuis GJ, Heuser M, Freeman S, Béné MC, Buccisano F, Cloos J, Grimwade D, Haferlach T, Hills RK, Hourigan CS, et al: **Minimal/measurable residual disease in AML: a consensus document from the European LeukemiaNet MRD Working Party.** *Blood* 2018, **131:**1275-1291.

23.	Ren X, Wen W, Fan X, Hou W, Su B, Cai P, Li J, Liu Y, Tang F, Zhang F, et al: **COVID-19 immune features revealed by a large-scale single-cell transcriptome atlas.** *Cell* 2021, **184:**1895-1913.e1819.

24.	Wolock SL, Lopez R, Klein AM: **Scrublet: Computational Identification of Cell Doublets in Single-Cell Transcriptomic Data.** *Cell Syst* 2019, **8:**281-291.e289.

**Second round of review**

**Reviewer 1**

The new experimental data included, unfortunately is not fully addressing the criticism on lack of functional validation. My main concern is that interpretations from provided experiments are not fully matching the data shown.

1) Cell line experiment with CD69 overexpression: The Western blots shown in new Fig.6a compare two AML cell lines with and without CD69 overexpression. In each blot the p-mTOR signal is lower with overexpression and authors conclude this to support the putative mechanism. However, similar decline is visible for total mTOR, and for downstream targets (total level and phosphorylated levels both affected). The difference is not reflected in loading control GAPDH, however the concomitant changes in phosphorylated and total protein amounts make the overall conclusion very questionable. (also overloading/smear issues in blots shown)

More quantitative analysis (such as fold change over total protein) would be needed to support conclusions.

2) The related primary patient data validation is shown in Fig. S7. The authors select the three highest and three lowest samples based on CD69 protein expression (panel a) for the comparisons shown in following panels. This choice appears quite arbitrary. Moreover, only 1 / 3 evaluated cell cycle markers was statistically significantly decreased in CD69+ cases by flow cytometric analyses shown. Yet, authors misleadingly write "In concordance with our findings in AML cell line, we found cell cycle regulators including Ki67, CCND1 and CDK6 were reduced .. in CD69highCD34+CD38- HSC-like populations from primary AML patients (Fig. S7a-c)." The three proteins are also depicted in summary figure 8 as "decreased" although this was not supported by collected experimental data.

3) The authors have improved the text and figures, addressing majority of my previous concerns regarding clarity. In part, it is still a struggle to follow what is written vs shown in figures:

- The data from malignant cell fraction by mutation or LAIP gene expression (figS2g) is provided to compare to the prediction. However, as it shown separately as summarized percentages (that also are not correlated to each other), it is very difficult to judge the matching to cell assignment.

- The case numbering is not consistently used to point the reader to the respective result panels. It takes a long time to read text, explore the figure and try to follow the rationale for subsets of data shown. This especially complicates interpretations related to figs 3 and 4 where further sub-populations (e.g. HSC-like that remain or disappear during treatment) are discussed. It would be important to pay attention to labelling the figure panels with additional clarifying text (dataset, or subset) and include the patient identifiers belonging to each subset at first mention in text to improve clarity across the paper.

- Unclear what is shown in figS2d and fig 3e data shown not matching text

- There are also language issues (grammar) that should be addressed to improve clarity of text

Given that this study has focused on the more rare pediatric AML, the differences between pediatric vs adult AML are surprisingly very little discussed throughout the study. As similar work characterizing post-treatment adult AML is most likely focus in several ongoing and future studies, this aspect could be the more unique feature of the present work.

**Reviewer 2**

The authors have addressed all my comments.

**Authors Response**

**Point-by-point responses to the reviewers’ comments:**

**We thank the Reviewers for their timely and constructive suggestions of our revised manuscript. In the past one month, we made the best efforts to address the new concerns as suggested by the reviewer #1. We believe that the revised manuscript has fully addressed these concerns and been strengthened as a consequence. The modified text and additions are marked in red in the revised version. Below please find the point-by-point response to Reviewers, with the page numbers and figures mentioned refer to the new ones in the latest revision.**

**Reviewer reports:**

Reviewer #1: The new experimental data included, unfortunately is not fully addressing the criticism on lack of functional validation. My main concern is that interpretations from provided experiments are not fully matching the data shown.

1) Cell line experiment with CD69 overexpression: The Western blots shown in new Fig.6a compare two AML cell lines with and without CD69 overexpression. In each blot the p-mTOR signal is lower with overexpression and authors conclude this to support the putative mechanism. However, similar decline is visible for total mTOR, and for downstream targets (total level and phosphorylated levels both affected). The difference is not reflected in loading control GAPDH, however the concomitant changes in phosphorylated and total protein amounts make the overall conclusion very questionable. (also overloading/smear issues in blots shown)

More quantitative analysis (such as fold change over total protein) would be needed to support conclusions.

**Response:** Following the reviewer’s suggestion, we repeated western blot analyses in HL60 and Kasumi-1 cells three times. The representative western blot data was shown in new Fig.6a (left) and relative quantification data, using data from three biological replicates, was shown in new Fig.6a (right).

The new results showed that *CD69* overexpression resulted in the reduction in phosphorylated levels of mTOR and its key downstream effectors (P70S6K and 4EBP1), as well as the decrease in total protein levels of mTOR and P70S6K. To examine whether phosphorylation of mTOR and its downstream effectors were inhibited, we normalized the levels of phosphorylated protein to the corresponding total protein. The relative levels of phosphorylation of mTOR, P70S6K, and 4EBP1, shown as fold change over total protein (new Fig.6a, also see right), were significantly decreased in *CD69*-overexpressing cell lines compared with those in controls. These results were consistent with previous findings that CD69 suppressed both the total and phosphorylated protein levels of mTOR and its downstream effectors in HSPCs [2018, leukemia].

The results support our conclusions that overexpression of *CD69* resulted in suppression of the mTOR signaling pathway. We revised the text, methods, and the legend of new Fig. 6a on pages 11, 31, and 21, respectively.

The revised text on page 11: ” We found that *CD69* overexpression resulted in reduced phosphorylated protein levels of mTOR and its key downstream effectors (P70S6K and 4EBP1), as well as decreased total protein levels of mTOR and P70S6K in both HL60 and Kasumi-1 cells (Fig. 6a). The relative levels of phosphorylation of mTOR, P70S6K and 4EBP1, shown as the fold change in the levels of phosphorylated protein over total protein levels, were significantly lower in *CD69*-overexpressing cell lines than those in controls.”.

The revised Methods on page 31: “To examine whether the phosphorylated protein levels of mTOR and its downstream effectors (P70S6K and 4EBP1) were significantly inhibited, we normalized the levels of phosphorylated protein to the corresponding total protein.”

The revised legend of new Fig.6a on page 21: “(Left) Western blot showing total and phosphorylated protein levels of mTOR, P70S6K, and 4EBP1 in negative control (NC) and *CD69*-overexpressing HL60 and Kasumi-1 cells. (Right) Bar plots displaying relative quantification by densitometry.”

Reference: Notario L, Alari-Pahissa E, Albentosa A, Leiva M, Sabio G, Lauzurica P. Anti-CD69 therapy induces rapid mobilization and high proliferation of HSPCs through S1P and mTOR. Leukemia. 2018;32(6):1445-1457. doi:10.1038/s41375-018-0052-x.

2) The related primary patient data validation is shown in Fig. S7. The authors select the three highest and three lowest samples based on CD69 protein expression (panel a) for the comparisons shown in following panels. This choice appears quite arbitrary. Moreover, only 1 / 3 evaluated cell cycle markers was statistically significantly decreased in CD69+ cases by flow cytometric analyses shown. Yet, authors misleadingly write "In concordance with our findings in AML cell line, we found cell cycle regulators including Ki67, CCND1 and CDK6 were reduced .. in CD69highCD34+CD38- HSC-like populations from primary AML patients (Fig. S7a-c)." The three proteins are also depicted in summary figure 8 as "decreased" although this was not supported by collected experimental data.

**Response:** To clarify the selection strategy, we added the following description to the “Methods” on page 32 and revised the figure legend of Fig. S7 in Additional file 1.

The revised Methods on page 32: “We collected data from 21 patients, out of which 19 were successfully evaluated with all candidate molecules and included in subsequent analysis. To investigate the potential relationship between CD69 expression and adhesion/proliferation-related molecules, we adopted a previously described top and bottom 20% grouping method [78, 79] to select CD34^+^CD38^-^ samples for subsequent analyses. Specifically, CD34^+^CD38^-^ samples with CD69 MFI ranking in the top 20% in an ascending order were classified as the "CD69^high^" group (3 out of 19), while those with CD69 MFI ranking in the bottom 20% in an ascending order were classified as the "CD69^low^" group (3 out of 19). The remaining samples were classified as the “CD69^middle^” group (13 out of 19).”

The revised figure legend of Fig. S7a in Additional file 1: “Histogram shows the surface protein levels of CD69 in CD34^+^CD38^-^ (HSC-like) cell populations sorted from pre-therapy BM samples of AML patients (n=19), ranked from lowest to highest. Cell populations with CD69 MFI ranking in the top 20% and bottom 20% were classified as the "CD69^high^" group (n=3) and the "CD69^low^" group (n=3), respectively. The remaining cell populations were classified as the “CD69^middle^" group (n=13).”

Importantly, when data from all 19 patients were analyzed using additional grouping methods (top 20% vs bottom 80%), the results (as shown below) were consistent with those presented in Fig. S7b,c. These data support that the classic proliferation marker Ki67 was significantly reduced in CD69^high^CD34^+^CD38^-^ HSC-like populations from primary AML patients.

We also thank the reviewer for correctly pointing out the inaccurate description of CCND1 and CDK6 flow data in the CD69^high^CD34^+^CD38^-^ populations. We have now revised the text on page 12, which now reads, “In concordance with our findings in AML cell lines, the protein levels of Ki67 were significantly reduced and the protein levels of CXCR4 were increased in CD69^high^CD34^+^CD38^-^ HSC-like populations from primary AML patients (Additional file 1: Fig. S7a-c).”.

Importantly, Ki67 serves as a clinically used proliferation marker for assessing cell cycling status in multiple types of cancer. Ki67 is constitutively expressed in cycling cells (G1, S, G2, and M phases of the cell cycle) but is absent in quiescent cells (G0 phase).

CCND1 and CDK6 are important regulators in the specific phase of cell cycle progression. It is noteworthy mentioning that the reduction of CCND1 and CDK6, as depicted in figure 8, was supported by single-cell defined *CD69*^+^ HSC-like subpopulation in primary AML patients (Fig. 5a, f, and Fig. S5a) and *CD69*-overexpressing AML cell lines (Fig. 6b). However, we only observed a trend of reduced CDK6 levels in the CD69^high^CD34^+^CD38^-^ patient population, without reaching statistical significance (Fig. 6b). It is possible that this may be due to limited patient samples and the lack of refined sorting strategies for isolating a single-cell defined *CD69*^+^ HSC-like subpopulation. The sorted CD34^+^CD38^-^ population is enriched for HSC-like cells but also possibly coexist with cells resembling MPPs and/or LMPPs [2011, Cancer Cell]. Nevertheless, we have removed "CCND1 and CDK6" from the text related to protein expression levels in primary CD69^high^CD34^+^CD38^-^ cells on page 12. Furthermore, in Fig. 8 working model, we specified that the reduction of CDK6 and CCND1 was supported by single cell transcriptomic data of *CD69*^+^ HSC-like subpopulation (Fig. 5a, f, and Fig. S5a) and protein data of two *CD69*-overexpressing cell lines (Fig. 6b). The figure legend in Fig. 8 now reads, “Among them, HSC-like leukemic cells characterized by the surface marker CD69 possessed chemoresistant capacity possibly via the CD69-mTOR axis. Suppression of the mTOR signaling pathway, in the *CD69*^+^ HSC-like cell subpopulation and *CD69*-overexpressing cell lines, might lead to cell quiescence via suppression of cell cycle regulators CCND1 and CDK6 (as shown in Fig. 5a, 6b, and Fig. S5a) and cell adhesion to stromal cells such as MSCs through the CXCR4-CXCL12 interaction (as shown in Fig. 6d, f).”.

Reference: Goardon N, Marchi E, Atzberger A, et al. Coexistence of LMPP-like and GMP-like leukemia stem cells in acute myeloid leukemia. Cancer Cell. 2011 Jan 18;19(1):138-52. doi: 10.1016/j.ccr.2010.12.012.

3) The authors have improved the text and figures, addressing majority of my previous concerns regarding clarity. In part, it is still a struggle to follow what is written vs shown in figures:

- The data from malignant cell fraction by mutation or LAIP gene expression (figS2g) is provided to compare to the prediction. However, as it shown separately as summarized percentages (that also are not correlated to each other), it is very difficult to judge the matching to cell assignment.

**Response:** The qualitative comparison between leukemic cells defined by our prediction and by mutation/LAIP was shown in Fig. S2c, while Fig. S2g aimed to represent the proportion of mutated and LAIP-coexpressing cells detected in pre- and post-therapy leukemic cells, respectively. We have now added statistical labels in Fig. S2c to simultaneously provide a quantitative comparison. The improved Fig. S2c has the capability to directly confirm that the prediction accuracy of malignant cells has reached approximately 95%.

The revised figure legend of Fig. S2c in Additional file 1: “UMAP plots showing the clustering of paired pre- and post-therapy samples from each patient with healthy donors. Transcriptionally predicted leukemia cells (left), mutant cells (middle), and LAIP-coexpressing cells (right) are highlighted, respectively. The numbers of highlighted cells are shown. Of the highlighted cells, the percentage of cells transcriptionally predicted to be leukemic is shown in parentheses.”

- The case numbering is not consistently used to point the reader to the respective result panels. It takes a long time to read text, explore the figure and try to follow the rationale for subsets of data shown. This especially complicates interpretations related to figs 3 and 4 where further sub-populations (e.g. HSC-like that remain or disappear during treatment) are discussed. It would be important to pay attention to labelling the figure panels with additional clarifying text (dataset, or subset) and include the patient identifiers belonging to each subset at first mention in text to improve clarity across the paper.

**Response:** Following the reviewer’s suggestions, we have made changes where needed across the whole manuscript. These changes include: (1) We added the patient identifiers at first mention in text on pages 5, 6, 7, 8, 9, 13, figure legends/titles for Fig. 2f,g, Fig. 3e, Fig. 4, Fig. 5a, Fig. S2e, Fig. S3e, and Fig. S4e,f on pages 19-21 and in Additional file 1, which are highlighted with underscores as shown below. (2) We added labels to indicate the grouping of patients and populations without LSC and OXPHOS signatures, as well as added a line-plot to display decrease in cellular diversity in response to treatments, to match the text better (improved Fig. S4e). (3) We added the labels indicating the cohort or patient subsets to enhance the self-interpretation of figures (improved Fig. 2b,c, Fig. 7d,g,h, Fig. S2a,e,h, Fig. S4c,e, Fig. S8e, and Fig. S9e,f,g).

The revised text on page 5: “Noticeably, in three patients with identifiable post-therapy malignant cells (AML7070B, AML328, and AML329), a small proportion of post-therapy cells coclustered with pre-therapy AML cells (Additional file 1: Fig. S2a).”

The revised text on page 6: “… in leukemic cells from three patients (P115, P116, and P119; Fig. 2f). Additionally, in two patients (P105 and P115) who harbored a Y chromosome deletion... Notably, three patients (P105, P115, and P116) were transcriptionally predicted to have more than 100 post-treatment leukemic cells.”

The revised text on page 7: “Cells coexpressing LAIP markers were identified using the scRNA-seq data of these eleven patients.”

The revised text on page 8-9: “In contrast, two (P116 and P105) out of three patients who achieved partial remission (PR) maintained diverse cell types …. In contrast, two (P116 and P105) out of seven patients whose pre-therapy HSC-like cell populations carried LSC signatures …, and half (three out of six; P116, P105, and P124) of patients whose diagnostic LMPP-like populations carried an active OXPHOS signature .… Three patients (P105, P115, and P116) who had hundreds of cells ... E/B/M-like cells in P115 exhibited an increased transcriptional activity … Notably, post-therapy AML cells from patients who achieved partial remission (P116 and P105) displayed activation of … We further focused on the seven patients (out of thirteen; P116, P105, P122, P106, P119, P118, and P115) whose HSC-like populations possessed LSC signatures. The HSC-like populations persisted after therapy in two (P116 and P105) of the seven patients (Fig. 3d, 4d).”

The revised text on page 13: “Notably, in our scRNA-seq cohort, the *PTPN11* mutation was only present in the resistant patient (P105) ...”

The revised figure legend for Fig. 2f on page 19: “… from four patients (P115, P116, P119, and P120) harboring this chromosomal translocation.”

The revised figure legend for Fig. 2g on page 19: “… from two patients (P105 and P115) with a chromosome Y deletion.”

The revised figure legend for Fig. 3e on page 20: “… leukemic populations in three representative patients (P116, P105, and P122).”

The revised figure title for Fig. 4 on page 20: “Dynamic cellular and transcriptomic changes in leukemic cells after chemotherapy in P116, P105, and P115”

The revised figure legend for Fig. 5a on page 21: “…pre-therapy HSC-like populations between resistant cases (P105 and P116) and sensitive cases (P115, P106, P118, P119, and P120).”

The revised figure legend for Fig. S2e in Additional file 1: “… Post-therapy samples from P116, P105, and P124 are indicated.”

The revised figure legend for Fig. S3e in Additional file 1: “Flow cytometry plots show the gating of pre-therapy bone marrow cells derived from AML patients (P114, P118, and P120) and their expression of stem and progenitor markers (CD34 and CD117).”

The revised figure legend for Fig. S4e in Additional file 1: “ … (Right) Line plot showing the changes in leukemic cellular diversity. Three patients (P116, P105, and P106) who achieved partial remission (PR) are indicated.”

The revised figure legend for Fig. S4f in Additional file 1: “UMAP visualization of pre-therapy HSC-like leukemic cells from resistant patients (P116 and P105) and sensitive patients (P122, P106, P119, P118, and P115).”

- Unclear what is shown in figS2d and fig 3e data shown not matching text

**Response:** Fig. S2d shows the proportion of cells derived from pre-therapy sample in each cell cluster. We used a commonly used approach in published study by Jeffrey M. Granja, et al., 2019, in which a cluster was classified as leukemic if it had a high proportion (at least 80%) of cells derived from the pre-therapy sample, or normal if it had a low proportion. To clarify the figure, we have made changes to its legend in Additional file 1. It now reads, "Proportion of cells from the pre-therapy sample in each cell cluster for individual patients. A point represents a cluster. Clusters for each patient were defined by clustering scRNA-seq data shown in panel c and Fig.2b." We have also moved its citation to a more appropriate position on page 5, which now reads, “We defined a cluster as leukemic if more than 80% of the cells in this cluster were derived from pre-therapy samples and exhibited a close relationship with myeloid cells, while cells in the remaining clusters were classified as normal cells (see “Methods”, Additional file 1: Fig. S2d)”.

For Fig.3e (and similar Fig. S4b), we have added gene labels (e.g. *CD34*, *ERG*, *SLC25A1*, and *MRPS34*) to match the data in the figure with the main text on page 8. Meanwhile, we have also revised the legends to clearly interpret these two figures on page 20 and Additional file 1.

The revised figure legend for Fig. 3e on page 20: “Heatmaps showing expression fold changes (FC) of core enriched genes (columns) contributing to LSC and OXPHOS signatures in each HSPC-like population (rows) compared to all other leukemic populations in three representative patients (P116, P105, and P122). Core enriched genes are identified from GSEA results and those related to cell stemness and metabolism are indicated.”

The revised figure legend for Fig. S4b in Additional file 1: “Heatmaps showing expression fold changes (FC) of core enriched genes (columns) contributing to LSC and OXPHOS signatures in each HSPC-like population (rows) compared to all other leukemic populations within each patient. Core enriched genes are identified from GSEA results and those related to cell stemness and metabolism are indicated.”

Reference: Granja JM, Klemm S, McGinnis LM, et al. Single-cell multiomic analysis identifies regulatory programs in mixed-phenotype acute leukemia. Nat Biotechnol. 2019;37(12):1458-1465. doi:10.1038/s41587-019-0332-7.

- There are also language issues (grammar) that should be addressed to improve clarity of text

**Response:** The revised manuscript has been edited by professional English editors from Springer Nature Author Services. The revised manuscript improves the overall clarity and flow of the message.

Given that this study has focused on the more rare pediatric AML, the differences between pediatric vs adult AML are surprisingly very little discussed throughout the study. As similar work characterizing post-treatment adult AML is most likely focus in several ongoing and future studies, this aspect could be the more unique feature of the present work.

**Response:** Thank for the reviewer’s comments. We have now discussed the similarity and difference between pediatric and adult AMLs on pages 14 and 17.

The revised text on page 14: “Using mouse models transplanted with AML cells from adult patients, previous studies have revealed four chemoresistant features [6-8, 23, 24]. Building upon these findings, we found that in both pediatric and adult AML patients, LSC and OXPHOS expression signatures are mapped onto HSPC-like leukemic populations and are the two major resistance features…”

The revised text on page 17: “In extension of these previous studies, our work showed that transcriptomically defined *CD69*^+^ HSC-like cells possess chemo-resistance capacity in pediatric and adult AML patients. Interestingly, we also noted several potential differences between pediatric and adult AML. First, while a high proportion of the *CD69*^+^ HSC-like subpopulation (*CD69*^+^-high) could be used to further stratify the well-known LSC expression scoring-based patient survival, the grouping of patients affected by *CD69*^+^-high was different. Specifically, *CD69*^+^-high could further divide the LSC-defined low-risk group of adult patients, while *CD69*^+^-high led to further stratification of the high-risk group of pediatric patients (Fig.7d, Additional file 1: Fig. S9d). These differential prognostic values may reflect the distinct stem cell biology associated with specific age groups, such as the self-renewal capacities of LSCs, as well as interactions of LSCs with the BM niche and the immune system [48-50]. Second, the presence of *CD69*^+^-high was associated with specific genomic alterations. Several alterations were common between pediatric and adult AML, including trisomy 8 in the *CD69*^+^-high group and *CBFB-MYH11* fusions in the *CD69*^+^-low group (Fig.7d, Additional file 1: Fig. S9d). In addition, some genomic alterations enriched in the *CD69*^+^-high group were distinctive between pediatric and adult AML. Mutations in *TP53* and the cohesin genes were observed in the *CD69*^+^-high group of adult AML, while they were nearly absent in that of pediatric AML (Fig. 7d, Additional file 10: Table S9). Furthermore, different sets of unusual fusion genes in the *CD69*^+^-high group were observed between pediatric and adult AML (Fig.7d, Additional file 1: Fig. S9d). These findings are consistent with the distinct genomic landscape reported in pediatric and adult AML patients [51, 52]. Nevertheless, further studies are warranted to confirm our observations and to explore the underlying regulatory mechanisms.”

Reviewer #2: The authors have addressed all my comments.

**Response:** We thank the reviewer to have pointed out that we addressed all his/her comments.

**Third round of review**

**Reviewer 1**

The authors have included further experimental data and clarified the analyses performed. I have no further comments.
